# Biomarker signatures of aging

**DOI:** 10.1111/acel.12557

**Published:** 2017-01-06

**Authors:** Paola Sebastiani, Bharat Thyagarajan, Fangui Sun, Nicole Schupf, Anne B. Newman, Monty Montano, Thomas T. Perls

**Affiliations:** ^1^ Department of BiostatisticsBoston University School of Public Health801 Massachusetts AvenueBostonMA02118USA; ^2^ Department of Laboratory Medicine and PathologyUniversity of Minnesota Medical SchoolMMC 609 Mayo420 DelawareMinneapolisMN55455USA; ^3^ Department of EpidemiologySergievsky CenterColumbia University Mailman School of Public Health630 West 168th StreetNew YorkNY10032USA; ^4^ Department of EpidemiologyUniversity of PittsburghA527 Crabtree Hall130 DeSoto StreetPittsburghPA15261USA; ^5^ Department of MedicineHarvard Medical SchoolBrigham and Women's Hospital221 Longwood AvenueBostonMA02115USA; ^6^ Department of MedicineGeriatrics SectionBoston University School of Medicine and Boston Medical CenterRobinson 240088 E Newton StBostonMA02118USA

**Keywords:** biological aging, biomarkers, healthy aging, morbidity and mortality

## Abstract

Because people age differently, age is not a sufficient marker of susceptibility to disabilities, morbidities, and mortality. We measured nineteen blood biomarkers that include constituents of standard hematological measures, lipid biomarkers, and markers of inflammation and frailty in 4704 participants of the Long Life Family Study (LLFS), age range 30–110 years, and used an agglomerative algorithm to group LLFS participants into clusters thus yielding 26 different biomarker signatures. To test whether these signatures were associated with differences in biological aging, we correlated them with longitudinal changes in physiological functions and incident risk of cancer, cardiovascular disease, type 2 diabetes, and mortality using longitudinal data collected in the LLFS. Signature 2 was associated with significantly lower mortality, morbidity, and better physical function relative to the most common biomarker signature in LLFS, while nine other signatures were associated with less successful aging, characterized by higher risks for frailty, morbidity, and mortality. The predictive values of seven signatures were replicated in an independent data set from the Framingham Heart Study with comparable significant effects, and an additional three signatures showed consistent effects. This analysis shows that various biomarker signatures exist, and their significant associations with physical function, morbidity, and mortality suggest that these patterns represent differences in biological aging. The signatures show that dysregulation of a single biomarker can change with patterns of other biomarkers, and age‐related changes of individual biomarkers alone do not necessarily indicate disease or functional decline.

## Introduction

The steady increase in human average life expectancy in the 20th century is considered one of the greatest accomplishments of public health. Improved life expectancy has also led to a steady growth in the population of older people, age‐related illnesses and disabilities, and consequently the need for prevention strategies and interventions that promote healthy aging. A challenge in assessing the effect of such interventions is ‘what to measure’. Chronological age is not a sufficient marker of an individual's functional status and susceptibility to aging‐related diseases and disabilities. As has been said many times by Gerontologists and Geriatricians, people can age very differently from one another. Individual biomarkers show promise in capturing specificity of biological aging (Karasik *et al*., 2005), and the scientific literature is rich in examples of biomarkers that correlate with physical function, anabolic response, and immune aging (Gruenewald *et al*., 2006; Walston *et al*., 2006; Stenholm *et al*., 2010; Banerjee *et al*., 2011; Franceschi & Campisi, 2014; Bürkle *et al*., 2015; Cohen *et al*., 2015; Catera *et al*., 2016; Peterson *et al*., 2016). However, single biomarker correlations with complex phenotypes that have numerous and complex underlying mechanisms is limited by poor specificity.

Moving from a simple approach based on one biomarker at a time to a systems analysis approach that simultaneously integrates multiple biological markers provides an opportunity to identify comprehensive biomarker signatures of aging (Zierer *et al*., 2015). Analogous to this approach, molecular signatures of gene expression have been correlated with age and survival (Kerber *et al*., 2009; Passtoors *et al*., 2013), and a regression model based on gene expression predicts chronological age with substantial accuracy, although differences between predicted and attained age could be attributed to some aging‐related diseases (Peters *et al*., 2015). The well‐known DNA methylation clock developed by Horvath has been argued to predict chronological age (Horvath, 2013). Alternative approaches that aggregate the individual effects of multiple biological and physiological markers into an ‘aging score’ have also been proposed (MacDonald *et al*., 2004; Levine, 2013; Sanders *et al*., 2014; Belsky *et al*., 2015; Peterson *et al*., 2016). These various aging scores do not attempt to capture the heterogeneity of aging. In addition, many of these aging scores use combinations of molecular and phenotypic markers and do not distinguish between the effects and the causes of aging (Newman, 2015).

Here we propose a system‐type analysis of 19 circulating biomarkers to discover different biological signatures of aging. The biomarkers were selected based upon their noted quantitative change with age and specificity for inflammatory, hematological, metabolic, hormonal, or kidney functions. The intuition of the approach is that in a sample of individuals of different ages, there will be an ‘average distribution’ of these circulating biomarkers that represents a prototypical signature of average aging. Additional signatures of biomarkers that may correlate to varying aging patterns, for example, disease‐free aging, or aging with increased risk for diabetes or cardiovascular disease (CVD), will be characterized by a departure of subsets of the circulating biomarkers from the average distribution. We implemented this approach using data from the Long Life Family Study (LLFS), a longitudinal family‐based study of healthy aging and longevity that enrolled individuals with ages ranging between 30 and 110 years (Newman *et al*., 2011; Sebastiani *et al*., 2013). We also validated the predictive values of the signatures discovered in LLFS using data from the Framingham Heart Study (FHS). Figure S1 (Supporting information) summarizes the overall discovery and replication analysis.

## Results

The LLFS is a family‐based study that enrolled 4935 participants including probands and siblings (30%), their offspring (50%), and spouses (20%), with ages between 30 and 110 years (Newman *et al*., 2011). Approximately 40% of enrolled participants were born before 1935 and had a median age at enrollment of 90 years and 45% participants were male (Fig. S2). Almost 55% of participants from the proband generation (birth year < 1935) have died since enrollment, with a median age at death of 96 years. Mortality in the generation born after 1935 is lower (3%) and among these few that have died, median age at death is currently 69 years. Table S1 (Supporting information) describes participants’ characteristics.

### Generation of biomarker signatures in LLFS

Approximately 40 serum biomarkers were measured at enrollment in 4704 LLFS participants, and the quality and distribution of these biomarkers has been characterized elsewhere (Sebastiani *et al*., 2016b). After removal of biomarkers that did not change with age, age and sex standardized values of 19 uncorrelated biomarkers (Table 1) were analyzed with an agglomerative cluster analysis that identified 26 significant clusters of LLFS participants (*P *< 0.004, Fig. S3). Seventeen of these clusters had at least 10 individuals, and only eight clusters had more than 40 individuals. Means and standard deviations of the 19 biomarkers in each cluster defined the 26 biomarker signatures that are depicted in Table S2 (Supporting information). The dependency of clusters on all 19 biomarkers was tested by ‘leave one‐biomarker‐out’ replication (Supporting information and Table S3).

**Table 1:** Biomarkers used for generation of signatures, and change with older age

High‐sensitivity C‐reactive protein (hsCRP) ↑ Interleukin 6 (IL‐6) ↑ N‐terminal B‐type natriuretic peptide (NT‐proBNP) ↑ Absolute monocyte count (Abs.M) ↑ White blood cell counts (WBC) ↑	Inflammation biomarkers
Red blood cell distribution width (RDW) ↑ Transferrin receptor (Transf.R) ↑ Mean corpuscular volume (MCV) ↑ Hemoglobin (Hgb) ↑	Hematological biomarkers
Glycated hemoglobin (HbA1c) ↑ Soluble receptor for advanced glycation end product (sRAGE) ↑ Adiponectin (Adip) ↑ Insulin‐like growth factor (IGF1) ↓	Diabetes associated biomarkers
Total cholesterol (T.Chol) ↑ ↓	Lipid biomarker
Sex hormone‐binding globulin (SHBG) ↑ Dehydroepiandrosterone sulfate (DHEA) ↓	Endocrine biomarkers
Albumin (Album) ↓ Creatinine ↑ Cystatin C ↑	Renal biomarkers

List of the 19 biomarkers used to define signatures. The biomarkers are grouped by functions. Biomarkers with generally increasing values with older age are labeled with an arrow pointing up, while biomarkers that generally decrease with older age are labeled with an arrow pointing down.

**Figure 1:**
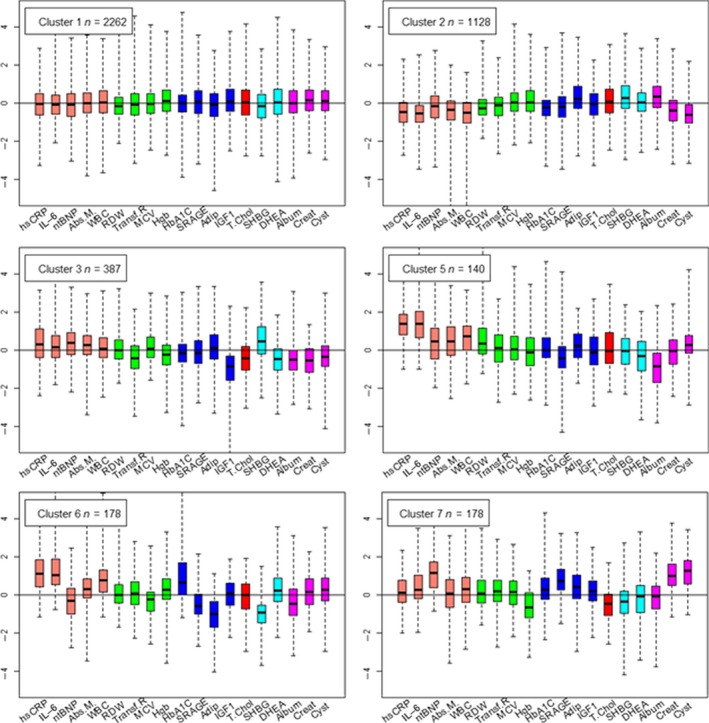
Six signatures of 19 biomarkers. Side‐by‐side boxplots display the distributions of the 19 standardized biomarkers in LLFS participants allocated to each cluster. Biomarkers are grouped and colored by function (salmon: inflammation; green: anemia; blue: diabetes; red: lipid; cyan: endocrine; magenta: renal). In each plot, the horizontal line at 0 represents the expected values of the standardized biomarkers, and hence, the value of biomarkers expected for specific age and sex groups. Note that the unstandardized values change with age. For example, the expected value of albumin and hemoglobin for a male aged between 60 and 65 years would be 4.2 g dL^−1^ and 15 mg L^−1^, respectively, while the expected value of albumin and hemoglobin for a male aged between 80 and 85 years would be 3.9 g dL^−1^ and 14.5 mg L^−1^, respectively (Fig. S2). LLFS, Long Life Family Study.

**Figure 2:**
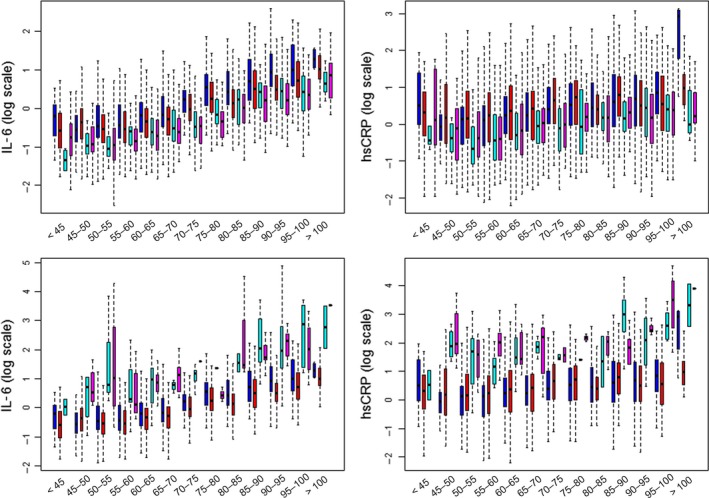
Age‐ and sex‐specific distribution of IL‐6 and hsCRP in LLFS participants, by cluster. Top: Age and sex distribution of IL‐6 in LLFS participants in cluster 1 (blue = males, red = females), and cluster 2 (cyan = male, magenta = females). Both inflammation markers are lower in cluster 2 than cluster 1 for all age groups. *Bottom:* Age and sex distribution of IL‐6 in LLFS participants in cluster 1 (blue = males, red = females), and cluster 5 (cyan = male, magenta = females). Both inflammation markers are substantially more elevated in cluster 5 than cluster 1 for all age groups. hsCRP, high‐sensitivity C‐reactive protein; LLFS, Long Life Family Study.

Figure 1 shows the distribution of the 19 biomarkers in the six largest clusters, and the complete description of the 26 clusters is in Figs S5–S17. In each plot in Fig. 1, the horizontal line at 0 represents the value of standardized biomarkers expected for an individual age and sex group. We designate the biomarker signature associated with cluster 1 as the ‘referent signature’ which is characterized by age and sex standardized biomarkers symmetrically distributed around 0. On average, all biomarkers of the individuals allocated to this cluster would match the values expected for their age and sex group. Note, however, that unstandardized values change with age and sex, as shown in Fig. 2 for IL‐6 and CRP, and Fig. S18a–p for all 19 biomarkers. Cluster 1 included 37% of participants born < 1935 (*n *= 837), with median age 90 years, and 54% females. Cluster 2 included 1128 LLFS participants who were slightly older than participants in cluster 1 (41% born < 1935; median age at enrollment 91 years, and 59% born ≥ 1935, median age at enrollment 61 years) and with a higher rate of females (55%). The signature associated with the second cluster was characterized by lower than average creatinine and cystatin values, lower than average biomarkers of inflammation, and elevated albumin suggesting that individuals in this cluster had lower than average inflammation and better than average kidney function for all ages. Cluster 3 included 387 individuals with noticeably low IGF1, and DHEA, and elevated sex hormone‐binding globulin (SHBG) and markers of inflammation, with an overall signature of poor physical function. Clusters 5 and 6 had similar patterns of biomarkers with the exception of NT‐proBNP, HbA1C, adiponectin, and SHBG. Other clusters were characterized by more substantial variations of biomarkers but smaller sample sizes. Table S4 (Supporting information) summarizes participants’ demographics by all cluster.

### Annotation of biomarker signatures in LLFS by their predictive values

**Figure 3:**
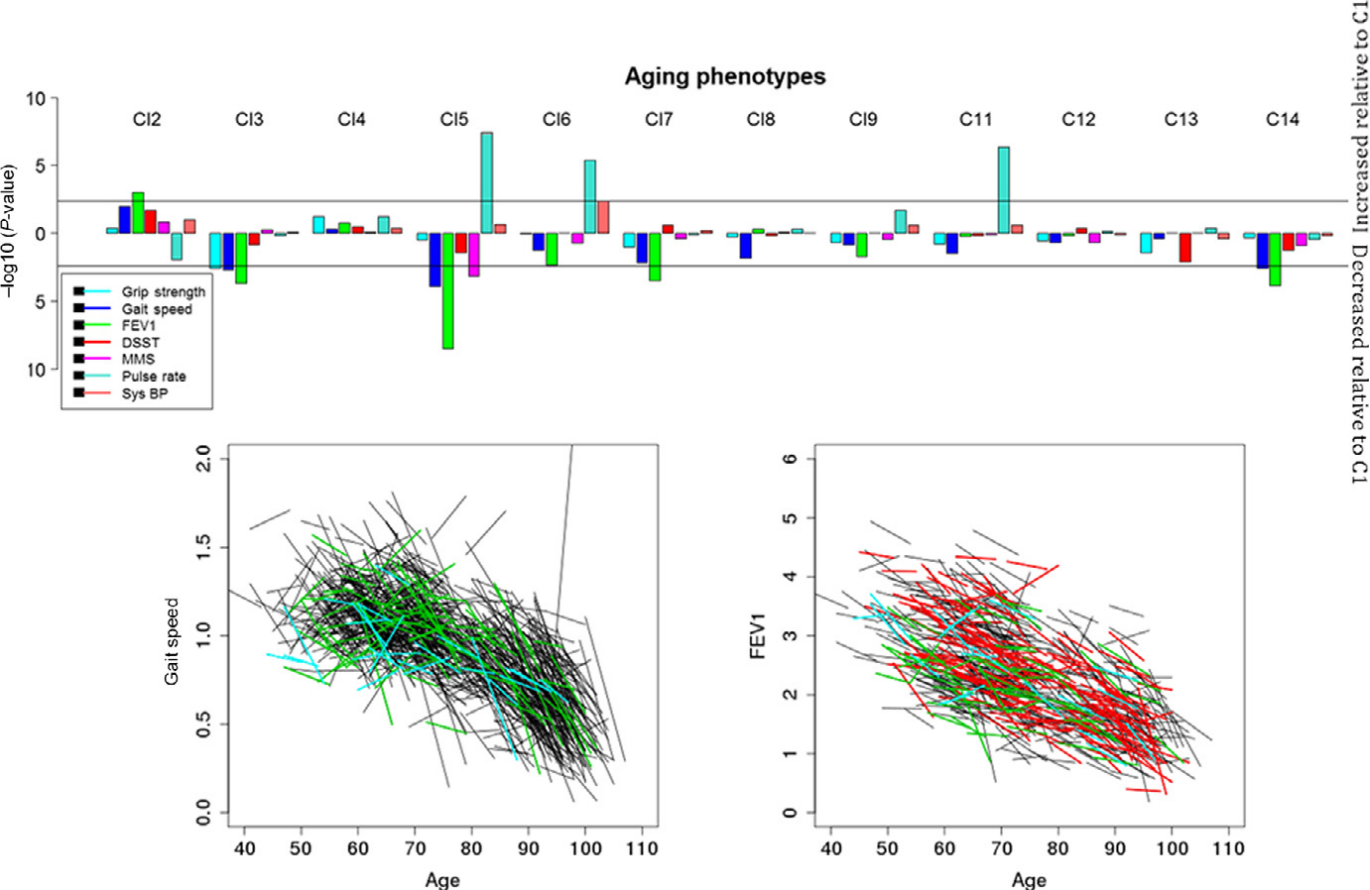
Association between biomarker signatures defined by 10 or more participants and physiological markers of aging. Top panel: Manhattan plot of the −log(*P*‐value) to test significant differences between physiological markers of aging comparing clusters with more than 20 subjects relative to cluster 1 (the referent group). Phenotypes are grip strength, gait speed, forced expiratory volume in 1 s (FEV1), scores of digital symbol substitution test (DSST) and Mini‐Mental State Examination (MMSE), pulse rate, and systolic blood pressure (sys BP). Horizontal lines represent the significance threshold based on Bonferroni correction. Bars above 0 represent increased effects relative to cluster 1, while bars below 0 represent decreased effects relative to cluster 1. For example, participants in cluster 2 have better gait speed, FEV1, DSST and MSE, and slower pulse rate compared to cluster 1, although only the difference of FEV1 remains significant after Bonferroni correction. Participants in cluster 5 have significantly slower gait speed, significantly lower FEV1 and MMSE, and faster pulse rate compared to cluster 1. Estimates of all 84 comparisons and p‐values are in Table S2. *Bottom panel:* Scatter plots of individual changes of gait speed and FEV1 between enrollment and the second in‐home visit colored by cluster membership (red: cluster 2; green: cluster 3; cyan: cluster 5). Each segment represents an individual change between age at enrollment and age at visit 2.

To test whether the biomarker signatures for clusters with 10 or more individuals correspond to different patterns of biological aging, we correlated them with longitudinal changes in physiological functions and incident risk of cancer, CVD, type 2 diabetes, and mortality. Figure 3 and Table S5 (Supporting information) summarize the results of the analysis that compared participants in cluster 1 (*n* = 2262) with the other clusters in terms of aging phenotypes measured at enrollment and at the second visit (approximately 8 years apart). The analysis showed that the different signatures are characterized by significant variations in important physiological markers of aging that include grip strength (significantly worst in cluster 3 compared to cluster 1), gait speed (significantly slower in clusters 3, 5, and 14 compared to cluster 1), FEV1 (significantly worse in clusters 3, 5, 7, and 14 compared to cluster 1), cognitive functions (significantly worse in cluster 5 compared to cluster 1), and pulse pressure (significantly higher in clusters 5, 6, and 11 compared to cluster 1). Participants in the 2nd cluster were characterized by a faster gait speed, higher FEV1, better cognitive scores, and lower pulse rate compared to the referent cluster. The difference in FEV1 was statistically significant (Bonferroni corrected significance 0.004), while the significance level for the other markers did not pass correction for multiple comparisons. No substantially significant interactions between age and clusters were found suggesting that physiological advantages or disadvantages remained constant over the past 8 years. However, this result may change as more data are collected and power to detect significant interactions increases.

Table 2 shows the results of the prospective analysis of morbidity and mortality in about 8 years of longitudinal follow‐up based on a Cox proportional hazard model stratified by sex and adjusted for age at enrollment. Several signatures showed significantly different hazards for mortality and morbidity compared to the referent group. Consistent with the favorable signature of lower inflammation and better kidney function, subjects in cluster 2 were characterized by significantly lower risk for type 2 diabetes and overall mortality compared to participants in cluster 1, while all other clusters were characterized by significantly higher risk for mortality, or aging‐related diseases including cancer, CVD, and type 2 diabetes. Participants in cluster 2 had a significantly lower BMI compared to participants in cluster 1 (average BMI difference −1.54, 95% CI: −1.82; −1.257), while participants in cluster 6 had a significantly higher BMI compared to cluster 1 (average BMI difference 2.87, 95% CI: 2.32; 3.47). Some of the smaller clusters had a limited number of events, so the results are less reliable. Note that 200 of 330 CVD events were a ‘first time event’, and 289 of these events were not preceded by type 2 diabetes.

**Table 2:** Prediction of incident morbidity and mortality in LLFS

Trait	Cluster	Size	*n* events	HR	Wald's Test	*P* value
Death	2	1128	231	0.81	6.72	0.009524
Type 2 diabetes			11	0.51	3.91	0.048026
Death	3	387	96	1.24	14.13	0.000171
Peripheral artery disease			6	1.73	5.03	0.024986
Glaucoma			8	1.53	4.20	0.040338
Death	5	140	49	1.20	22.68	1.91E‐06
Type 2 diabetes			6	1.32	6.47	0.010941
Type 2 diabetes	6	178	12	1.32	17.56	2.79E‐05
Death	7	178	73	1.10	21.12	4.31E‐06
CVD			19	1.10	5.38	0.020357
Cancer	9	31	4	1.16	5.11	0.023814
Death	11	33	7	1.12	8.04	0.004587
COPD	12	91	3	1.15	5.78	0.016201
CVD	13	32	6	1.15	15.45	8.47E‐05
Death			17	1.05	5.61	0.017905
Death	14	28	12	1.11	20.01	7.70E‐06
Skin cancer			3	1.11	5.41	0.020031
Death	15	11	6	1.15	20.47	6.06E‐06
Death	16	14	9	1.08	11.76	0.000606
Skin cancer	17	11	4	1.22	33.35	7.72E‐09
Cancer			2	1.13	7.31	0.006866

All hazard ratios (HR) are relative to the referent signature of cluster 1 and were estimated using Cox proportional hazard and were adjusted by age at enrollment and stratified by sex. *P*‐values to test whether the HR are different from 1 are based on Wald's test. Cardiovascular disease (CVD) was defined as myocardial infarction, coronary artery bypass graft, atrial fibrillation, congestive heart failure, or valve replacement.

### Calibration of biomarker data in Framingham Heart Study

Our goals with the FHS data were (i) to verify whether the age‐sex distributions of biomarkers in the LLFS were similar to those observed in the LLFS, (ii) to use the biomarker data available in the FHS to predict their biomarker signatures, and (iii) to use these predicted signatures to validate their association with morbidity and mortality in FHS. Limited subsets of biomarkers were available in the three FHS cohorts, and the most complete subset was from the offspring generation with 12 biomarkers measured at exam 7 and WBC measured at exam 2 that we used as a proxy of the measurement at exam 7 (Table S6). Analysis of one biomarker at a time showed that some markers of inflammation [high‐sensitivity C‐reactive protein (hsCRP), NT‐proBNP, IL‐6, WBC], diabetes (IGF1, HBA1C, SRAGE), endocrine functions (SHBGE), and kidney function in men (Albumin) had significantly different distributions in age groups after adjusting for laboratory‐specific effects (Table S7 and Fig. S19). To remove laboratory‐specific effects, calibration of the biomarkers in the FHS was performed for the FHS original and offspring generations as described in the Appendix S1, and histograms of the externally standardized biomarker data in FHS in Fig. S20 show that all biomarkers had been calibrated successfully.

### Validation of predictive values of biomarker signatures in FHS

The next step was to develop a classification model and use it to assign one of the 26 biomarker signatures discovered in LLFS to each FHS participant, based on their biomarker profile. To this end, we trained a Bayesian classifier in LLFS data to predict the biomarker signatures of FHS participants, and we evaluated sensitivity and positive predictive values (PPV) when incomplete biomarker data are used for the prediction. Tables S8 and S9 (Supporting information) show the sensitivity and %PPV of the classifier in the LLFS data. Notwithstanding the challenge of a 26‐label classification, the sensitivity ranging between 36% and 100% was substantially higher than a random classification of 26 labels which yields a sensitivity of 3% (Table S8). The %PPV rate was also above 50% for 14 of the 26 clusters, and above 30% for 22 of 26 clusters (Table S9). The PPVs of clusters 9 and 17 were lower, suggesting that results associated with these clusters may not be reliable. Both sensitivity and %PPV were also tested using the subsets of the biomarkers available in the original and offspring generations of the FHS (Tables S10 and S11). With just the eight biomarkers available in the FHS original generation, the %PPV decreased substantially, while maintaining acceptable values (%PPV > 25%) with the 13 biomarkers used in the FHS offspring generation.

When the classifier trained with LLFS data was applied to FHS standardized data, it predicted the most likely biomarker signature of each FHS participant. The distribution of these predicted signatures in FHS participants from the offspring generation was substantially different from LLFS, and particularly, the signature associated with healthier aging (cluster 2) was less common in FHS participants (Fig. S21). In both FHS generations, the incident risk for mortality, CVD, and diabetes of participants with signatures 2 through 26 was compared to the referent group (signature 1). Table 3 reports the analysis of risk for mortality in the FHS offspring for the nine clusters that had a significantly different hazard for mortality compared to the referent cluster in LLFS, while the complete set of results is in Table S12. Six of the nine clusters replicated the significantly different hazard for mortality relative to the referent cluster, with the same effects seen in LLFS. The other three clusters had increased risk for mortality but failed to reach statistical significance. Noticeably, FHS offspring with signature 2 also had a reduced risk for mortality compared to the referent group, confirming the favorable effect of a signature combining lower than average inflammation and better than average kidney functions. The reduced risk for mortality associated with signature 2 and the increased risk for mortality of signature 14 were also replicated in FHS participants of the original cohort (Cluster 2: HR = 0.64, *P* < E10^−4^, Cluster 14: HR = 1.05, *P*‐value 0.007). The increased risk for CVD of signature 5 was replicated in the offspring generation, and the reduced risk for type 2 diabetes of signature 2 and the increased risk for type 2 diabetes of signature 6 were replicated in both original and offspring generations (Table 4 and Table S13 and S14).

**Table 3:** Replication of mortality risk by biomarker signatures in FHS offspring

Cluster (signatures)	FHS: offspring						LLFS				Comment
	Size	Events	HR	*P*‐value	%PPV	% →C1	Size	Events	HR	*P*‐value	
1	694	158			78						
2	576	92	0.68	0.02405	56	38	1128	231	0.81	0.00952	Replicated
3	310	56	1.12	0.22662	42	33	387	96	1.24	0.00017	Consistent
5	171	48	1.23	0.00003	30	40	140	49	1.20	< 1E‐05	Replicated
7	195	34	1.02	0.58680	32	57	178	73	1.10	< 1E‐05	Consistent
11	172	75	1.05	0.01120	50	21	33	7	1.12	0.00458	Replicated
13	297	35	1.02	0.21334	13	21	32	6	1.05	0.01790	Consistent
14	126	77	1.10	< 5 E‐07	64	7	28	12	1.11	0.00001	Replicated
15	100	18	1.04	0.033	13	19	11	6	1.15	0.00001	Replicated
16	93	64	1.13	< 5 E‐06	45	6	14	9	1.08	0.00061	Replicated

Replication of the risk for mortality relative to the referent group (cluster 1) in the offspring generation of the FHS. Hazard ratios (HR) were estimated using Cox proportional hazard regression and adjusted by age at blood collection and stratified by sex. Significance is based on *P*‐values from Wald's test. %PPV denotes the proportion of positive predicted values of each signature selected by the Bayesian classifier trained in the LLFS data. The column denoted by ‘%→C1’ denotes the proportion of profiles assigned to the referent group.

**Table 4:** Replication of CVD risk by biomarker signatures in FHS offspring

Cluster	FHS: offspring				LLFS				Comment
	Size	No. events	HR	*P*‐value	Size	No. events	HR	*P*‐value	
Cardiovascular disease									
1	694	158			2262	151			
5	171	48	1.23	2.90E‐05	140	14	1.13	0.07283	Replicated
7	195	34	1.02	0.58680	178	19	1.10	0.02036	Consistent
13	297	35	1.02	0.21348	32	6	1.15	8.47E‐05	Consistent
Diabetes									
1	694	53			2262	40			
2	576	31	0.52	0.0080	1128	11	0.51	0.04802	Replicated
5	171	18	1.07	0.3726	140	6	1.32	0.01094	Consistent
6	289	78	1.34	3.3E‐13	178	12	1.32	2.8E‐05	Replicated

Hazard ratios (HR) relative to the referent signature of cluster 1 were estimated using Cox proportional hazard regression, adjusted by age at examination in 7 FHS, and stratified by sex. Significance is based on *P*‐values from Wald's test. Cardiovascular disease (CVD) in FHS was defined as myocardial infarction, coronary insufficiency, and congestive heart failure.

## Discussion

We used an agnostic data‐driven cluster analysis to identify 26 signatures of 19 blood biomarkers associated with important aging‐related physiological functions summarized in Table 1. To demonstrate that these signatures are associated with differences in biological aging, we analyzed their ability to predict changes in physical and cognitive function, survival, and risk of age‐related diseases, including cancer, cardiovascular events, and type 2 diabetes. We showed that 10 of these signatures predict different risks of morbidity and mortality in LLFS participants and we identified one signature that is associated with healthy aging, as characterized by better physical and cognitive function, and reduced risk for mortality and morbidity. The risk prediction of 7 of the 10 signatures was replicated in an independent set of participants from the FHS with consistent effects. Our analysis shows that various signatures of 19 circulating biomarkers exist, and their significant associations with function, morbidity, and mortality suggest that these patterns represent differences in biological aging.

While prediction of the risk for morbidity and mortality was used to demonstrate that different biomarker signatures are linked to different trajectories of aging‐related function, morbidity, and mortality, the goal of our analysis was not to discover another risk score of aging‐related diseases. Many scores have already been described and work well in an epidemiological setting, for example, the physiological index of comorbidities (Newman *et al*., 2008; Newman & Murabito, 2013), the Healthy Aging Index that was developed with LLFS data (Sanders *et al*., 2014), or the well‐known Framingham Risk Score (Wilson *et al*., 1998) and its modifications (Tsao & Vasan, 2015). Our hypothesis was that patterns of departures from the ‘average age trajectories’ of many circulating biomarkers may be linked to differences in biological aging that are characterized by worsened or improved physical and cognitive functions, morbidity, and mortality. Validating the hypothesis, our analysis discovered various signatures of circulating biomarkers that were significantly associated with different risk for disease and death, and varying physical and cognitive functions. Importantly, this hypothesis contrasts with the view that the biological profile of older, but healthy individuals should be indistinguishable from that of younger healthy people (Huemer, 1977), a tenant that is the basis of ‘anti‐aging’ and ‘orthomolecular medicine’. We have previously shown (Sebastiani *et al*., 2016a) that the distributions of the 19 biomarkers change with age in a subset of healthy LLFS participants, and the current analysis provides additional compelling evidence that some age‐related changes in the distribution of biomarkers do not necessarily represent disease. For example, the referent signature associated with cluster 1 captures these average age‐related changes (see hsCRP and IL‐6 in Figs 2 and S18a) and is associated with lower risk for morbidity and mortality than the majority of the other signatures. Signature 2 was associated with better physical and cognitive functions, and lower risks for mortality and type 2 diabetes than the referent signature. Interestingly, levels of markers of inflammation of individuals sharing this signature tended to be higher with older age, but they were lower in all age groups compared to the referent group (Fig. 2, top panel, and Fig. S18b). These results are consistent with some of the data shown in (Belsky *et al*., 2015) that emphasize the need to study aging in younger in addition to older individuals to identify markers of healthy and unhealthy aging.

An important characteristic of these biomarker signatures of ‘biological aging’ is that they are only based on molecular data and do not include any expressed aging‐related phenotype. Investigators have proposed mathematical models to compute the biological age of an individual that use molecular data together with physiological phenotypes such as forced expiratory volume (FEV_1_), body mass index (BMI), grip strength, and systolic blood pressure (Levine, 2013; Belsky *et al*., 2015). While the inclusion of these expressed phenotypes may improve prediction of morbidity and mortality because changes of these expressed phenotypes may be closer in time to onset of morbidity and mortality, signatures based exclusively on molecular data could capture earlier departure from the normal healthy aging trajectory and suggest the need for early interventions before phenotypic symptoms appear.

The graphical display of signatures (Fig.**** 1) underscores the importance of a system‐type approach to assess the clinical implications of altered or dysregulated expression of multiple biomarkers. Our analysis shows that the effect of the dysregulation of a single biomarker changes with varying patterns of other biomarkers. For example, the morbidity and mortality risk associated with elevated hsCRP changes with different patterns of NT‐proBNP and HBA1C (signatures 5 and 6 in Fig. 1). The modeling approach we used, based on clustering, is designed to capture this type of interdependence that would be difficult to describe using standard regression modeling. Such complexity also makes sense given the many different biological mechanisms and their determinants that underlie aging and its many different trajectories (Lopez‐Otin *et al*., 2016). Other system‐type approaches have been proposed to capture the complexity of biological aging (Zierer *et al*., 2015). Li *et al*. used a multisystem approach to model 6 predefined physiological systems and to generate dysregulation scores that correlate with age and predict morbidity and mortality (Li *et al*., 2015). Collino *et al*. (Collino *et al*., 2013) discovered metabolic signatures that can discriminate between different age groups. While these and other analyses showed interesting features of aging, they did not discover different patterns of aging affected by multiple systems simultaneously. Our analysis shows that specific groups of people share specific biomarker signatures that implicate clinical and biological conditions. In the LLFS data, we found 26 such signatures of biomarkers among a group of individuals who are generally aging well and are possibly enriched for genetic and nongenetic factors that promote longevity (Sebastiani *et al*., 2009, 2013; Newman *et al*., 2011).

It is possible that the biomarker signatures discovered in the LLFS are biased toward healthy agers and that we may have missed important biomarkers that correlate with less successful aging. These signatures reproduced well in the offspring cohort of the FHS (Fig. S22a–g), but their distribution in FHS participants was different (Fig. S21) and was characterized by higher prevalence of signatures associated with higher risk for mortality and morbidity. In fact, the FHS did not enroll participants based on longevity or healthy aging and may be considered as a more generalizable sample of aging individuals compared to the LLFS. Noticeably, the FHS has been used to study the genetic and epidemiology of aging‐related diseases with results that have been replicated in numerous other studies. Therefore, the validation of some of the associations of the biomarker signature with morbidity and mortality in the FHS is an important strength of this work (Tsao & Vasan, 2015). We expect that similar analyses in other and larger samples of aging individuals may discover many more signatures that capture additional types of biological aging.

In addition to elucidating different patterns of aging, the signatures we discovered have a potential utility in clinical trials, testing treatments, and nonpharmacological strategies that promote healthy aging or reduce the risk for aging‐related diseases. The predictive values of some of the biomarker signatures suggest that they could become an efficient way to assess the effect of interventions but also a more organic means of simultaneously assessing efficacy and safety of new treatments. Also, biomarker signatures assessed over time will likely detect target‐related outcomes both preclinically and earlier when efficacy for age‐related diseases needs to be demonstrated. If these analyses replicate in larger cohorts, biomarker signatures could be used for patient stratification in the design and analysis of clinical trials and go beyond studies to become tools for early preclinical diagnoses and more efficacious patient treatment in clinical settings.

The selection of biomarkers to be measured in LLFS was based on known or putative roles of the biomarkers in aging and related diseases, and many of these biomarkers have been shown to change differentially in normally aging and healthy aging individuals (Newman *et al*., 2016). By design, there are no ‘surprising’ biomarkers in our signatures because we did not conduct molecular‐wide analyses to discover novel aging‐related biomarkers. An addition limitation of this analysis is that many biomarkers of inflammation such as CMV (Wang *et al*., 2010) that were shown to be important in the calculation of biological aging in (Levine, 2013), or several markers of inflammaging or immunosenescence (Franceschi & Campisi, 2014) have not yet been measured in the LLFS. We expect that many more biomarkers exist that could lead to even more powerful results, and as costs for measuring and processing proteomics data become more approachable and the technology more reliable, these analyses will be very informative. The approach we have used is applicable to high‐throughput data, although large sample sizes will be needed for reliable results.

Rarely do studies of biomarkers use independent data to replicate the findings. In this study, we validated the predictive value of the biomarker signatures in an independent set. Although the validation is suggestive, the lack of complete biomarker data in the FHS (FHS had 13 of the 19 biomarkers) was a limitation, and we had to resort to a proxy measurement of WBC obtained at younger age to avoid loss of specificity. In addition, we are unable to verify whether the signatures predicted by the Bayesian classification rules in the FHS data are valid. The graphical displays of the clusters associated with different inferred signatures suggest the results are correct (Fig. S22a–g), but a definite answer will be provided by a replication with more complete biomarker data.

Our analyses did not attempt to explain why subsets of individuals age more healthily than others, and we hypothesize that both genetic and nongenetic factors contribute to different aging patterns captured by the different biomarker signatures. This work sets the stage for a molecular‐based definition of aging that leverages information from multiple circulating biomarkers to generate signatures associated with different mortality and morbidity risk, and additional work is needed to better characterize these signatures. Application of the proposed approach to larger studies and a larger number of biomarkers will extend the current set of biomarker signatures and possibly discover new ones, and we expect that these biomarker signatures will become a potent investigative, diagnostic, and prognostic tool.

## Experimental procedures

A more detailed description of study samples, data and statistical methods is in Supporting information. Figure S1 (Supporting information) summarizes the analysis process.

### Study populations and study design

The LLFS is a family‐based, longitudinal study of healthy aging and longevity that enrolled 4935 subjects in 583 families between 2006 and 2009 via three American and one Danish field centers (Sebastiani *et al*., 2009, 2013; Newman *et al*., 2011). Surviving participants are currently undergoing a second in‐person evaluation (taking place in 2015–2018). The FHS was recently reviewed in Tsao & Vasan (2015).

### Biomarkers data

Fasting blood samples in LLFS participants were collected as described in Newman *et al*. (2011), and biomarkers to assay were chosen based on known or hypothetical association with aging‐related diseases and functions (Sebastiani *et al*., 2016a). Protocols of biomarkers data in FHS are available from the study web site (https://www.framinghamheartstudy.org/researchers/description-data/index.php).

### Derivation of biomarker signatures

The 19 of the 40 available biomarkers assayed in LLFS that were used for construction of the biomarker signatures appears in Table 1, and preliminary analysis are detailed in Appendix S1. Hierarchical clustering and a novel resampling procedure (Sebastiani & Perls, 2016) were used to detect 26 significant clusters. The distribution of biomarkers for LLFS participants allocated to each cluster was depicted with side‐by‐side boxplots and summarized by means and standard deviations of each biomarker (Fig. 1 and S5–S17, Tables S2 and S4). Sensitivity analysis was conducted to examine the robustness of selected clusters to varying significance levels. To verify the relevance of the 19 biomarkers to define clusters, the analysis was also repeated by removing one biomarker at a time, and differences in clusters were examined. Effect of familiarity was examined as described in the Supporting information.

### Annotation of biomarker signatures

Age‐ and sex‐adjusted mixed effect linear models for repeated measures were used to estimate differences between physiological markers (gait speed, grip strength, FEV1, digital symbol substitution test and Mini‐Mental State Examination, BMI, pulse rate, and systolic blood pressure) associated with the clusters (Fig. 3 and Table S5). Cox proportional hazard models stratified by sex and adjusted by age at enrollment were used for the analyses of incident events (Table 2).

### Calibration of FHS biomarkers

To remove laboratory‐to‐laboratory effect, an external standardization of FHS biomarker data using LLFS standards was used.

### Replication of associations between biomarker signatures and morbidity and mortality in FHS

We built and validated a 26‐label Bayesian classifier (Hand & Yu, 2001) to assign the most likely biomarker signature discovered in LLFS to each FHS participant based on his biomarker data. The accuracy of the Bayesian classifier was evaluated in the LLFS data to assess the goodness of fit of the rule and to estimate the proportion of positive predicted values and the misclassification error. The analyses were also repeated using the subset of biomarkers available in the FHS. The classifier was then used to identify the most likely biomarker signature of FHS participants using the externally standardized biomarker data. In all cases, uniform prior probabilities were used. Incident risk for mortality and morbidity in FHS participants in clusters 2–26 were compared to cluster 1 using Cox proportional hazard regression adjusted for age at blood collection and stratified by sex.

All analyses were conducted in the statistical program r v3 (https://www.r-project.org/) and OpenBUGS (http://www.openbugs.net/w/FrontPage).

## Authors contribution

PS, BT, NS, AN, MM, and TTP performed the study design; BT, NS, AN, and TTP performed the data acquisition; PS and FS performed the statistical analysis; PS, MM, and TTP performed the interpretation of results; PS, FS, MM, BT, NS, AN, and TTP performed the manuscript preparation and revision.

## Conflict of interest

None.

## Funding

Funded by NIH NIAU01‐AG023712, U01‐AG23744, U01‐AG023746, U01‐AG023, U19 AG023122O, NHLBI (R21HL128871 and contract # N 01HC25195)

## Supporting information


**Fig. S1** Flow chart of the analytic approach.
**Fig. S2** Distribution of age at enrollment in LLFS.
**Fig. S3** Overview of cluster analysis to discover biomarker signatures.
**Fig. S4** (a,b) Age and sex distribution of biomarkers.
**Fig. S5–S17** Description of 26 biomarker signatures in LLFS
**Fig. S18** Age and sex specific distribution of 19 biomarkers in clusters 1–17.
**Fig. S19** Example of lab‐bias in the measurement of albumin.
**Fig. S20** distribution of externally standardized biomarkers in FHS data using LLFS means and standard deviations.
**Fig. S21** Distribution of biomarker signatures in LLFS and FHS offspring.
**Fig. S22** Reproduced biomarker signatures in FHS offspring.

**Table S1** baseline characteristics of LLFS participants included in the analysis by generation and sex (mean and standard deviation)
**Table S2** (Clusters 1 to 13) The table displays the cluster number, the cluster size, and the cluster signature defined by mean and standard deviation of the standardized biomarkers
**Table S2** (Clusters 14 to 26) The table displays the cluster number, the cluster size, and the cluster signature defined by mean and standard deviation of the standardized biomarkers
**Table S3** Results of the ‘Leave‐one‐biomarker‐out’ replication
**Table S4** Summary demographics of patients allocated to the 26 clusters
**Table S5** Comparative analysis of 7 aging related phenotypes in LLFS
**Table S6** The table list the biomarkers available in the FHS by generation (g1: original; g2: offspring; g3: grand‐children), exam number, and age range and number of patients with available data
**Table S7** The table show the list of biomarkers in which there is a significant lab effect (column 2) and a significant cohort effect (column 3)
**Table S8** Sensitivity and misclassification rate of the Bayes Rule
**Table S9** Positive predicted values rate of the Bayes Rule
**Table S10** PPV rate of the Bayes rule with the subset of biomarkers available in the FHS generation 1
**Table S11** PPV rate of the Bayes rule with subset of biomarkers in FHS offspring
**Table S12** Replication of association of signatures with mortality (FHS Cohort)
**Table S13** Replication of association of signatures with CVD risk (FHS Cohort)
**Table S14** Replication of association of signatures with Type 2 Diabetes (FHS Cohort)
**Appendix S1** Material and methods
