# Supplementary material for: Biomarker signatures of aging
Source: Aging Cell. 2017 Jan 6;16(2):329–38. doi: 10.1111/acel.12557 (PMC5334528; doi:10.1111/acel.12557)

Paola Sebastiani, Bharat Thyagarajan, Fangui Sun, Nicole Schupf, Anne B Newman, Monty Montano, Thomas T Perls

BIOMARKER SIGNATURES OF AGING: SUPPLEMENT FIGURES 1-22

Table of content

- Supplement Figure S1: Flow chart of the analytic approach
- Supplement Figure S2: Distribution of age at enrollment in LLFS
- Supplement Figure S3: Overview of cluster analysis to discover biomarker signatures
- Supplement figure S4(a-b): age and sex distributions of biomarkers
- Supplement Figures S5 through S17: description of 26 biomarker signatures in LLFS
- Supplement Figure S18a through S18p: Age and sex specific distribution of 19 biomarkers in clusters 1-17. Red and blue denote females and males in cluster 1; magenta and cyan denote females and males in the cluster described in the title page
- Supplement Figure S19: example of lab-bias in the measurement of albumin
- Supplement Figure S20a through S20w: distribution of externally standardized biomarkers in FHS data using LLFS means and standard deviations
- Supplement Figure S21: Distribution of biomarker signatures in LLFS and FHS offspring
- Supplement Figure S22a through S22g: Reproduced biomarker signatures in FHS offspring

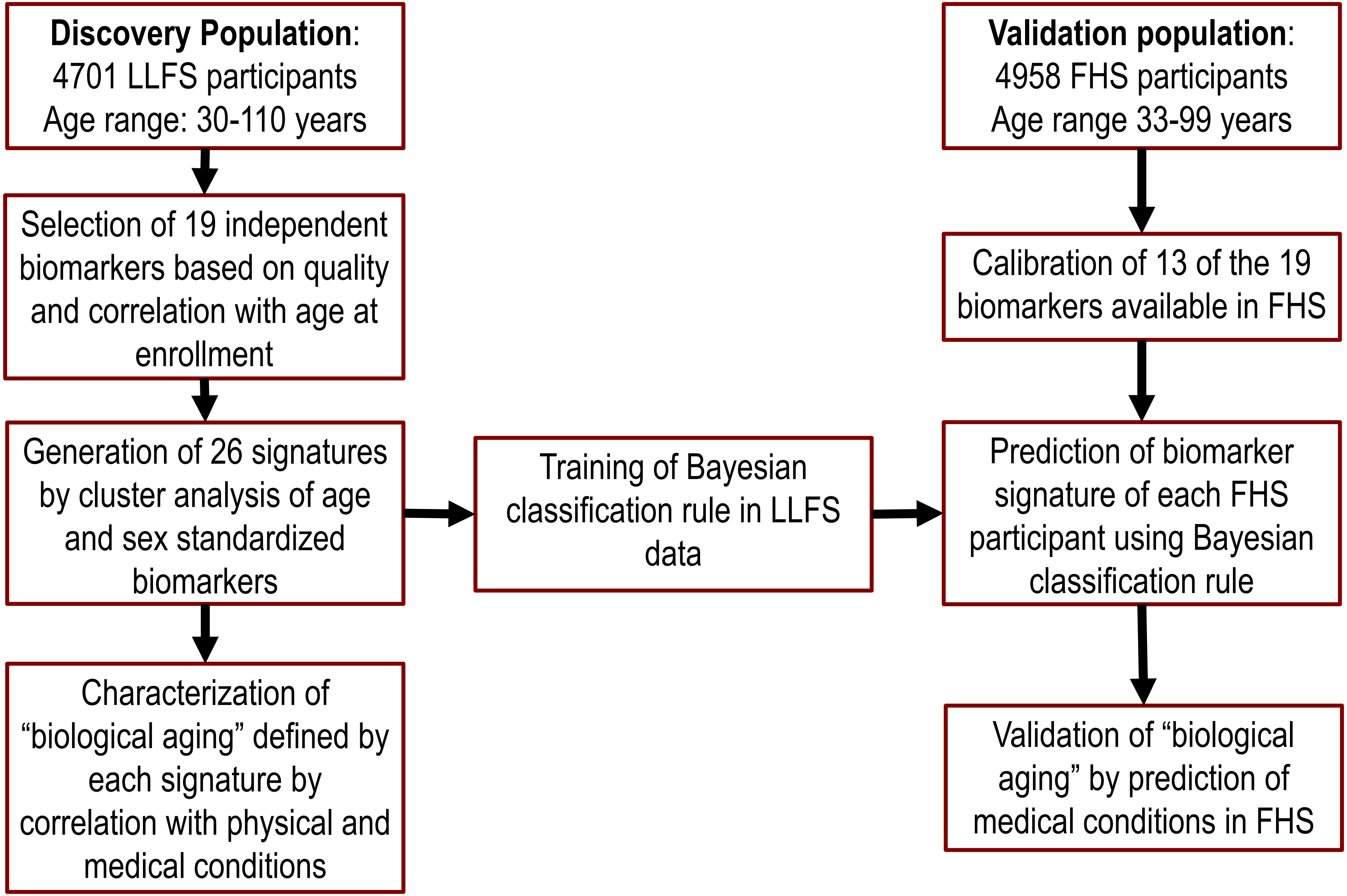

Supplement Figure S1: Flow chart of the analytic approach

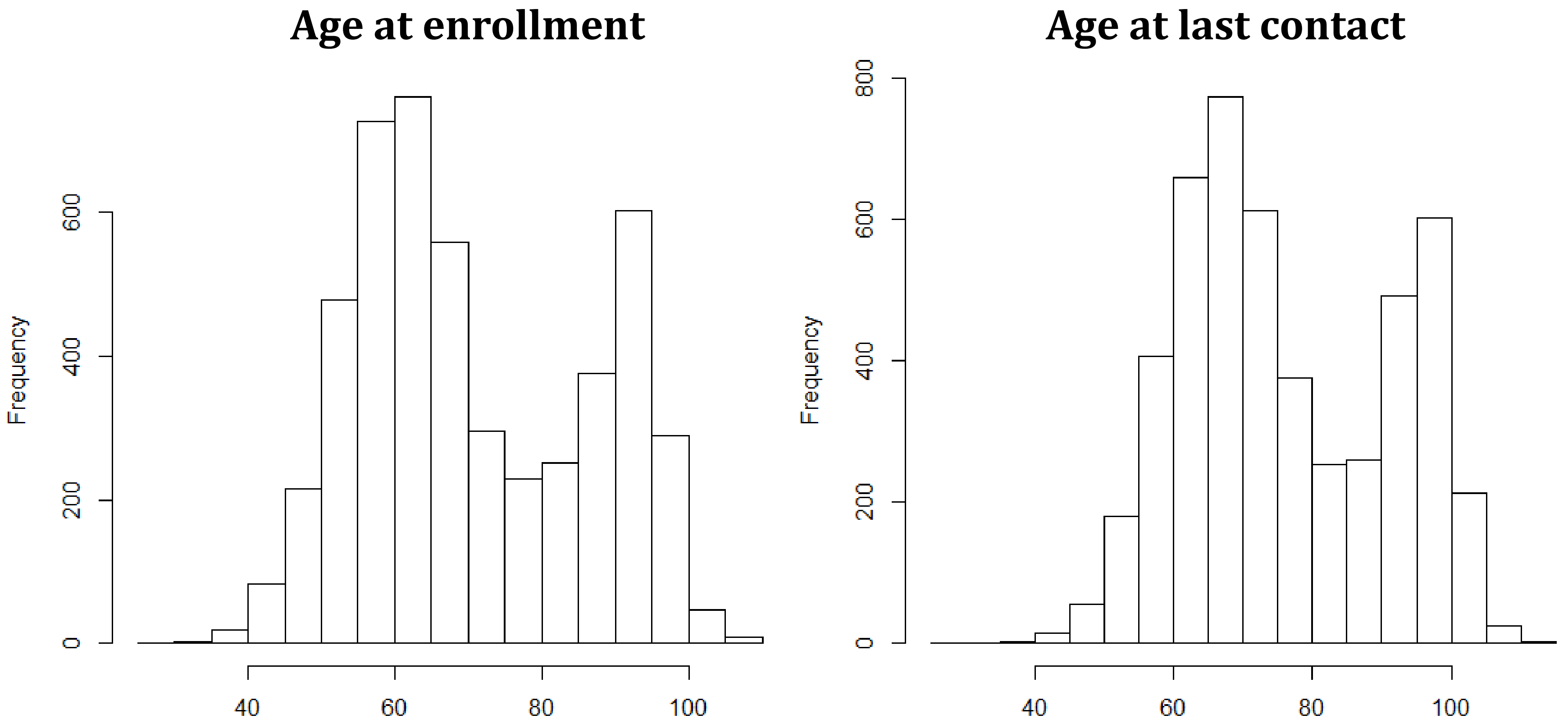

Supplement Figure S2: Distribution of age at enrollment and age at last contact in the 4701 LLFS participants included in the analysis

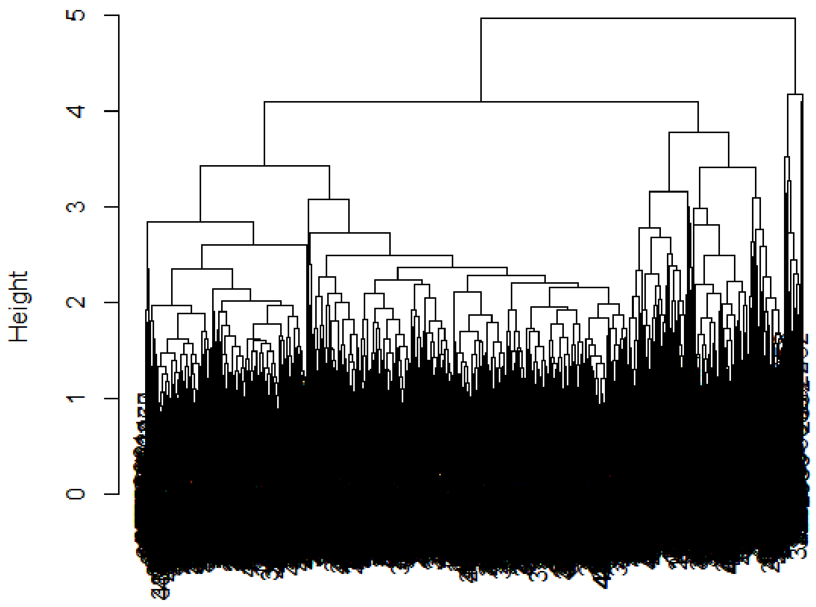

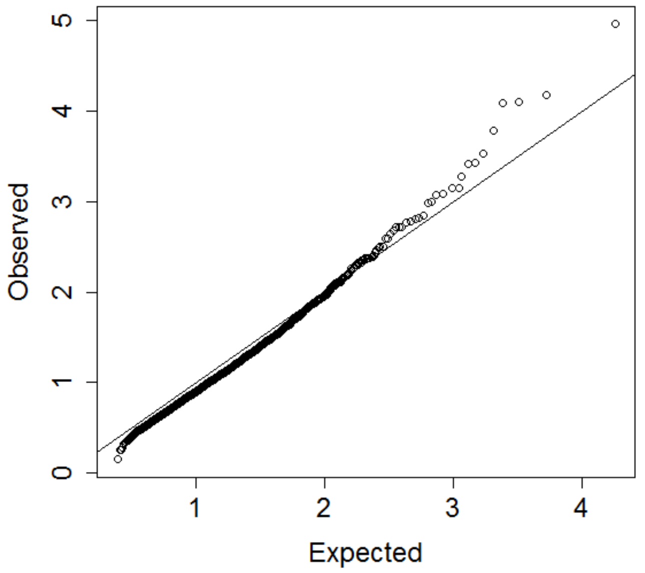

α	Cluster Size for Different Levels of Significance																																N clusters
**0.001**	2298				1160			401					462								251				102			18			3	5	4	10
**0.002**	2293			5	1160			401					414					48			211		40		102			12		6	3	5	4	14
**0.003**	2293			5	1128		32	387		11		3	414					28	14	6	211		40		102			12		6	3	5	4	19
**0.004**	2262		31	5	1128		32	387		11		3	178		140		96	28	14	6	178	33	29	11	91		11	8	4	6	3	5	4	26
**0.005**	2262		31	5	1120	8	32	387		11		3	159	19	140		96	28	14	6	178	33	29	11	91		11	8	4	6	3	5	4	28
**0.006**	1919	343	31	5	1120	8	32	195	192	3	8	3	159	19	131	9	96	28	14	6	178	33	29	11	61	30	11	8	4	6	3	5	4	33

Supplement Figure S3: *Top left* – dendrogram displaying the arrangement of subject profiles by hierarchical clustering with complete linkage. The distances are normalized by the number of biomarkers (n=19). *Top right*: QQ-plot displaying the observed and expected distances used in hierarchical clustering (height of branch nodes). Departure from the diagonal line suggests that there are significant clusters in the data. *Bottom*: Cluster composition for different levels of significance a (first column). Colors track clusters that are robust with respect to different levels of significant. The number of clusters ranges from 10 for a=0.1% to 33 a=0.6% but most of the differences are in the generation of new clusters with very small number of individuals. With a= 0.4% the algorithm detects 26 clusters, and the most noticeable difference from the clusters detected with a between 0.1% and 0.3% is the split of the cluster with 462 participants into 3 smaller clusters of 96, 140 and 178 participants (Clusters highlighted in blue). For all subsequent analyses, we used the 26 clusters detected for a significance level of 0.4% that provides a good compromise between number of clusters and error rate.

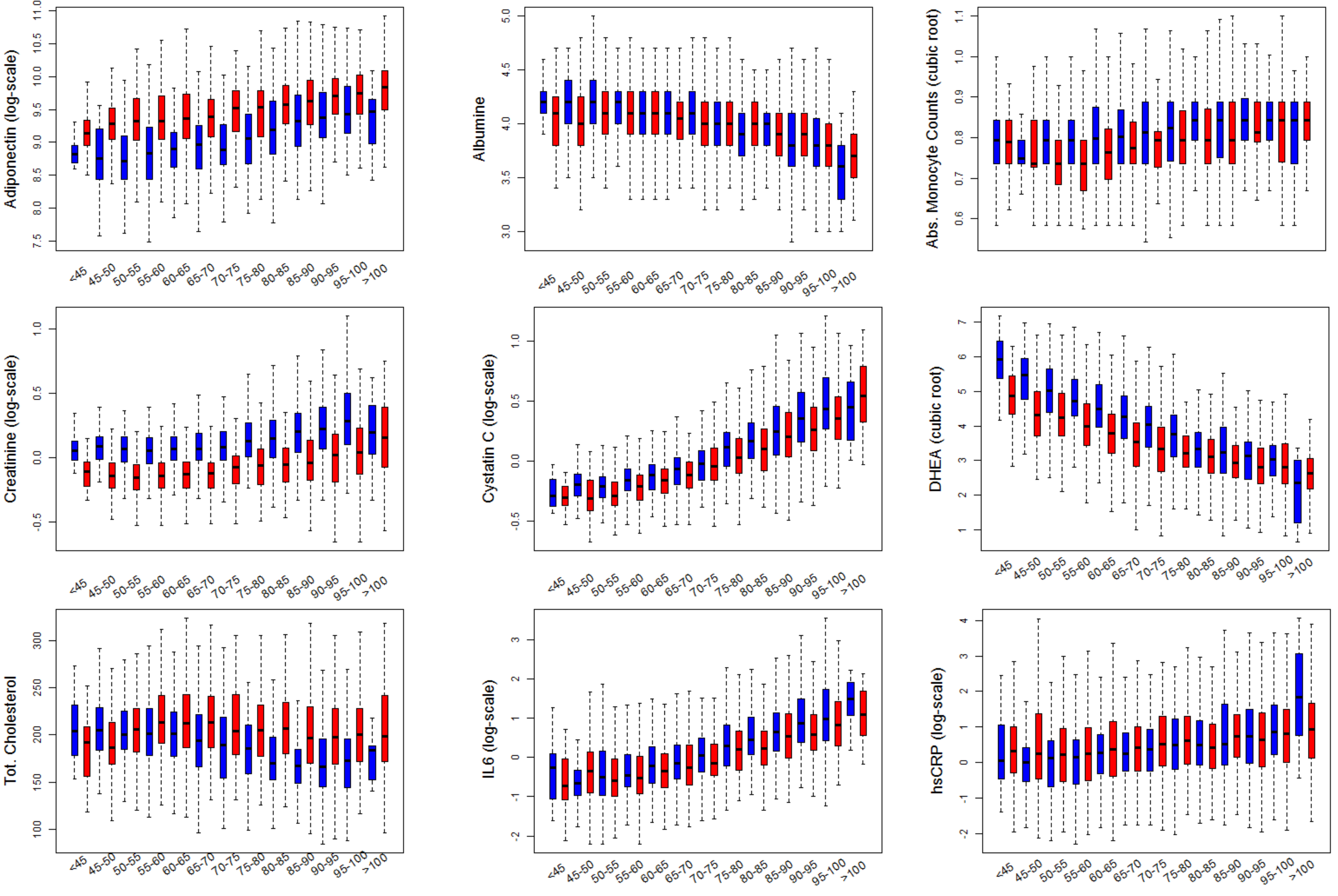

Supplement Figure S4a: Age and sex distribution of selected biomarkers (blue=males, red=females.)

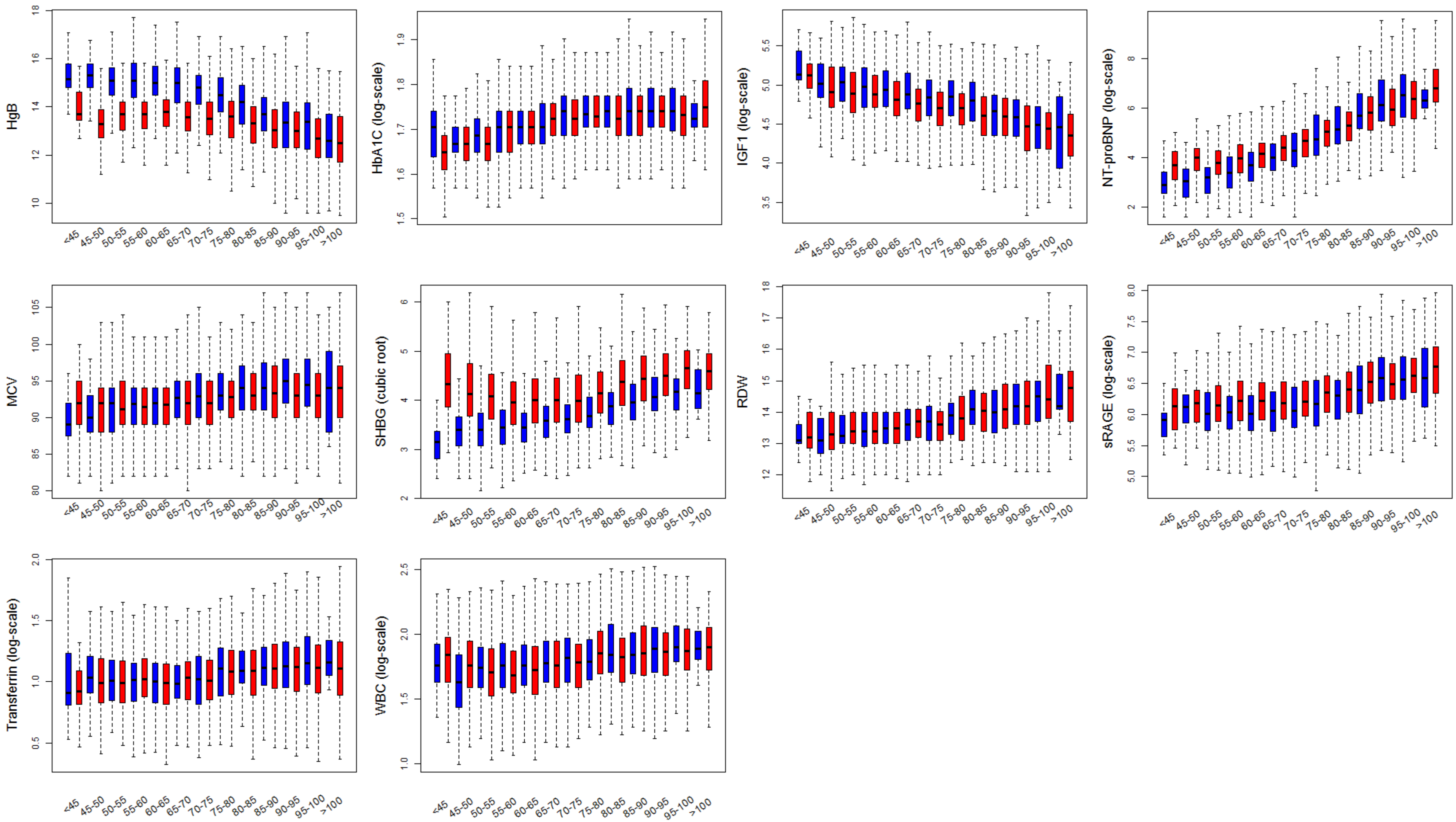

Supplement Figure S4b: Age and sex distribution of selected biomarkers (blue=male, red=females.). Mean and standard deviation per age group were used to define the age and sex specific z-score. Outliers were removed using 2.5% trimmed means.

Supplement Figure S5: Profiles 1 and 2

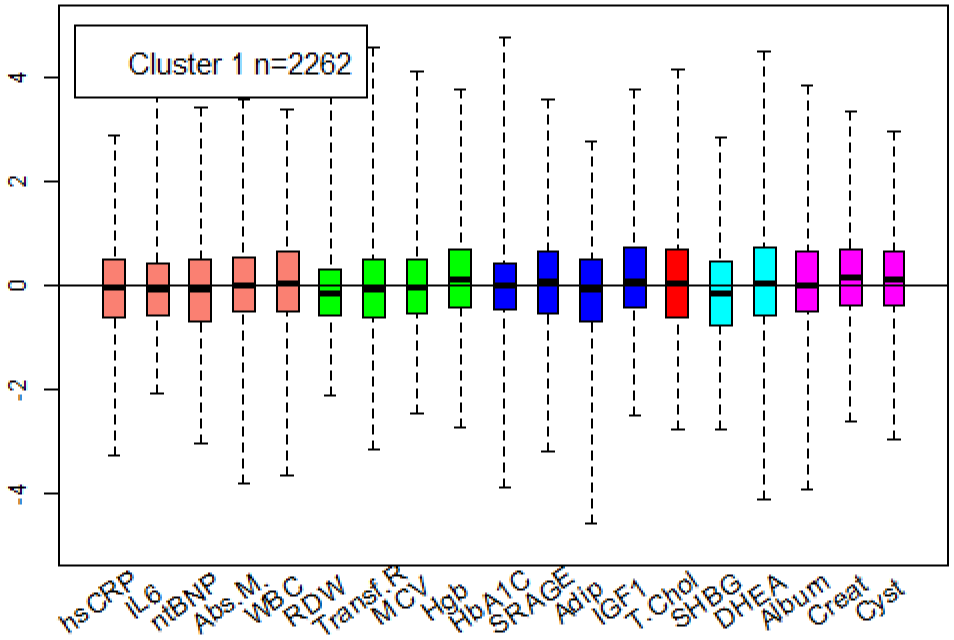

"**Referent profile**" : biomarkers are equal to the value expected for age/sex

Cluster:
37% born < 1935; median age 90
63% born ≥ 1935; median age 60
54% females

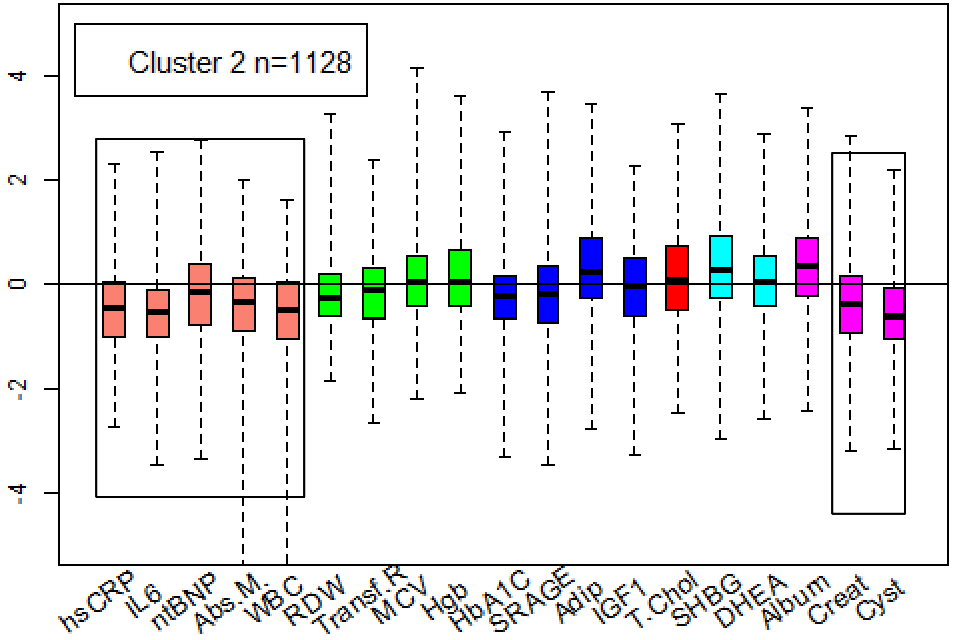

Profile 2: a group of biomarkers lower than the value expected for age/sex

Cluster:
41% born < 1935; median age 90
59% born ≥ 1935; median age 61
55% females

Supplement Figure S6: Profiles 3 and 4

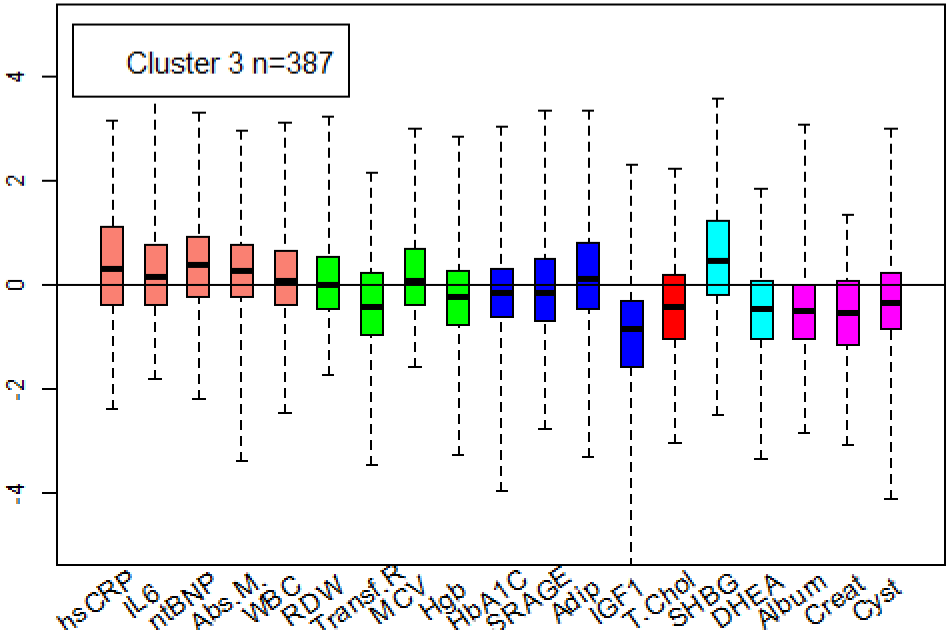

Profile 3: higher than average inflammation, low DHEA and albumin suggesting frailty

Cluster:
34% born < 1935; median age 91
66% born $\geq$ 1935; median age 60
59% females

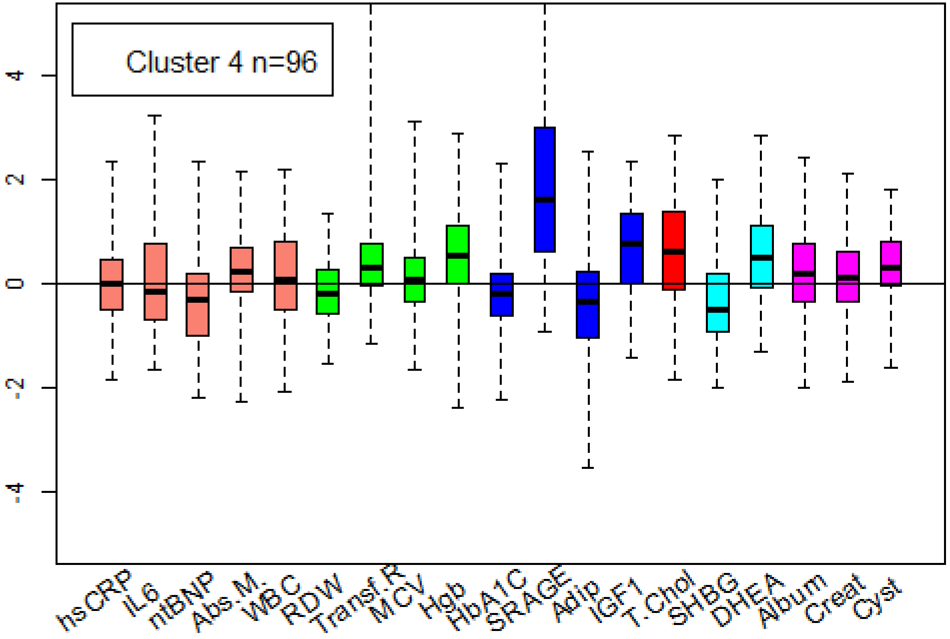

Profile 4: extremely elevated sRAGE, IGF1 and cholesterol

Cluster:
36% born < 1935; median age 92
64% born $\geq$ 1935; median age 61
55% females

Supplement Figure S7: Profiles 5 and 6

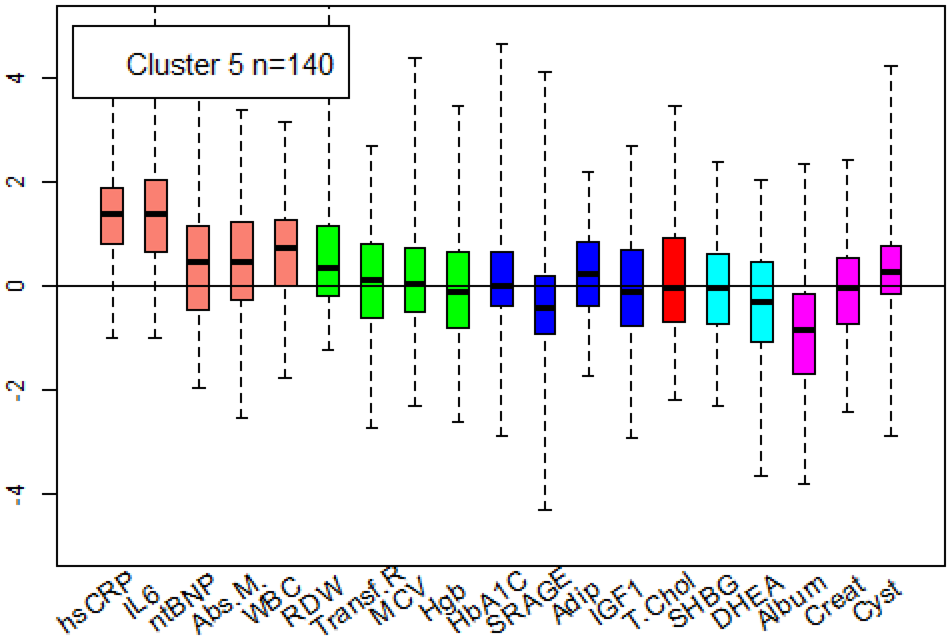

Profile 5:

Cluster:

41% born < 1935; median age 91

59% born ≥ 1935; median age 59

46% females

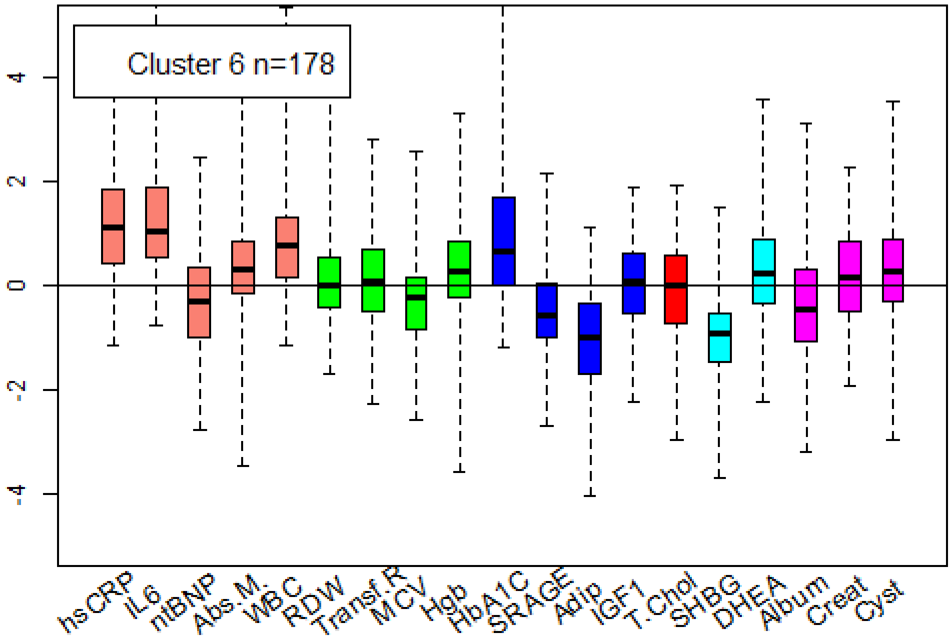

Profile 6:

Cluster:

36% born < 1935; median age 90

64% born ≥ 1935; median age 62

62% females

Supplement Figure S8: Profiles 7 and 8

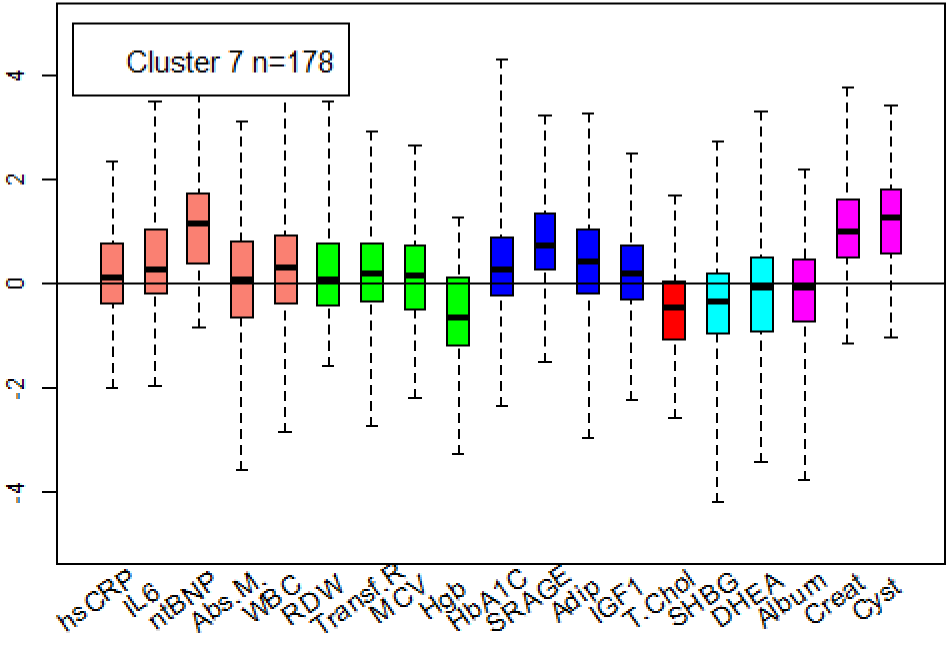

Profile 7:

Cluster:

56% born < 1935; median age 92

44% born ≥ 1935; median age 62

55% females

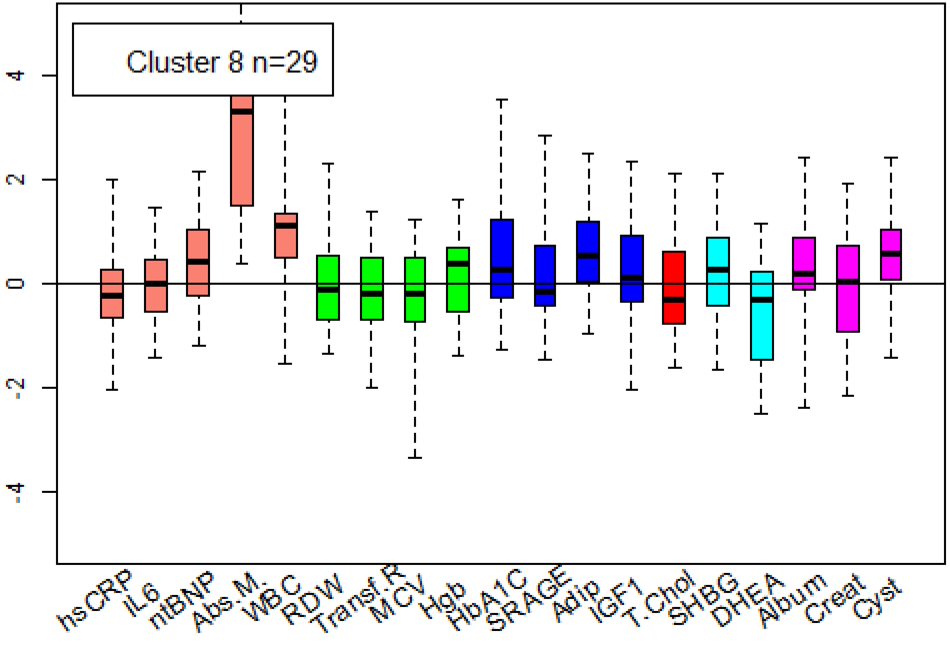

Profile 8:

Cluster:

55% born < 1935; median age 91

45% born ≥ 1935; median age 58

48% females

Supplement Figure S9: Profiles 9 and 10

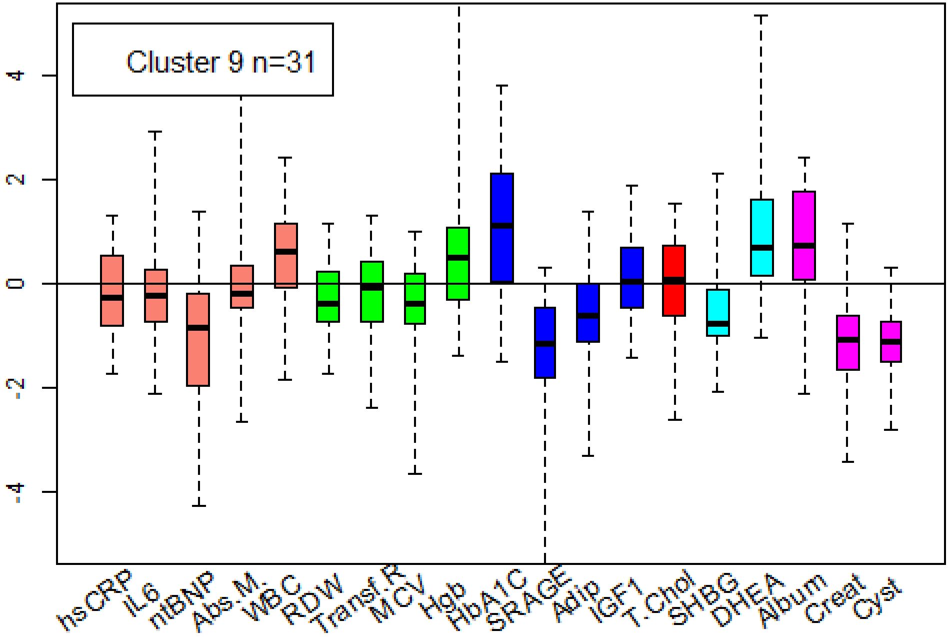

Profile 9:

Cluster:

42% born < 1935; median age 87

58% born ≥ 1935; median age 64

48% females

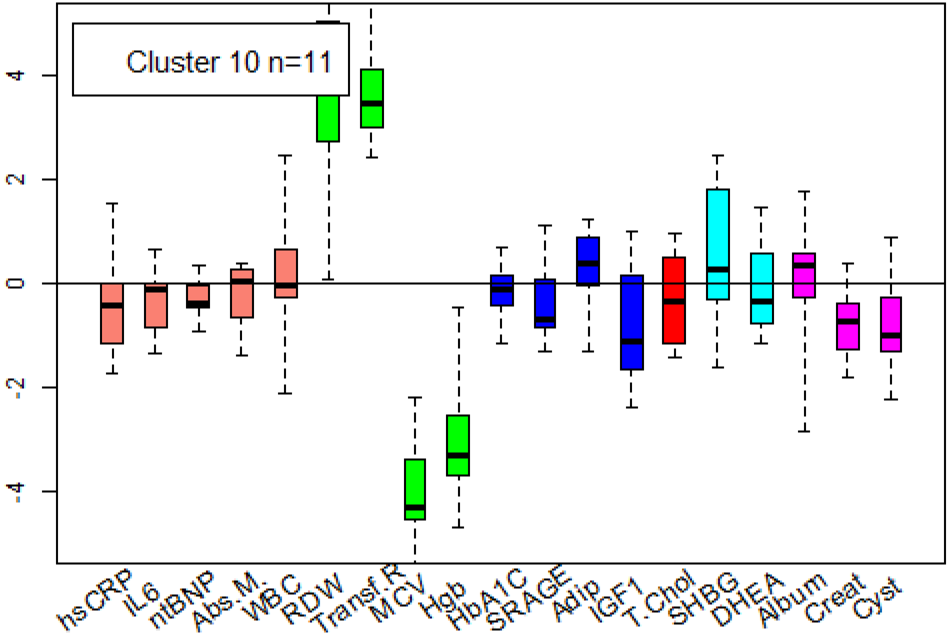

Profile 10:

Cluster:

45% born < 1935; median age 88

55% born ≥ 1935; median age 52

73% females

Supplement Figure S10: Profiles 11 and 12

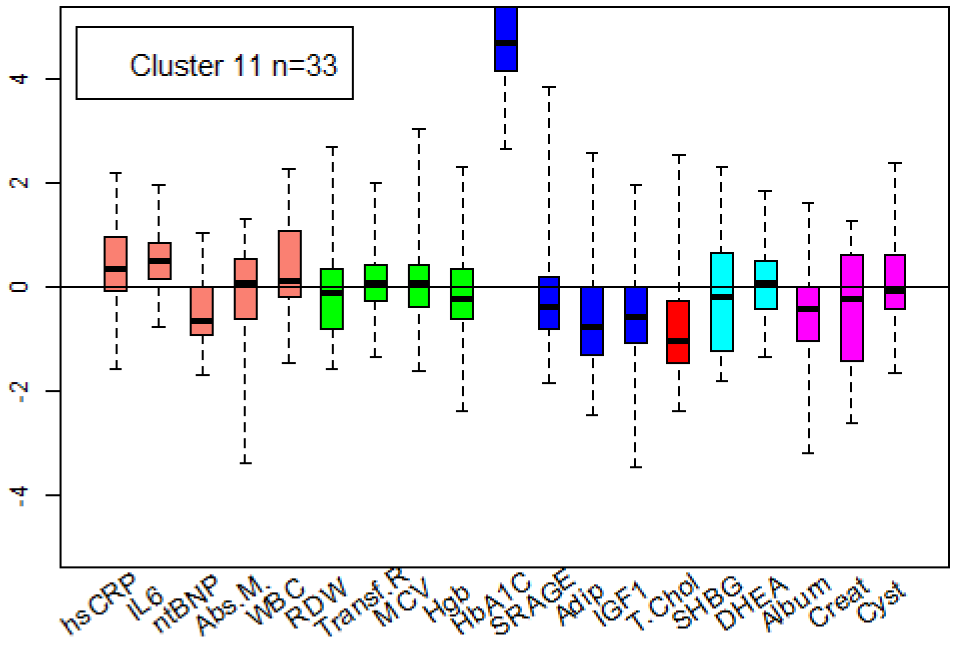

Profile 11:
Cluster:
27% born < 1935; median age 85
73% born ≥ 1935; median age 59
49% females

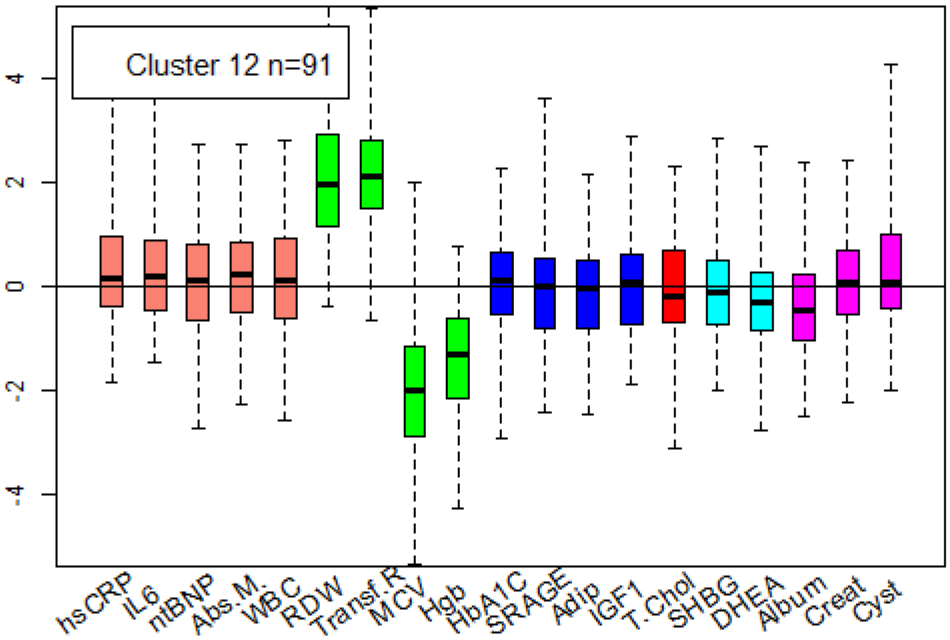

Profile 12:
Cluster:
42% born < 1935; median age 92
58% born ≥ 1935; median age 59
54% females

Supplement Figure S11: Profiles 13 and 14

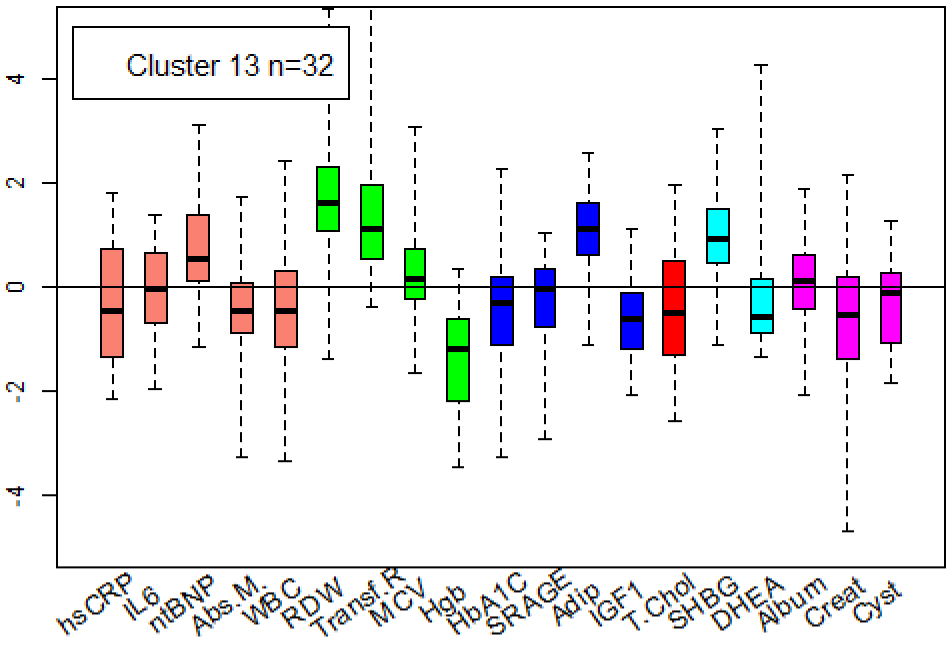

Profile 13:

Cluster:

66% born < 1935; median age 89

34% born ≥ 1935; median age 57

55% females

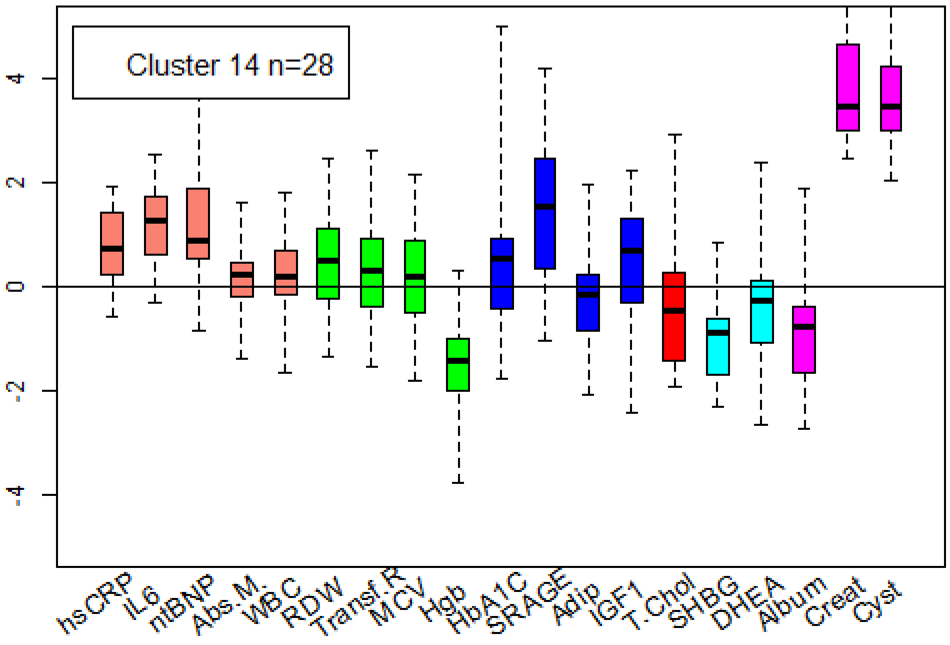

Profile 14:

Cluster:

57% born < 1935; median age 91

43% born ≥ 1935; median age 57

61% females

Supplement Figure S12: Profiles 15 and 16

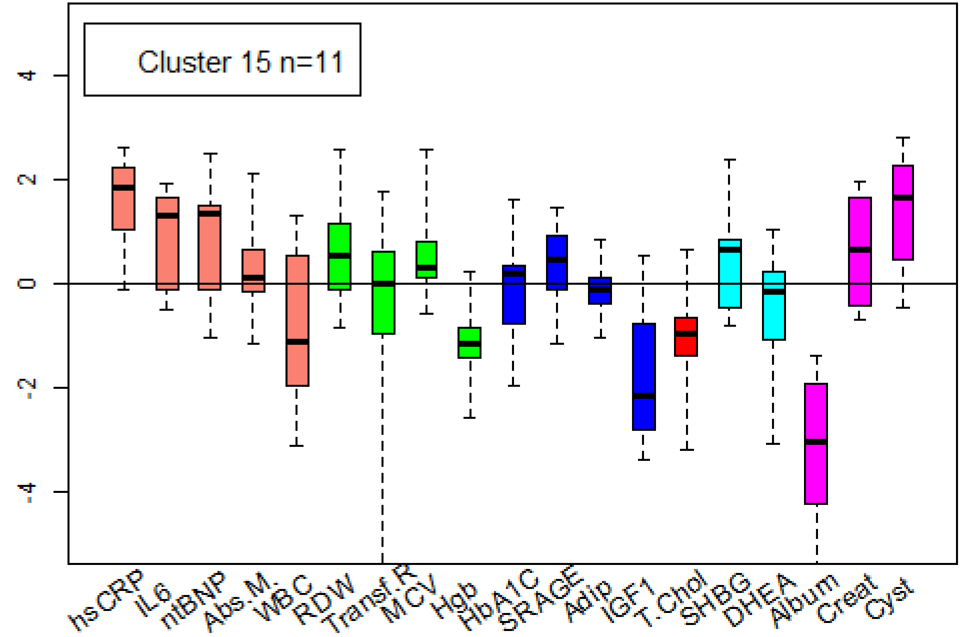

Profile 15:

Cluster:

55% born < 1935; median age 89

45% born > 1935; median age 63

73% females

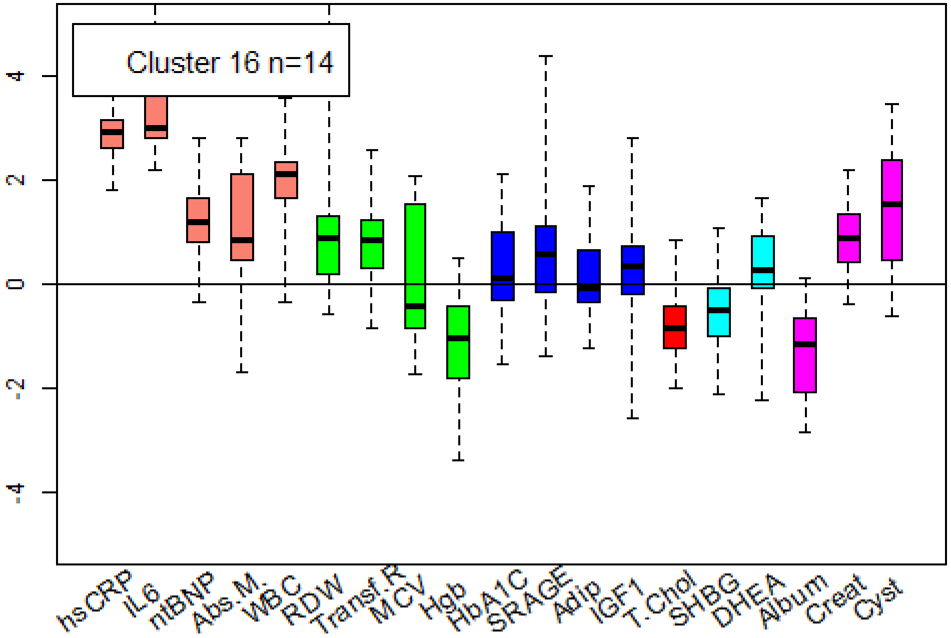

Profile 16:

Cluster:

64% born < 1935; median age 92

36% born > 1935; median age 63

29% females

Supplement Figure S13: Profiles 17 and 18

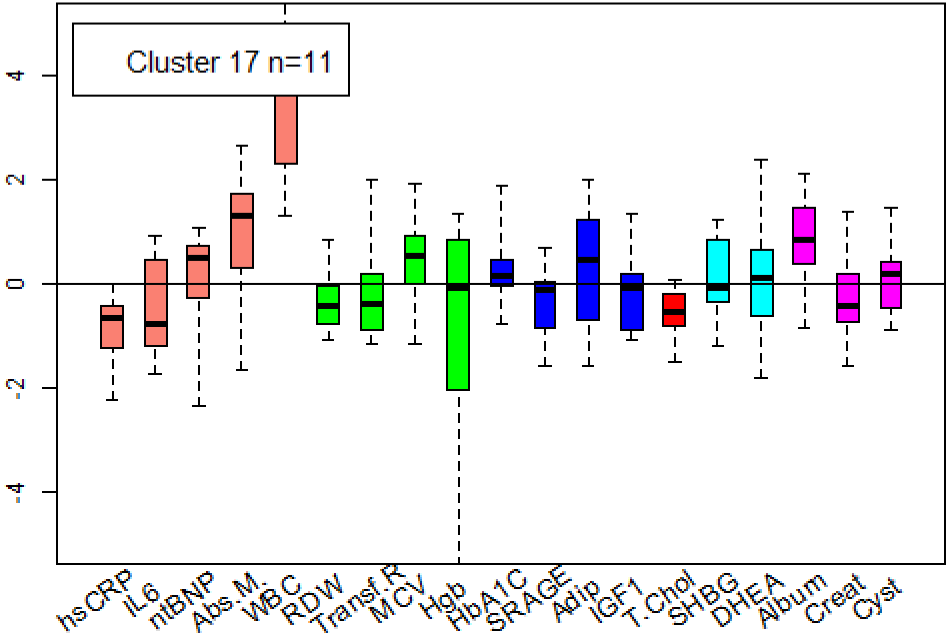

Profile 17:

Cluster:

55% born < 1935; median age 90

45% born ≥ 1935; median age 61

46% females

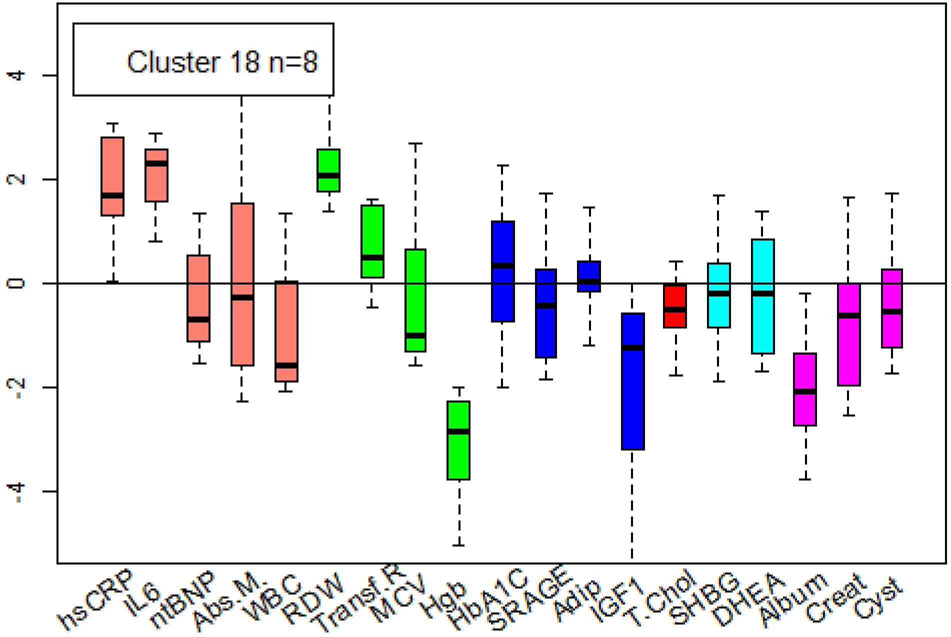

Profile 18:

Cluster:

50% born < 1935; median age 98

40% born ≥ 1935; median age 55

75% females

Supplement Figure S14: Profiles 19 and 20

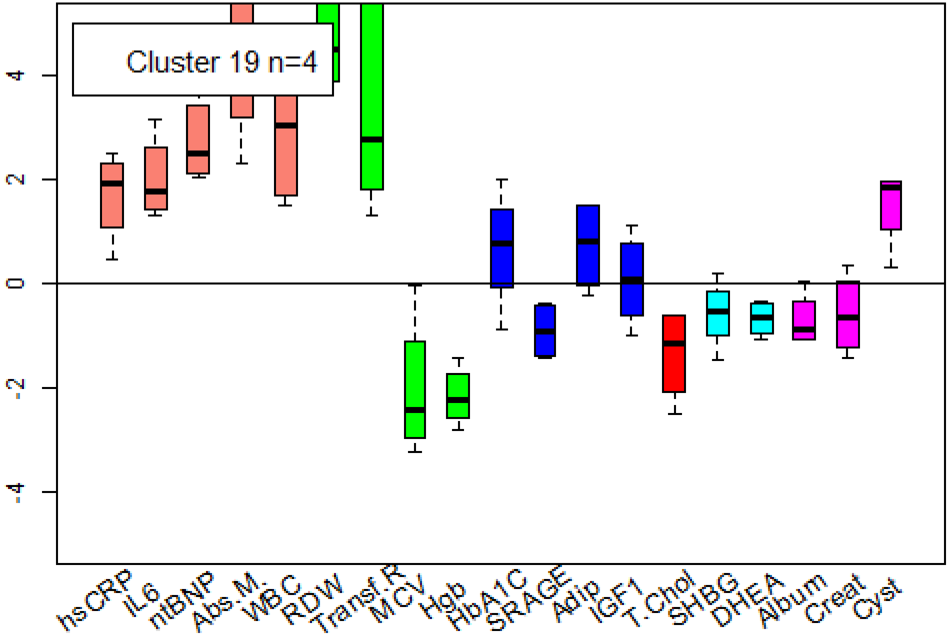

Profile 19:

Cluster:

50% born < 1935; median age 79

60% born > 1935; median age 59

25% females

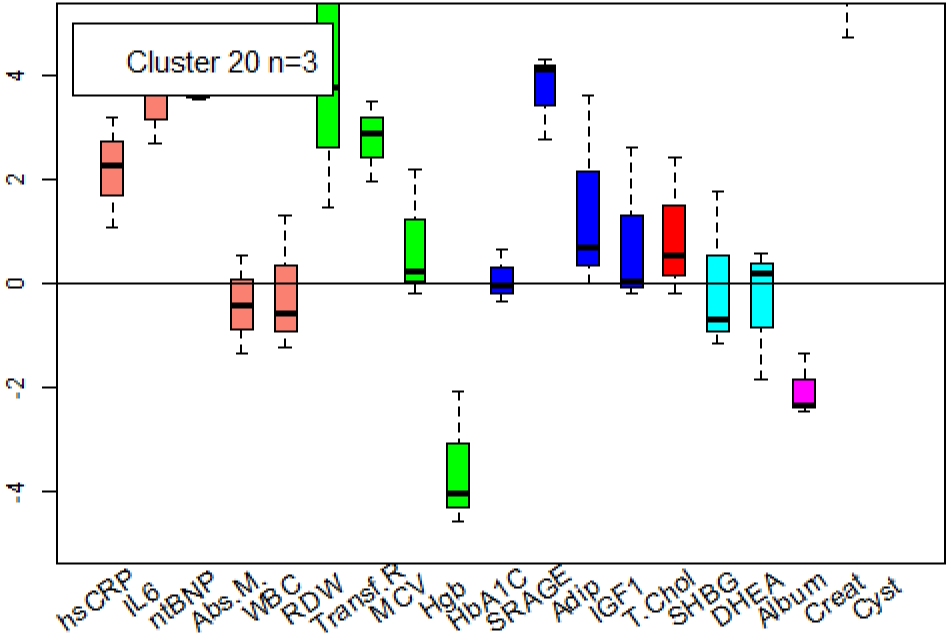

Profile 20:

Cluster:

0% born < 1935

100% born > 1935; median age 57

0% females

Supplement Figure S15: Profiles 21 and 22

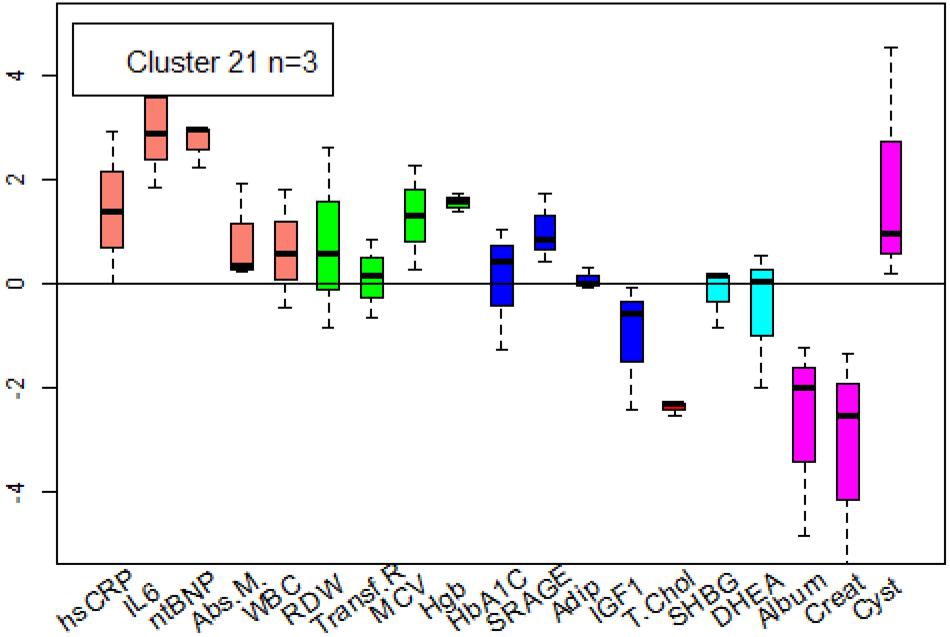

Profile 21:
Cluster:
0% born < 1935;
100% born ≥ 1935; median age 62
67% females

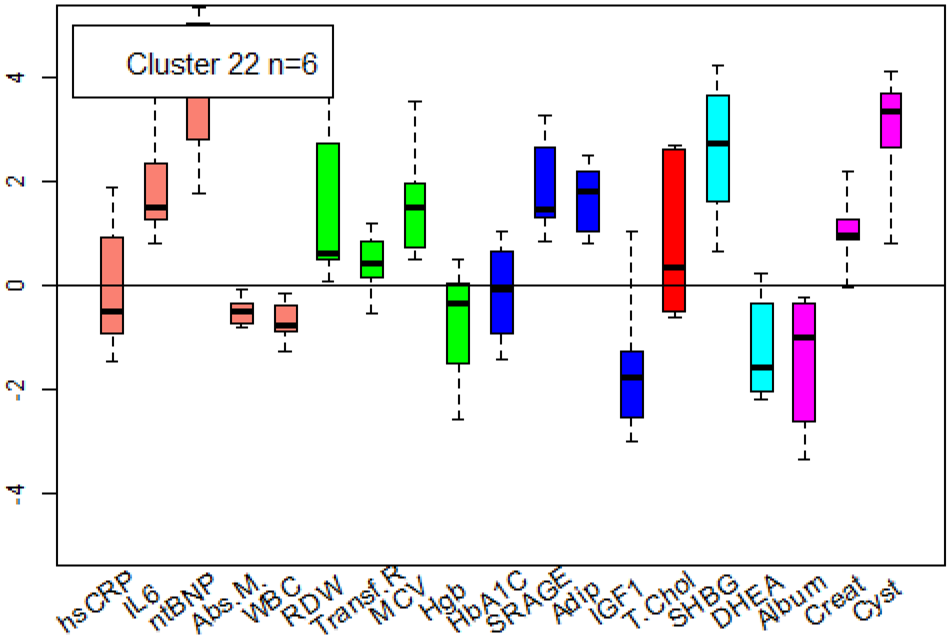

Profile 22:
Cluster:
0% born < 1935;
10% born ≥ 1935; median age 62
50% females

Supplement Figure S16: Profiles 23 and 24

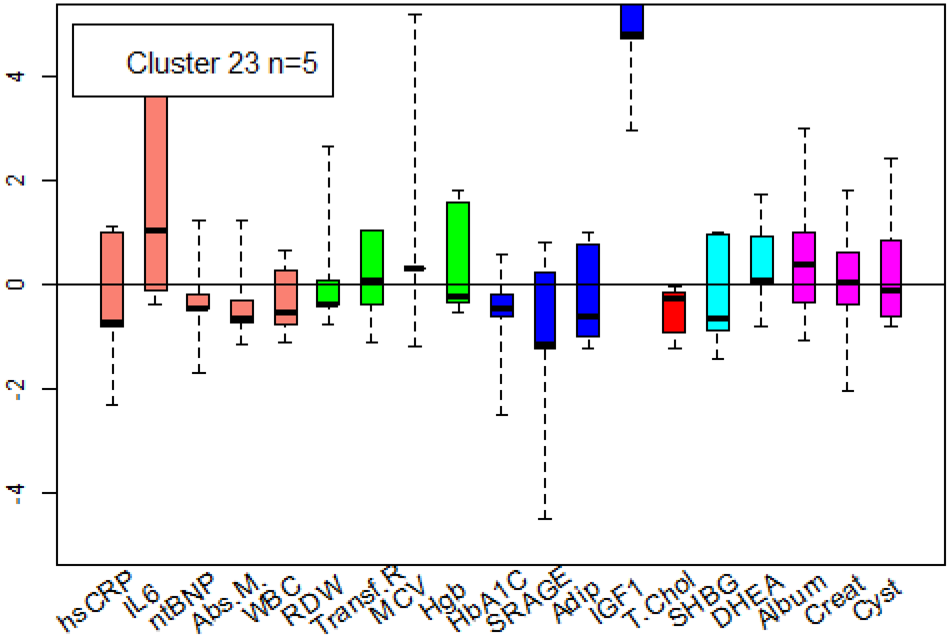

Profile 23:
Cluster:
60% born < 1935; median age 90
40% born ≥ 1935; median age 62
80% females

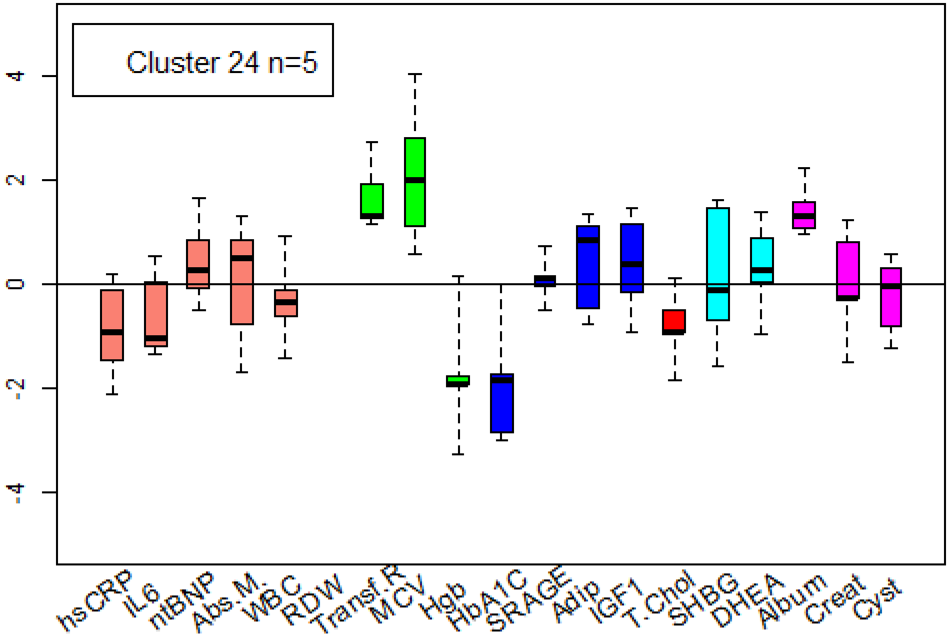

Profile 24:
Cluster:
80% born < 1935; median age 92
20% born ≥ 1935; median age 60
80% females

Supplement Figure S17: Profiles 25 and 26

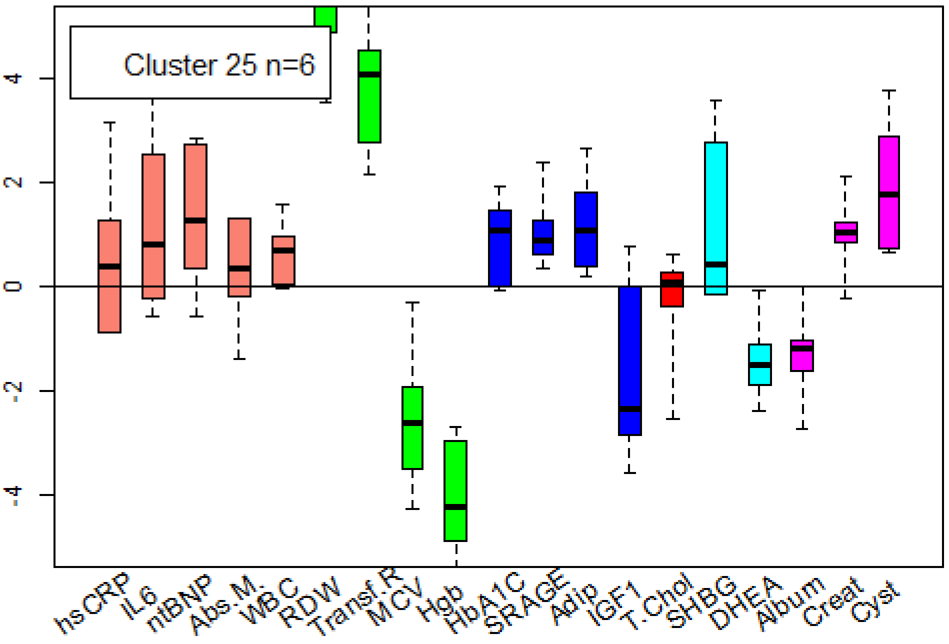

Profile 25:

Cluster:

16% born < 1935; median age 84

84% born ≥ 1935; median age 64

50% females

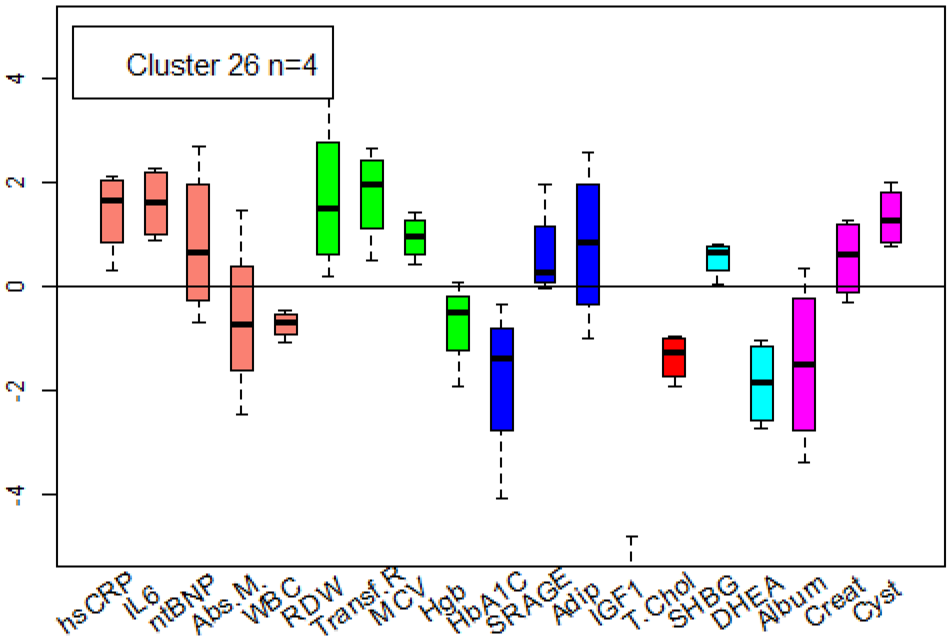

Profile 26:

Cluster:

50% born < 1935; median age 97

50% born ≥ 1935; median age 57

50% females

Supplement Figure S18_a: Referent Cluster

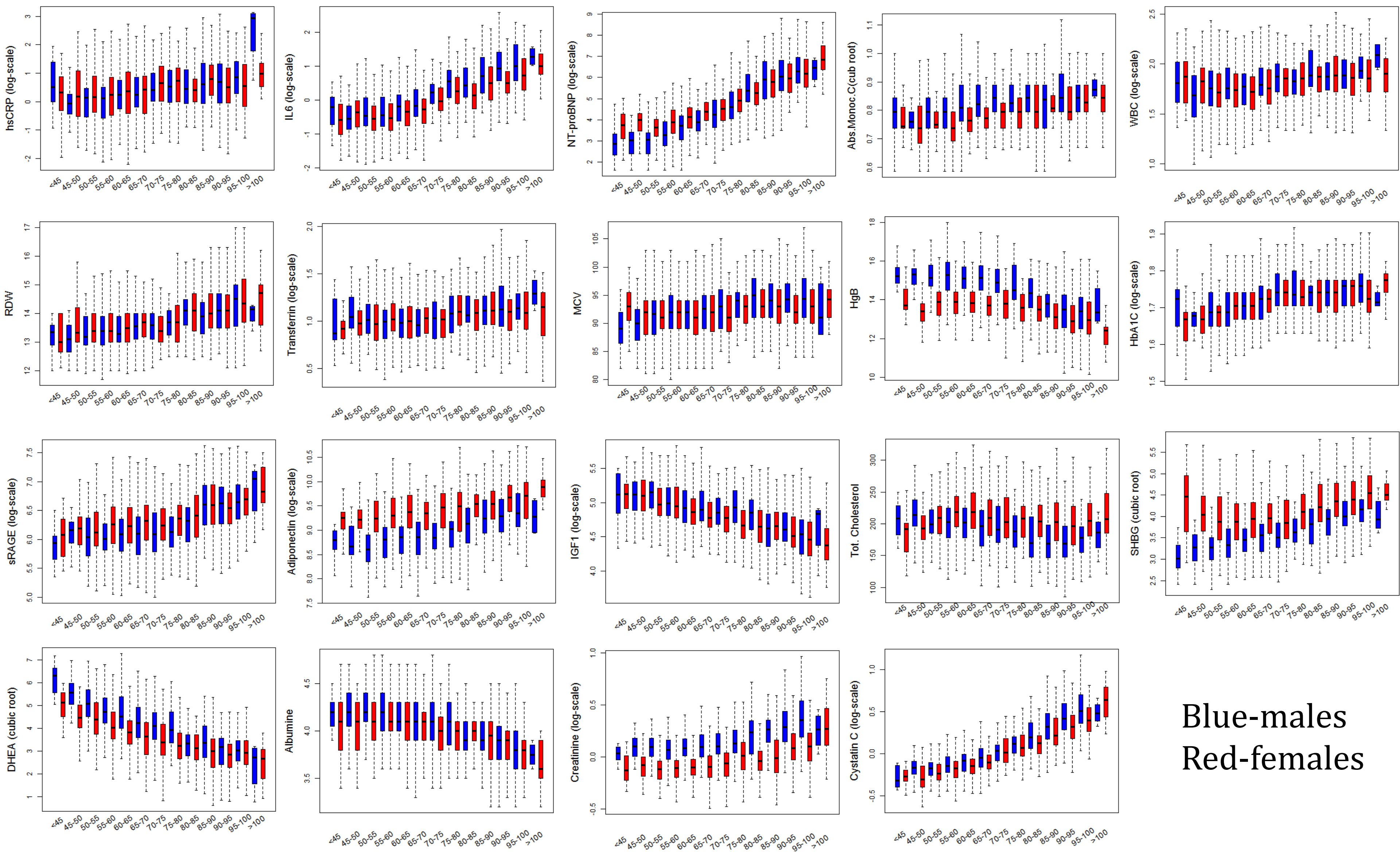

Supplement Figure S18_b: Cluster 2

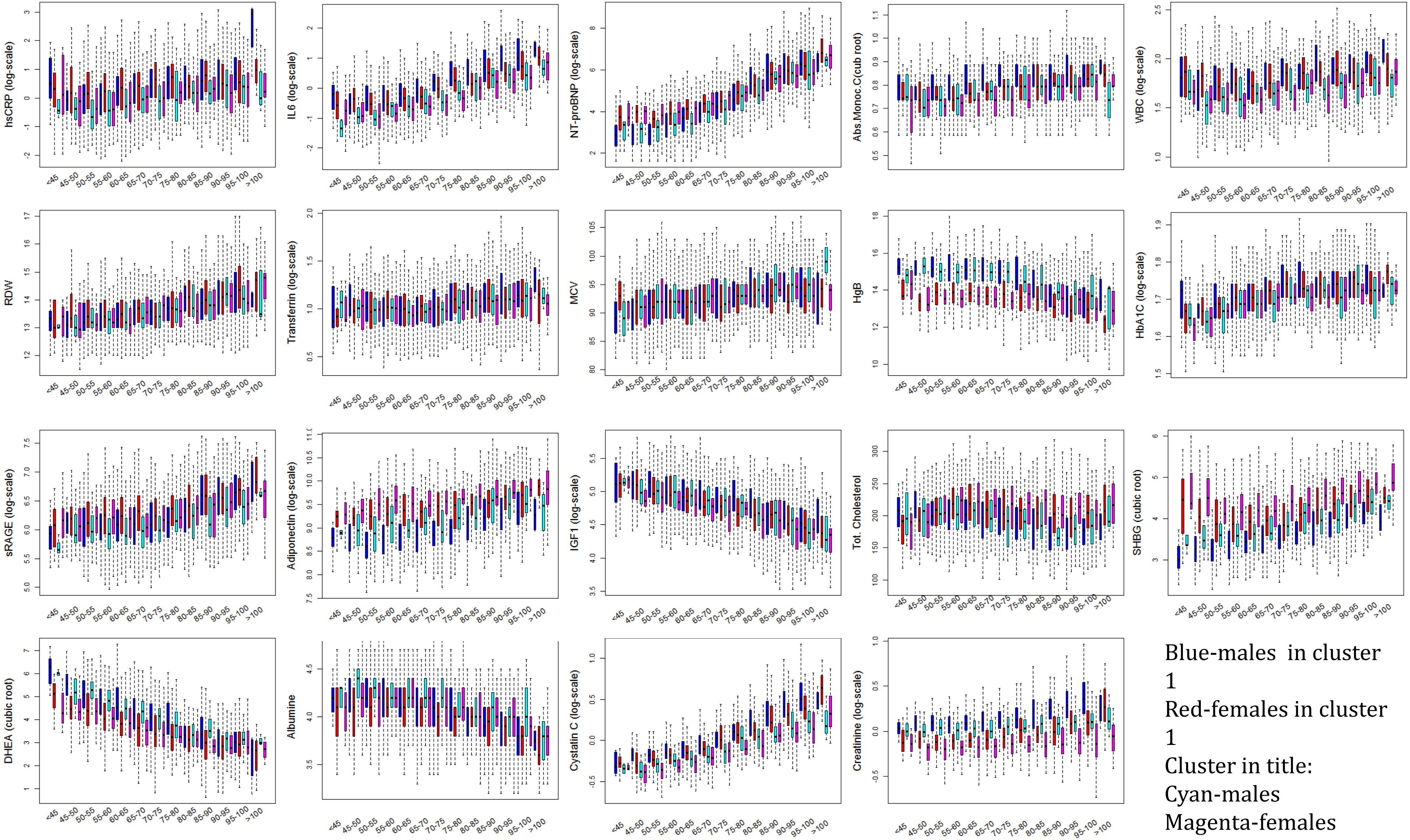

Blue-males in cluster 1
Red-females in cluster 1
Cluster in title:
Cyan-males
Magenta-females

Supplement Figure S18_c: Cluster 3

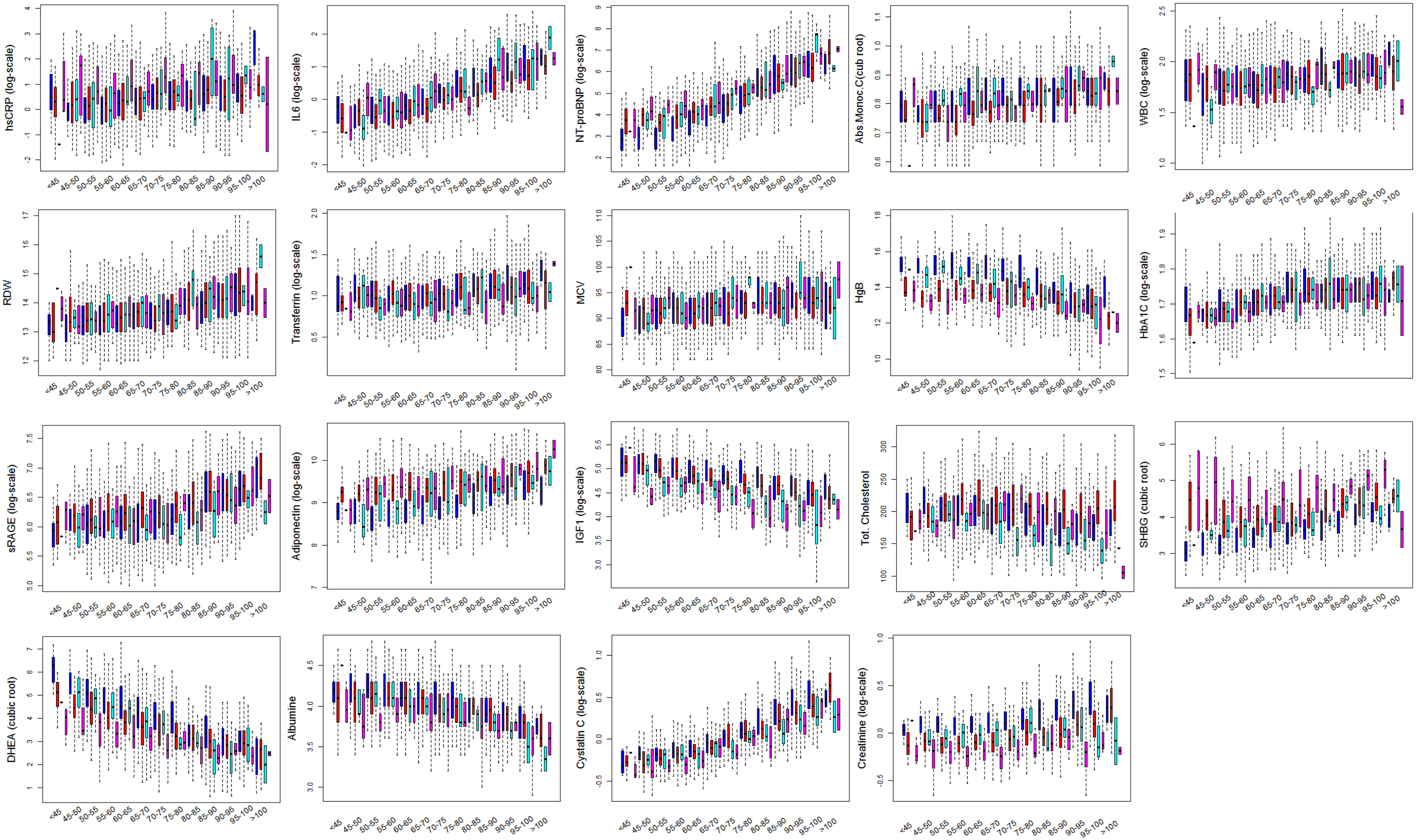

Supplement Figure S18_d: Cluster 4

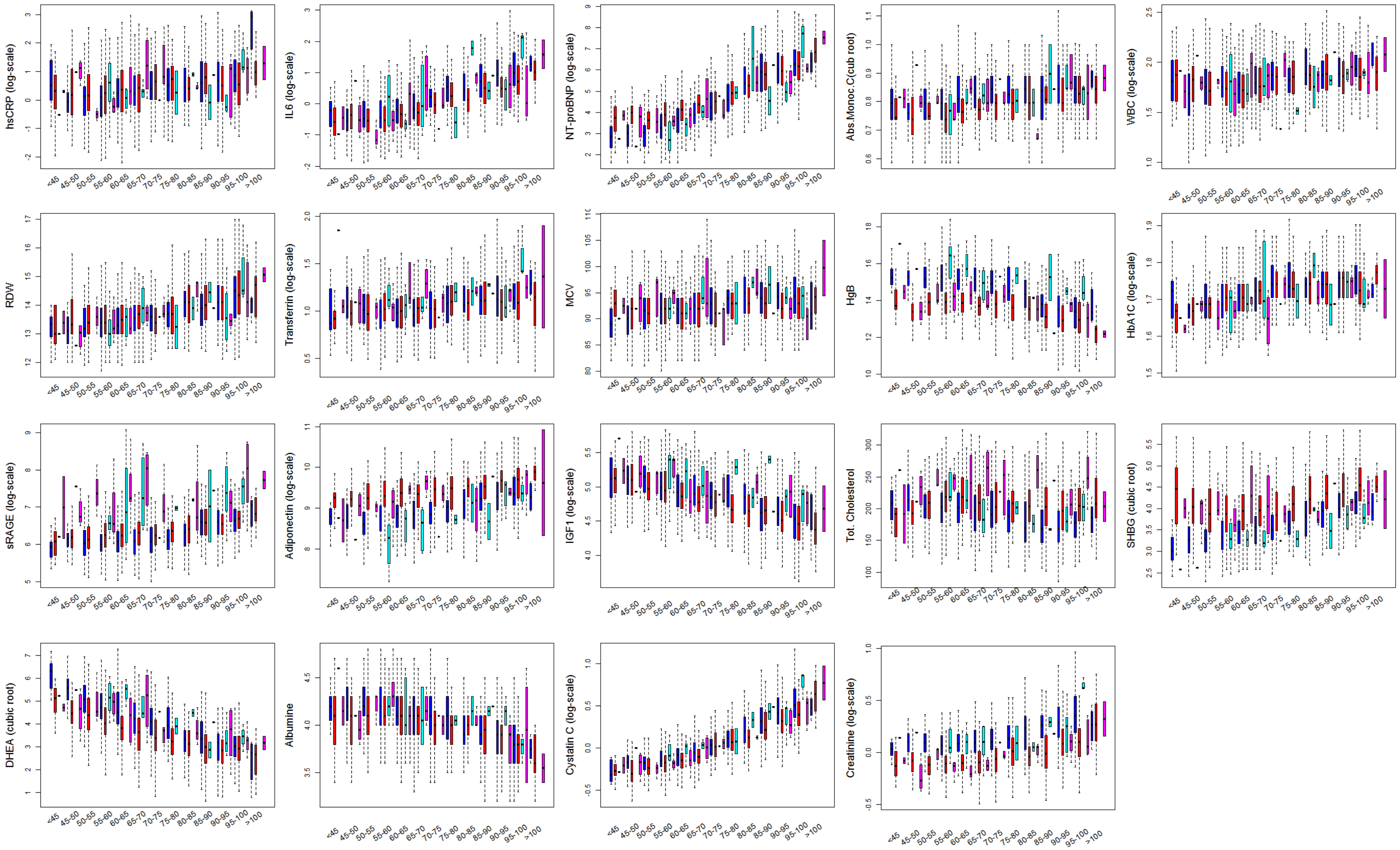

Supplement Figure S18_e: Cluster 5

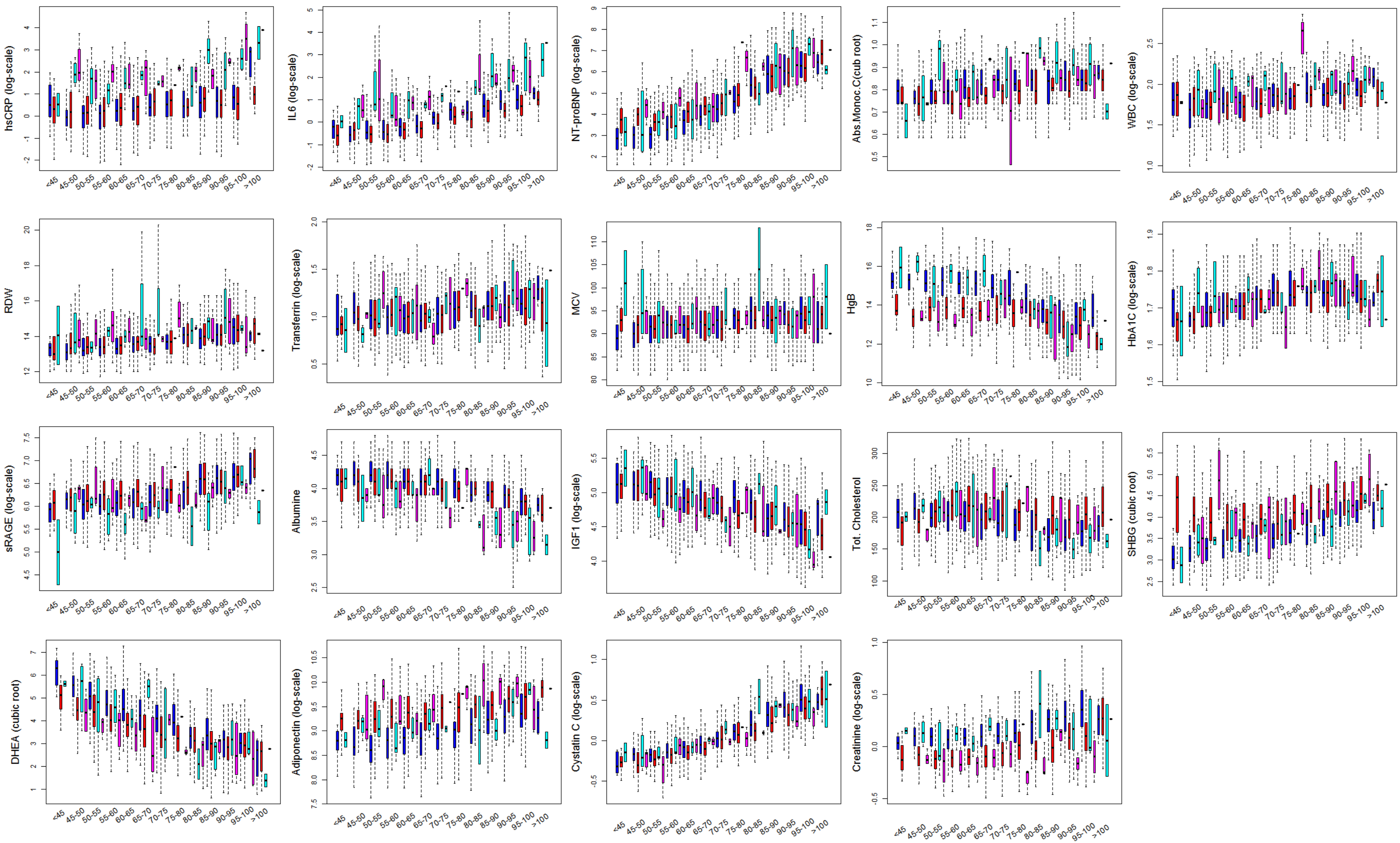

Supplement Figure S18_f: Cluster 6

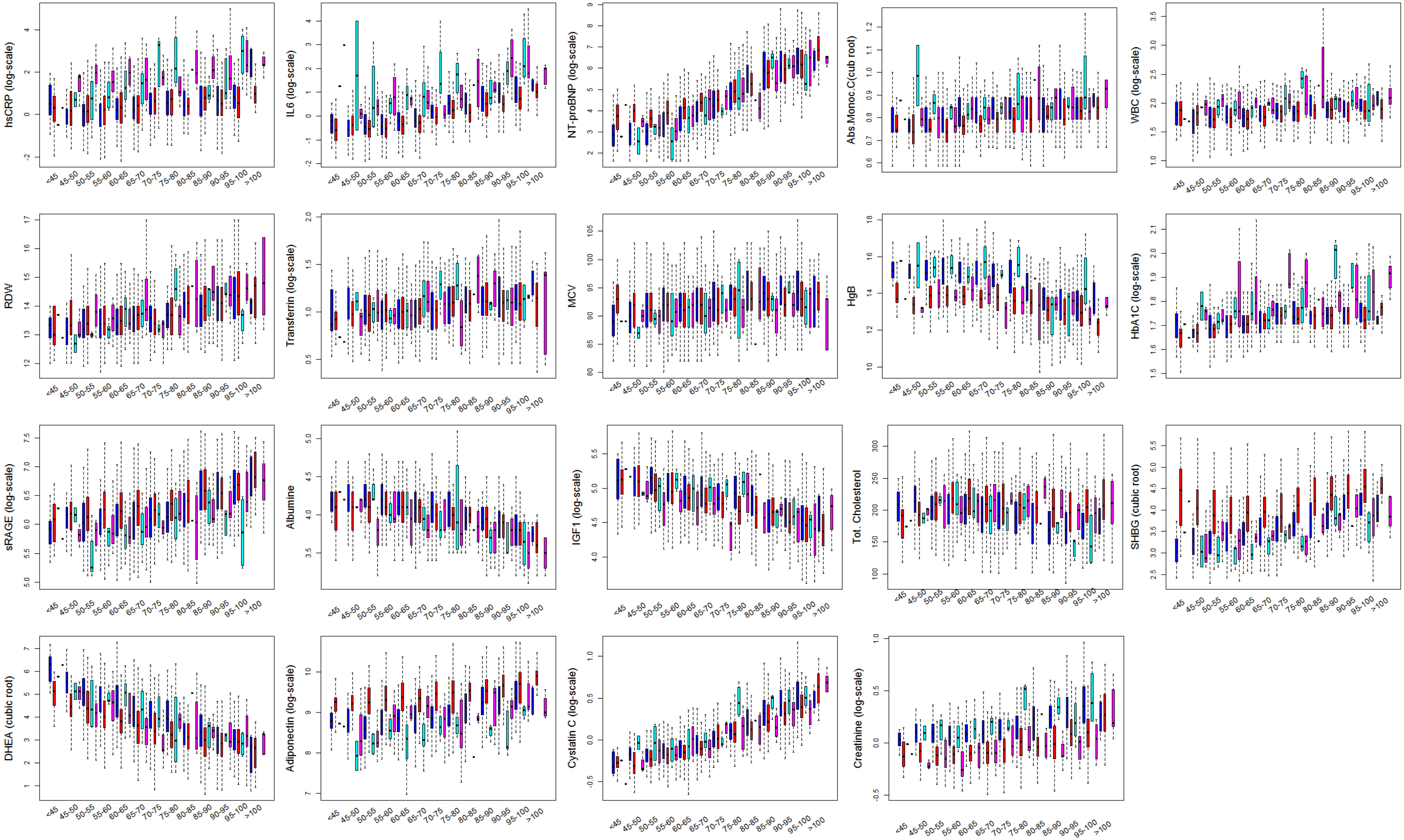

Supplement Figure S18_g: Cluster 7

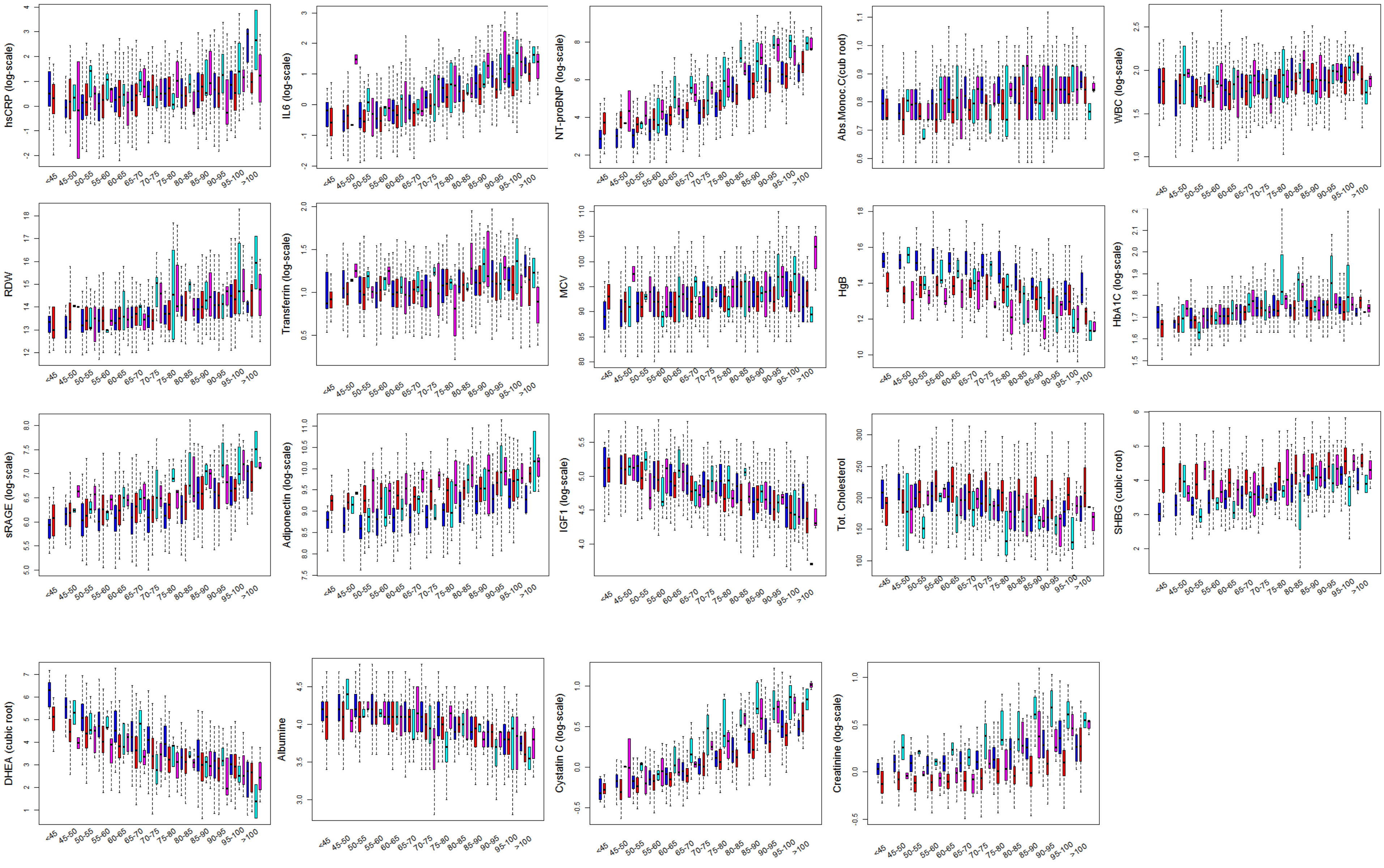

Supplement Figure S18_h: Cluster 8

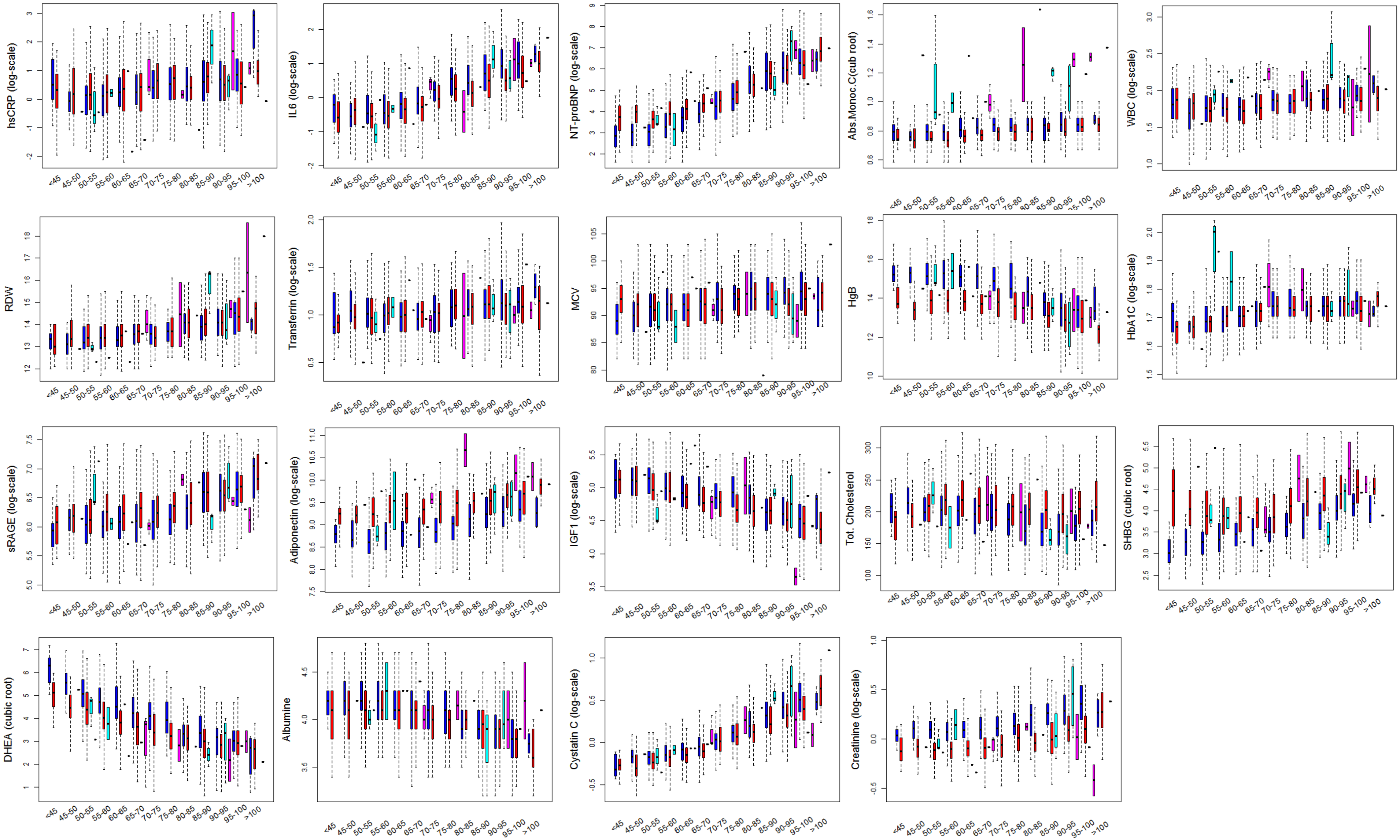

Supplement Figure S18_k: Cluster 9

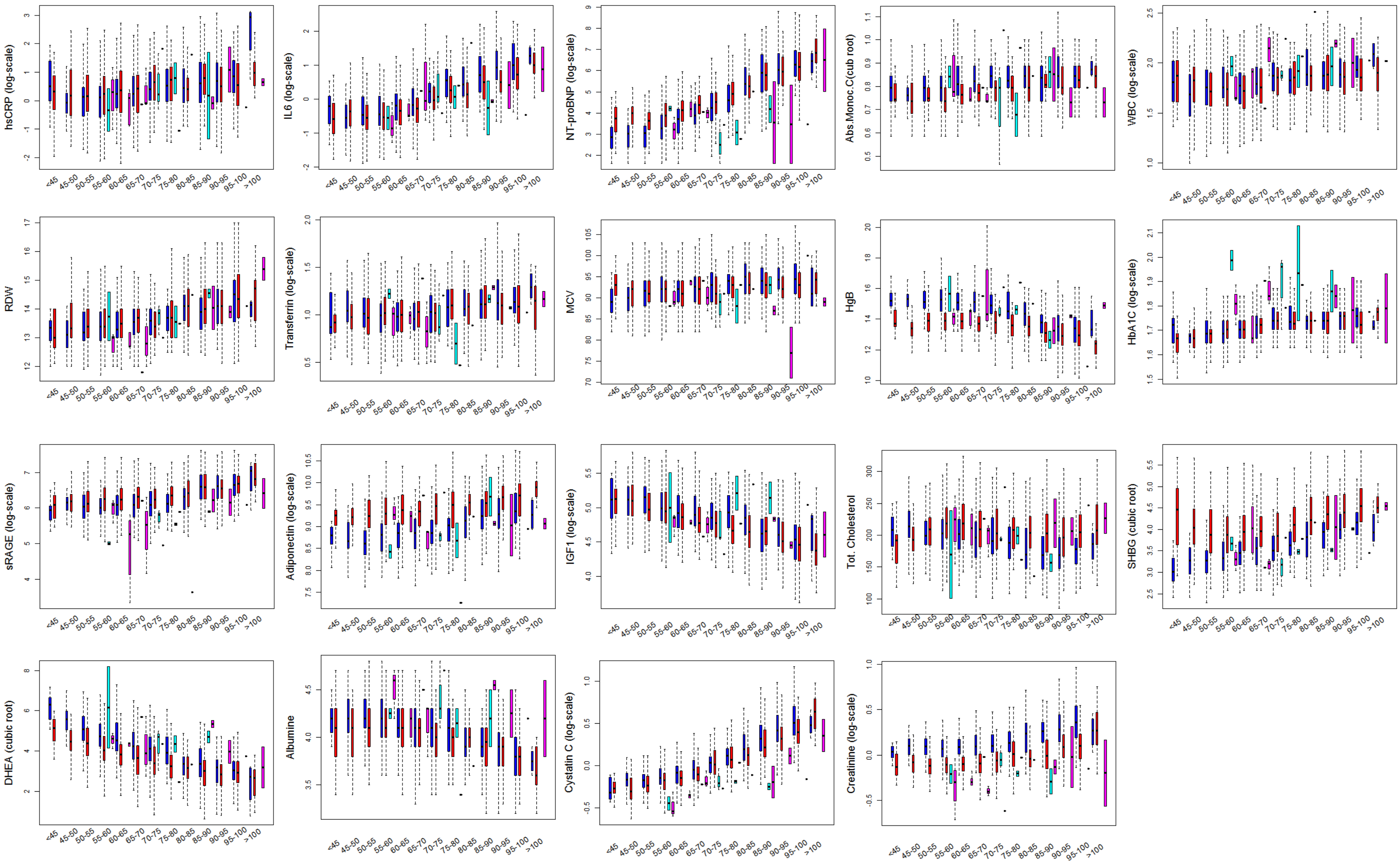

Supplement Figure S18_j: Cluster 10

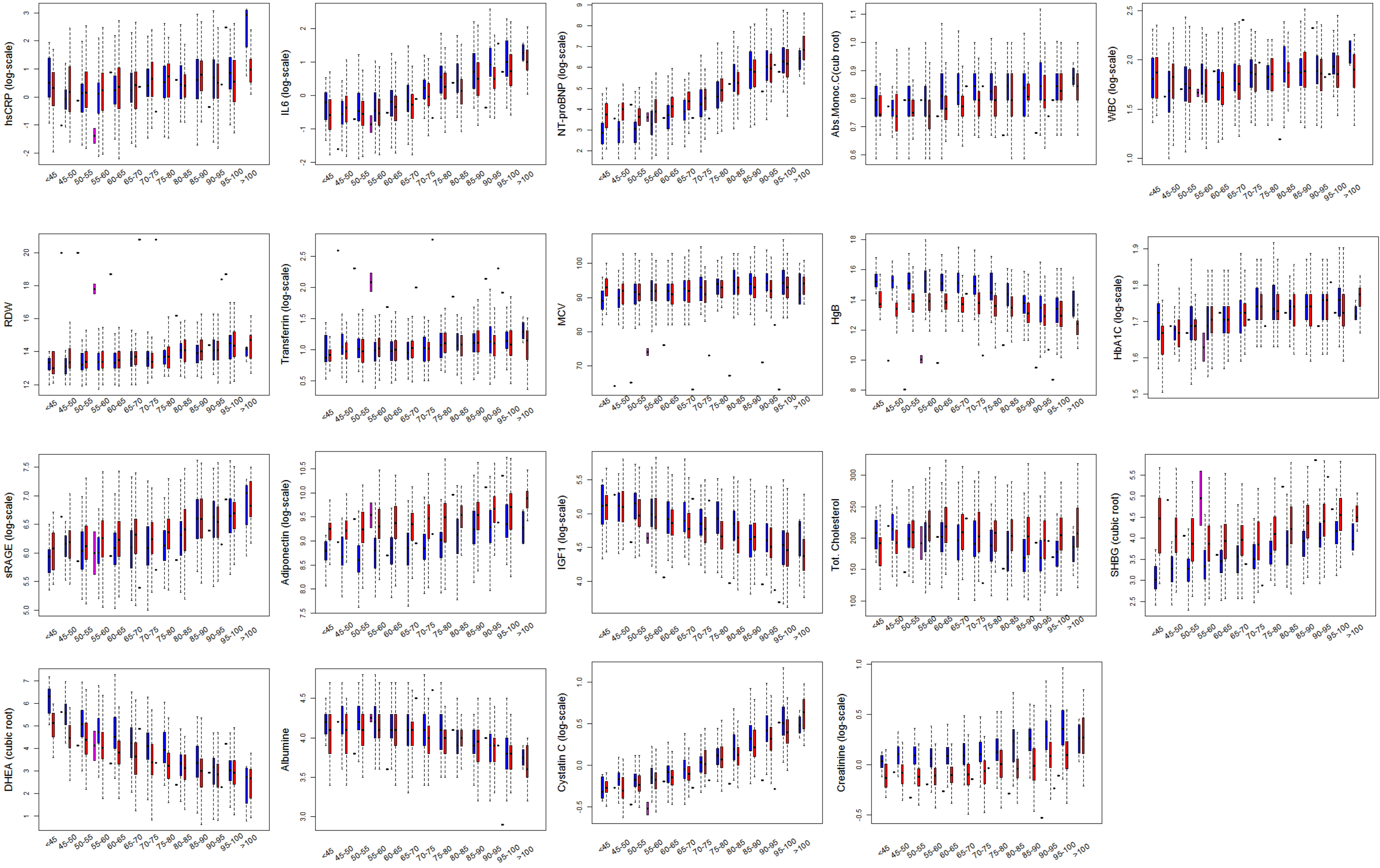

Supplement Figure S18_k: Cluster 11

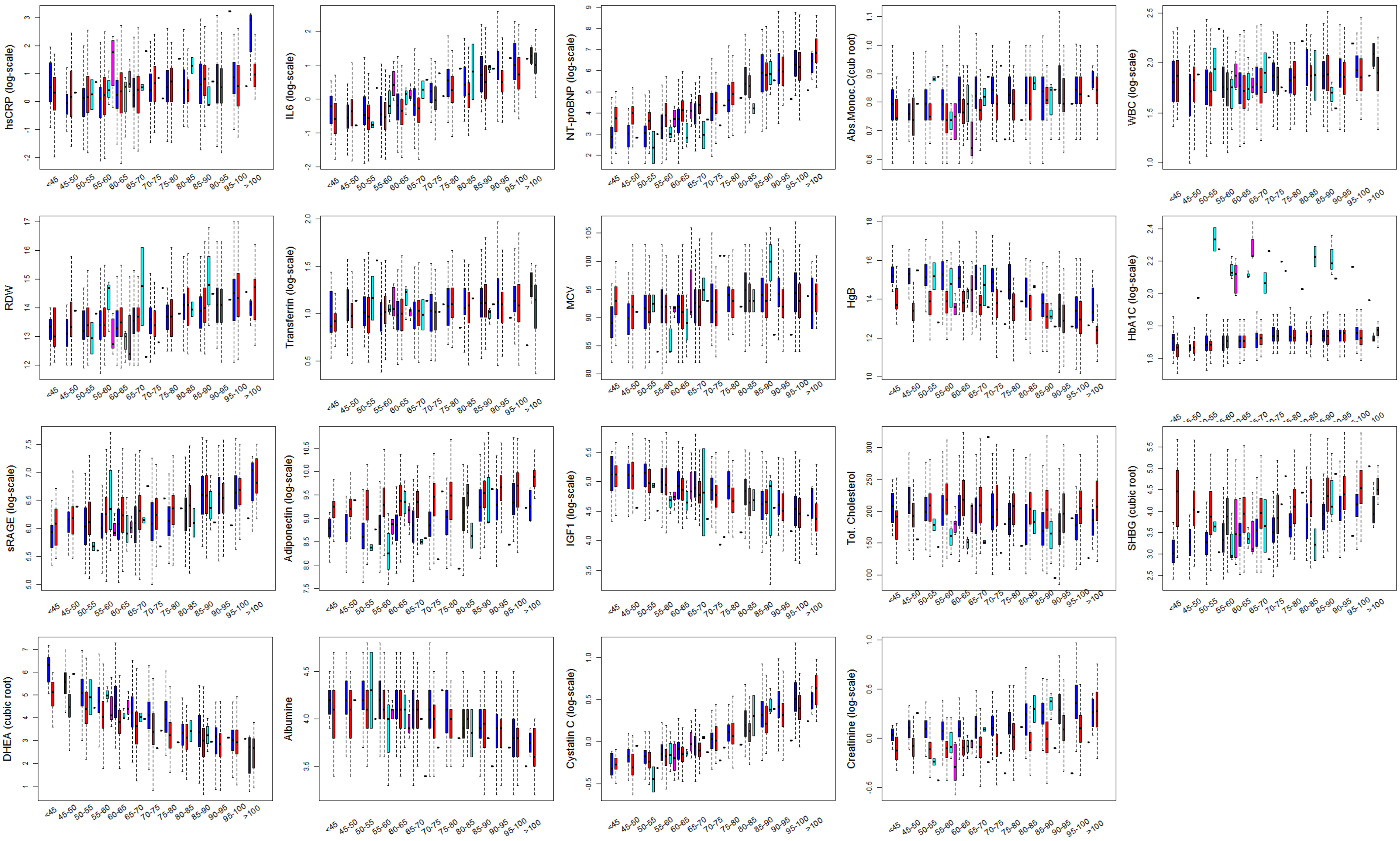

Supplement Figure S18_m: Cluster 12

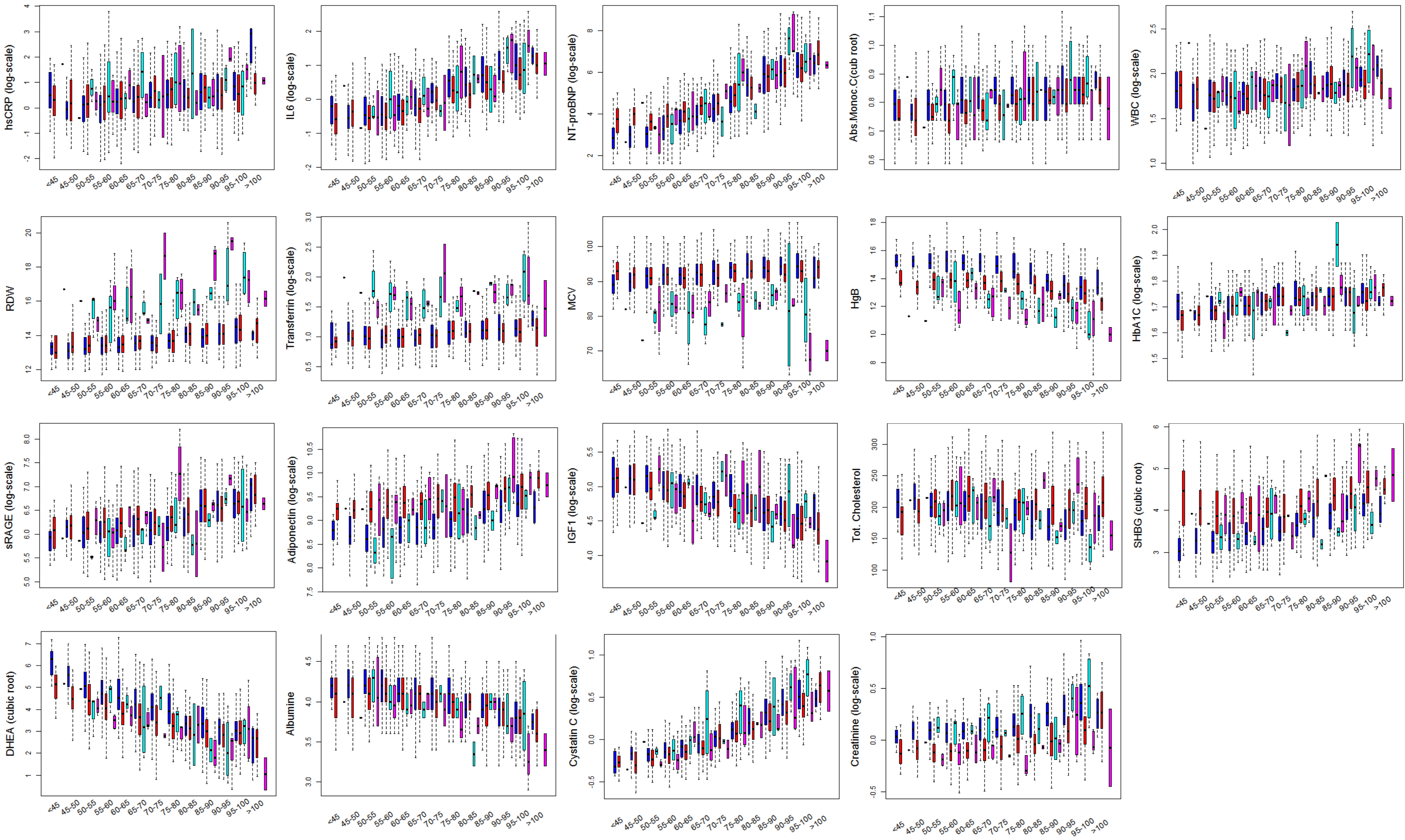

Supplement Figure S18_n: Cluster 13

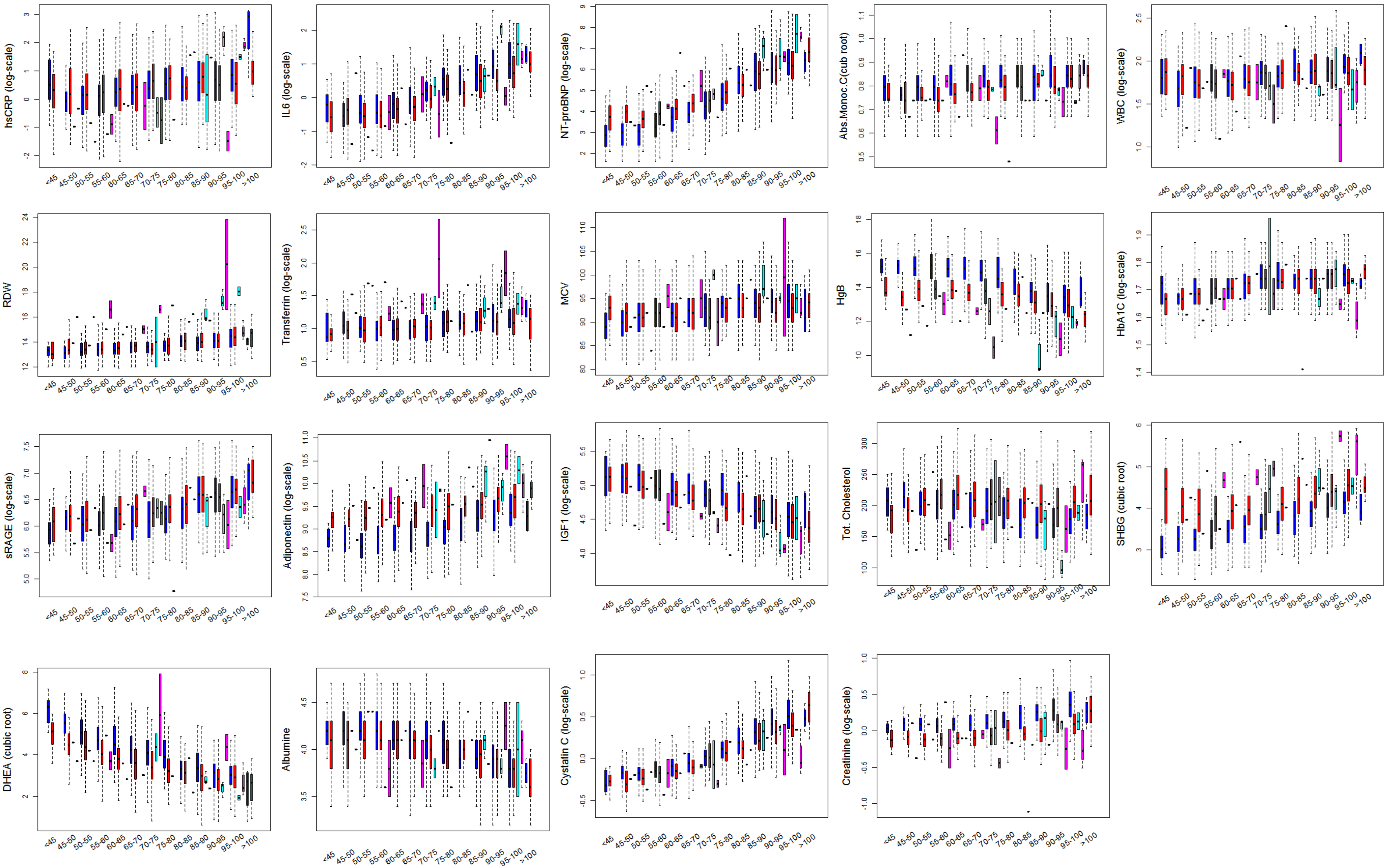

Supplement Figure S18_0: Cluster 14

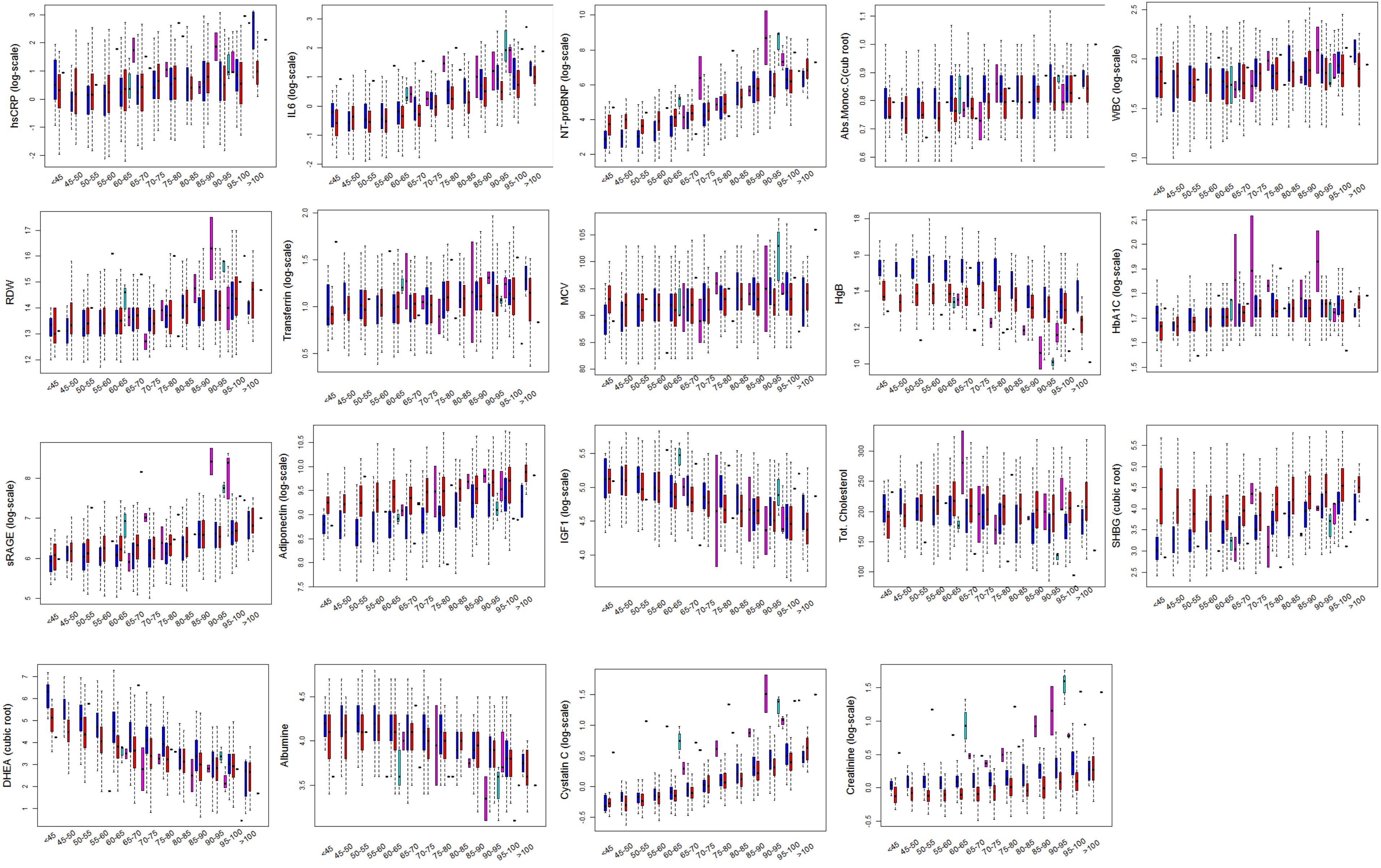

Supplement Figure S18_p: Cluster 15

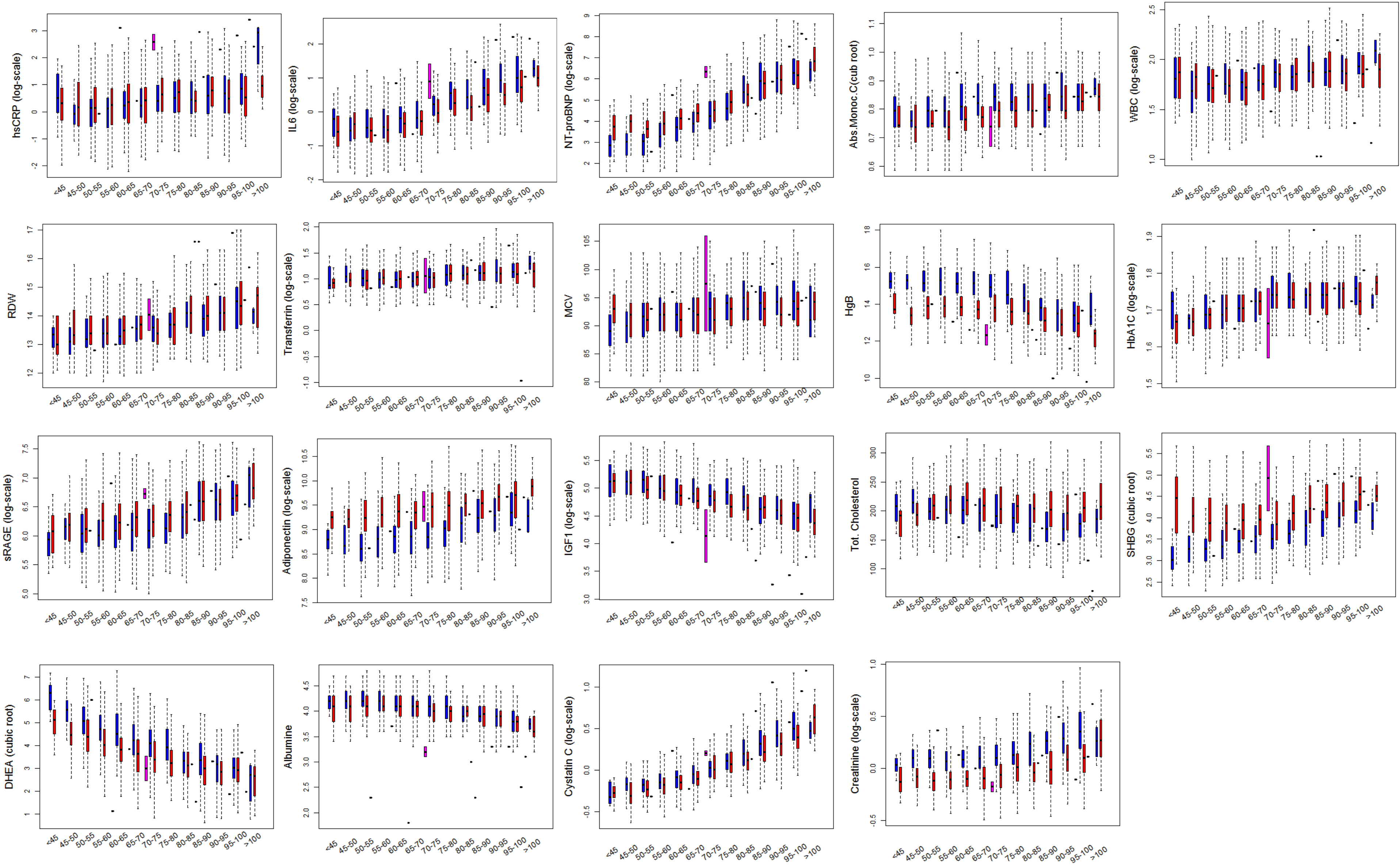

Supplement Figure S18_p: Cluster 16

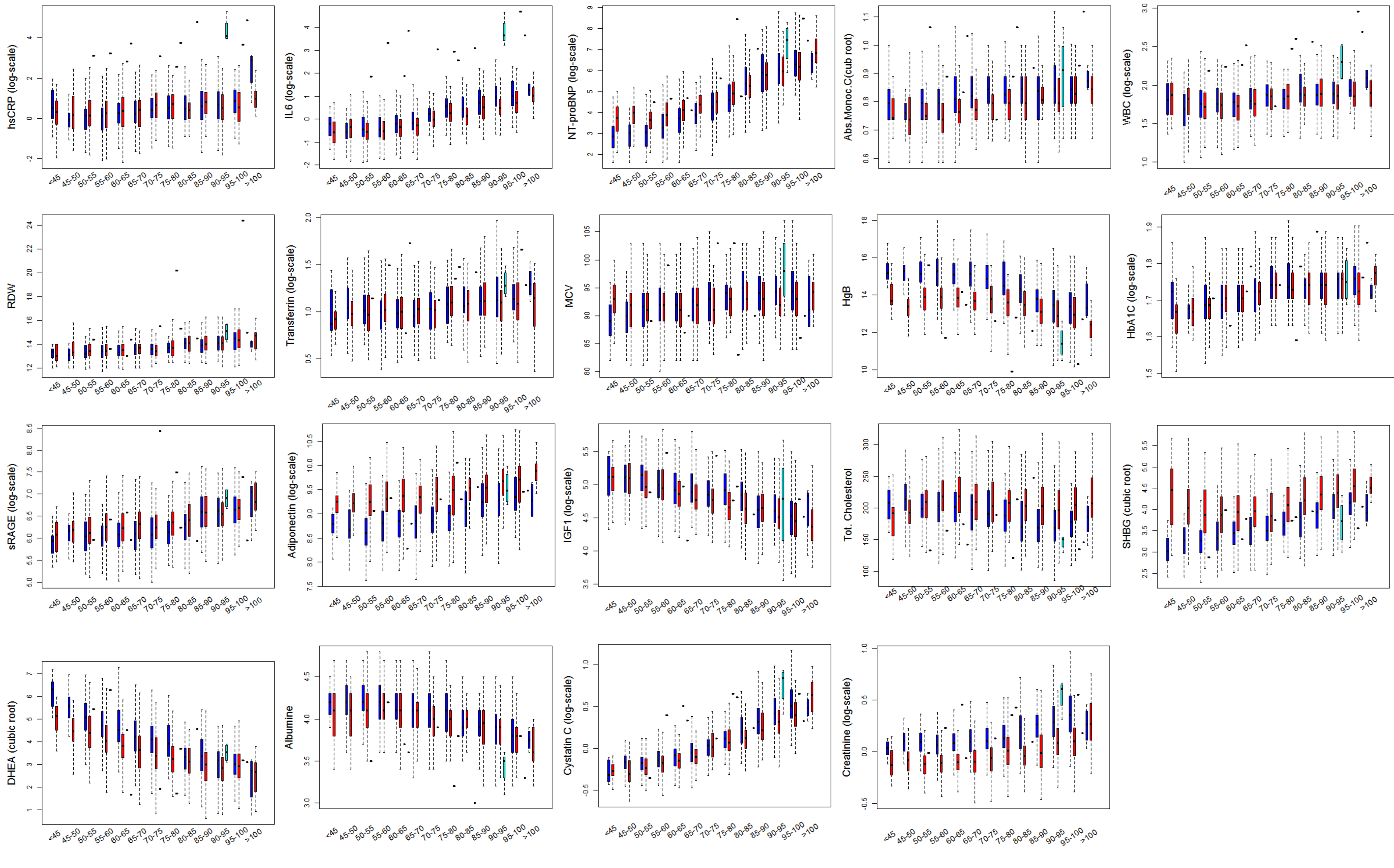

Supplement Figure S18_p: Cluster 17

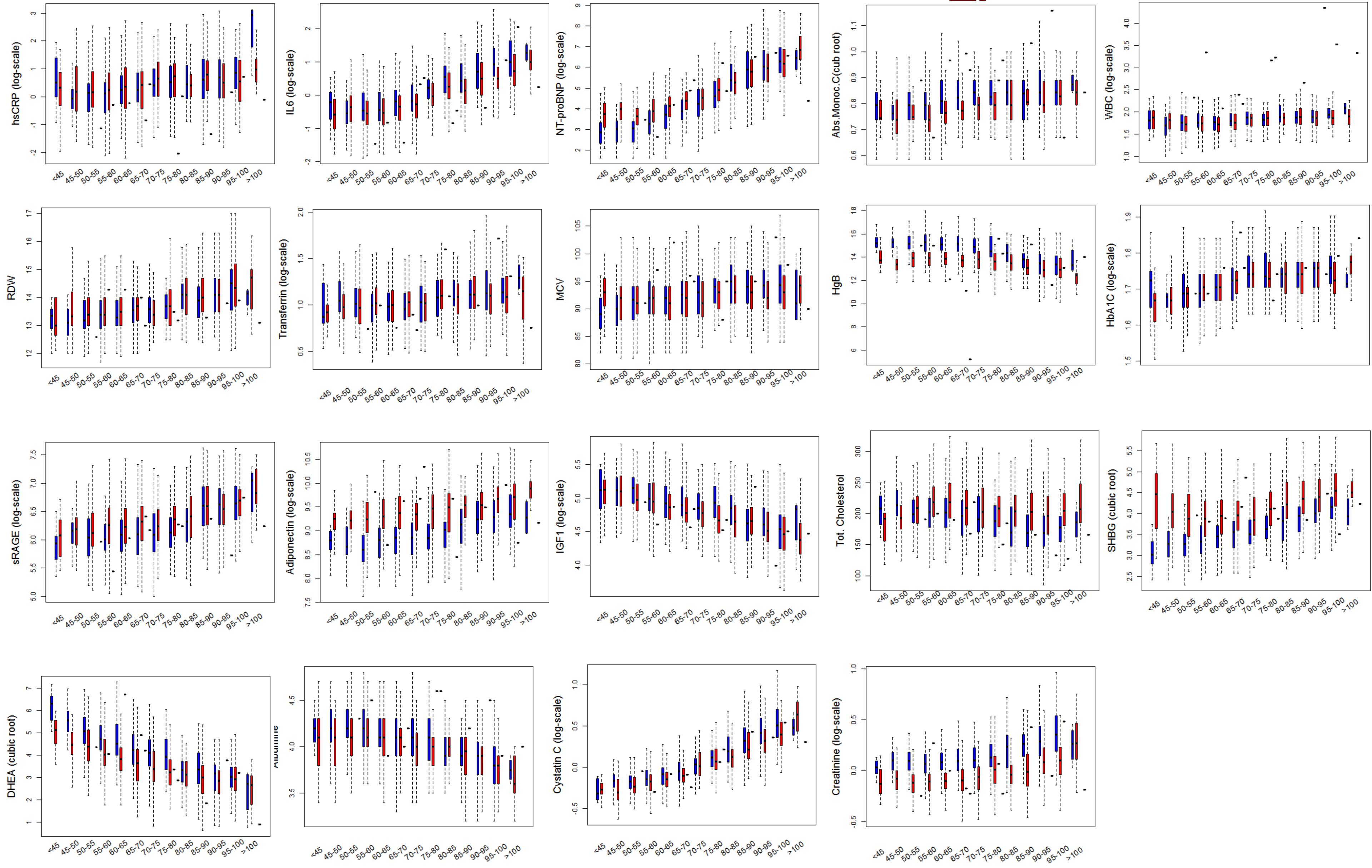

Supplement Figure S19: Example of Lab-to-Lab Bias in Albumin Measurements

Red=FHS-gen-1 ex20;
Green = FHS-gen 2 exam 7;
Blue= FHS-gen3 – ex1;
Pale blue = LLFS;
Normal range 3.5; 5.4

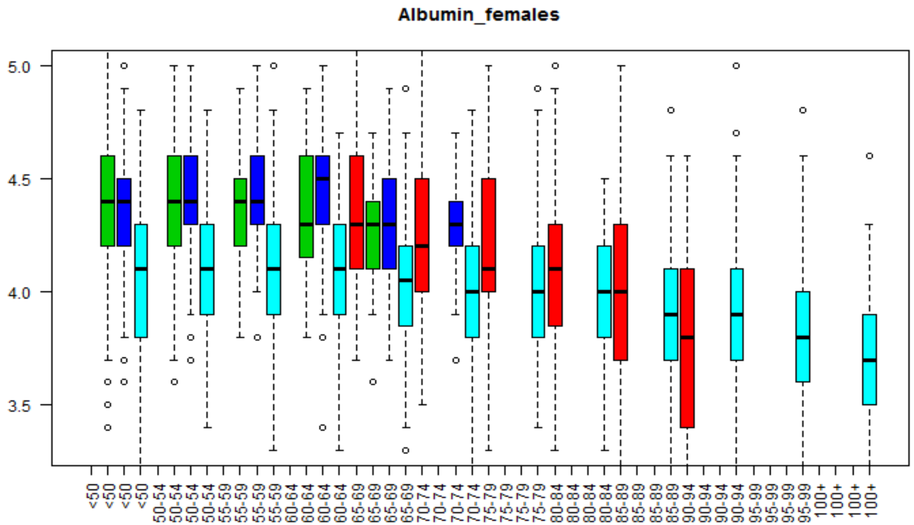

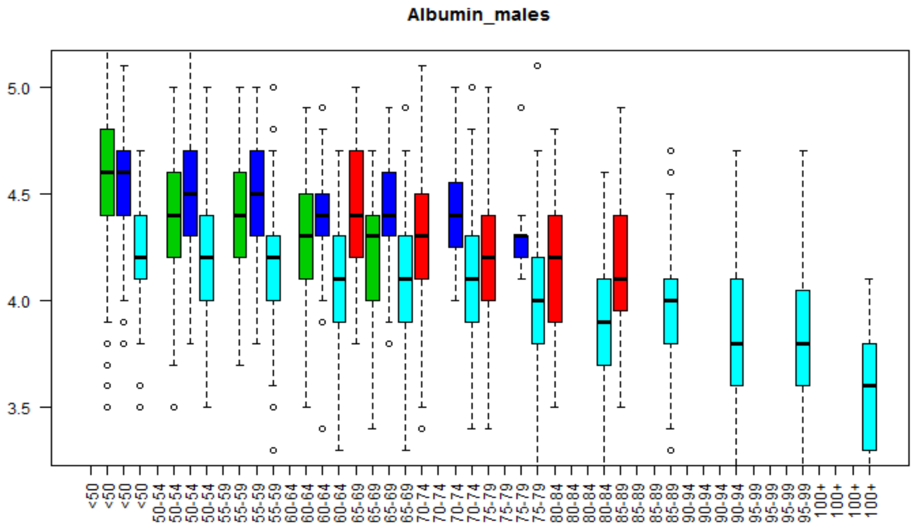

Measurements in FHS tend to be systematically higher at younger ages (batch effect), and converge at older ages. So in older LLFS participants, we see higher than expected albumin. The batch effect is consistent with what we see in NHANES

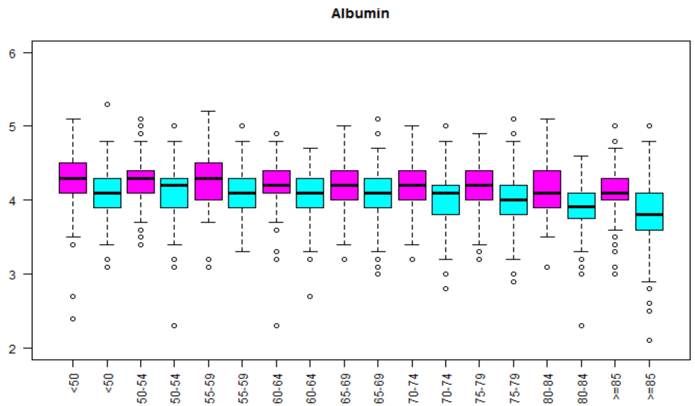

NHANES

Supplement Figure S20-a: Externally Standardized Adiponectin

Females | Males

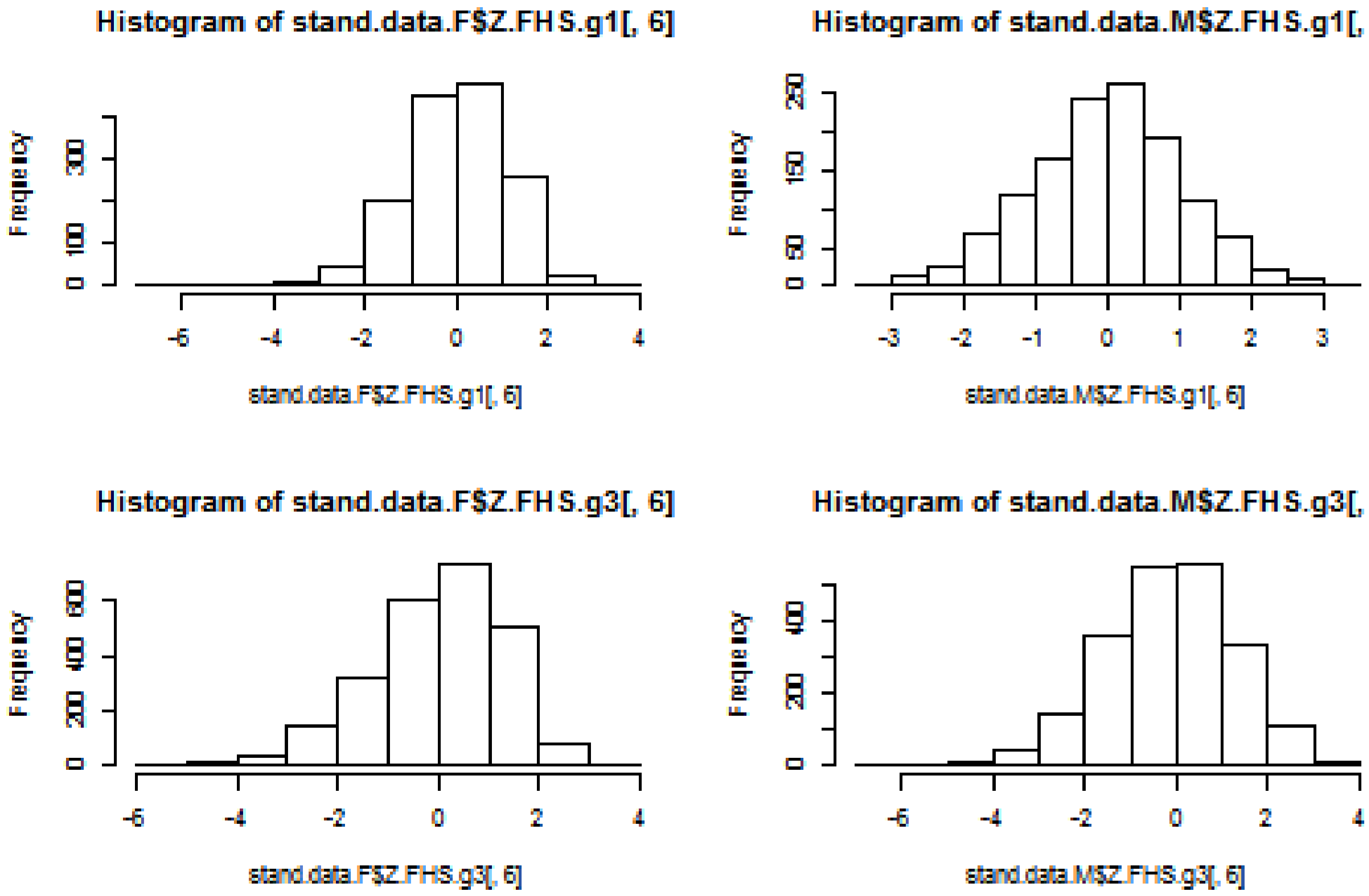

Supplement Figure S20-b: Externally Standardized Albumin

Females

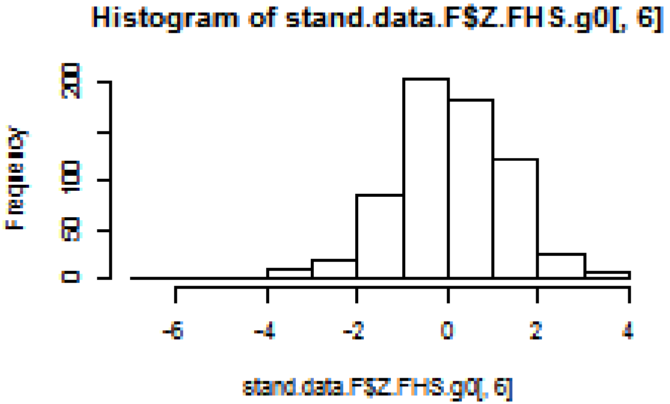

Males

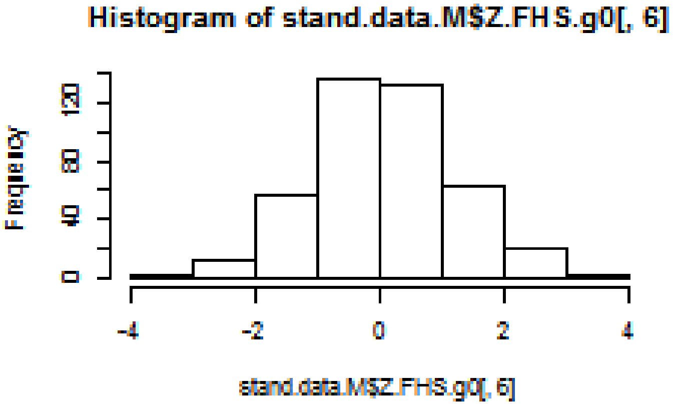

Supplement Figure S20-c: Externally Standardized Abs. Monocyte Counts

Females

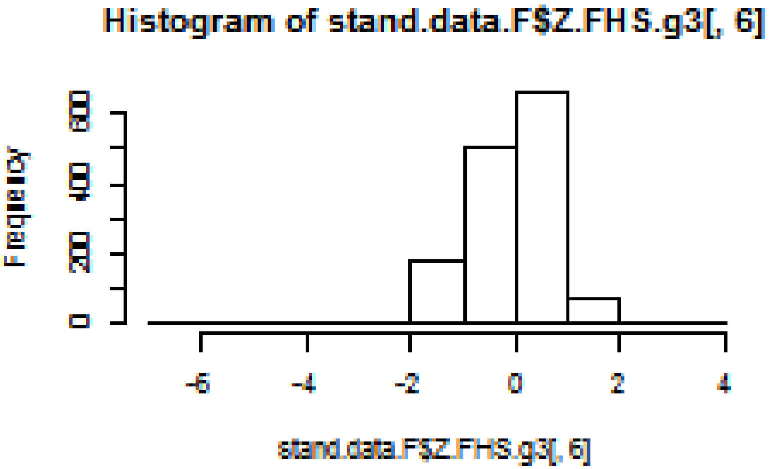

Males

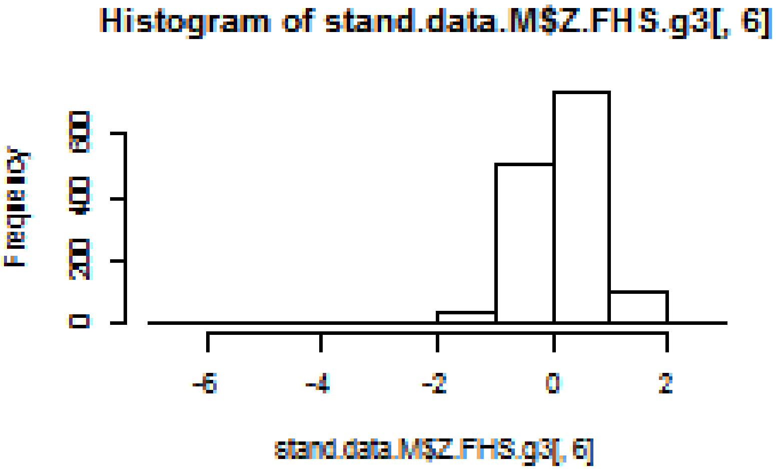

Supplement Figure S20-d: Externally Standardized NT-proBNP

Females | Males

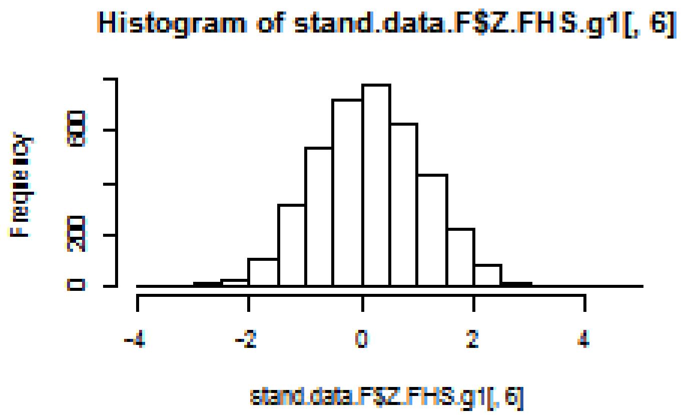

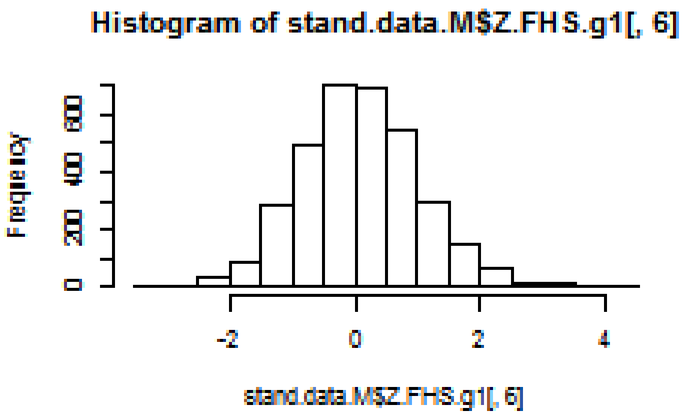

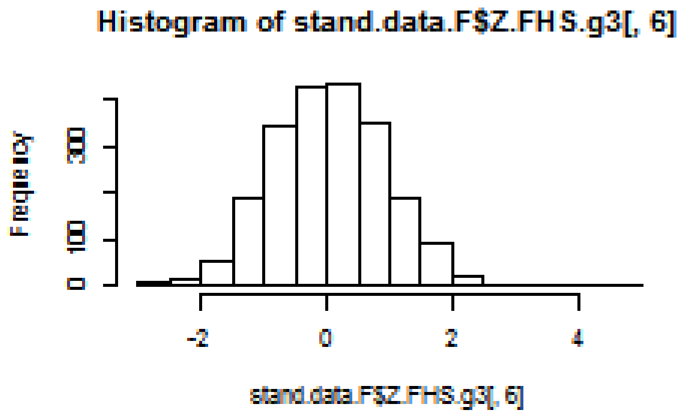

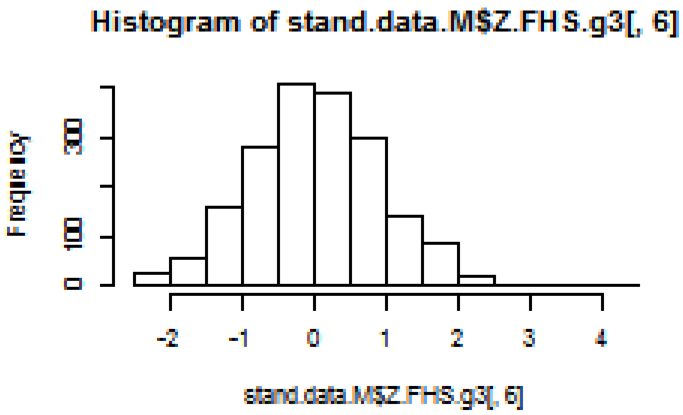

Supplement Figure S20-e: Externally Standardized Total Cholesterol

Females | Males

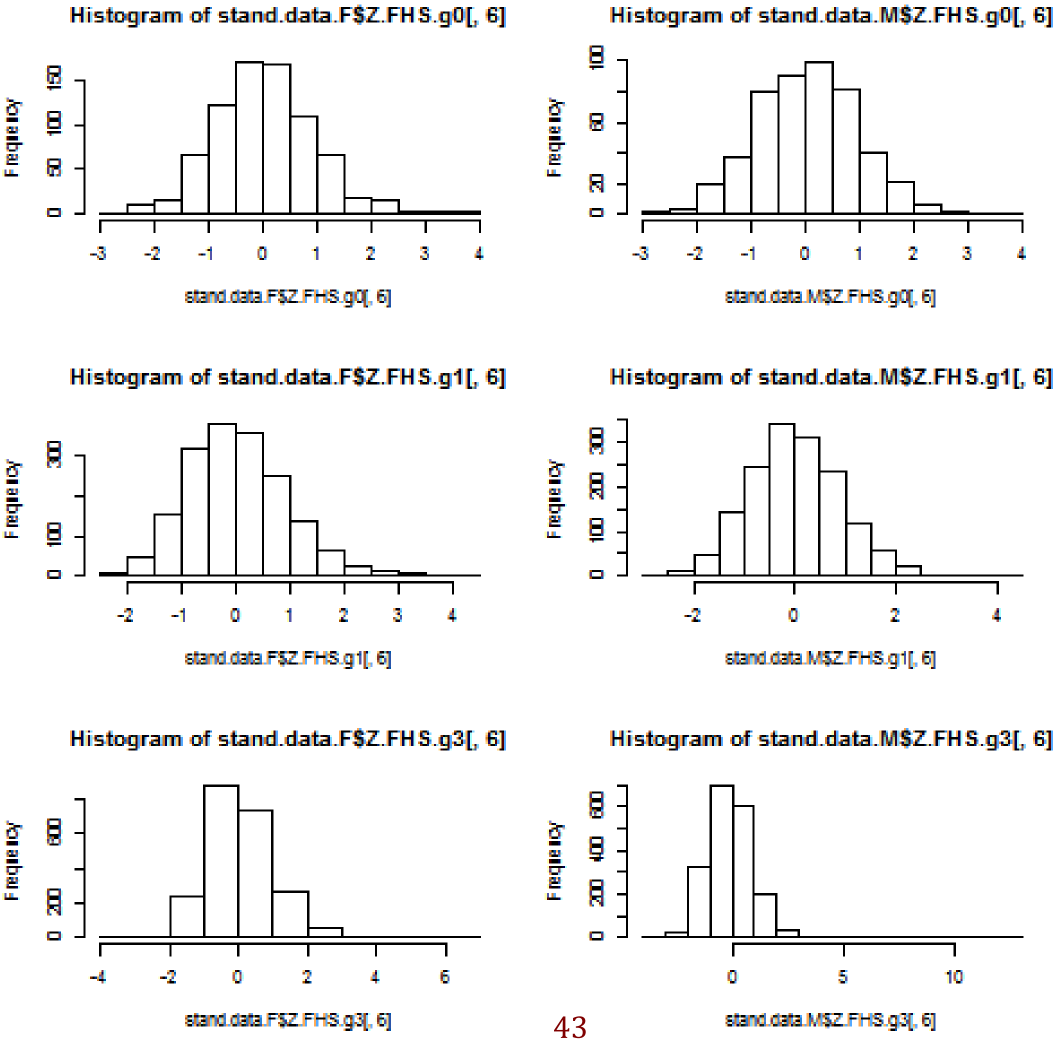

Supplement Figure S20-f: Externally Standardized Creatinine

Females | Males

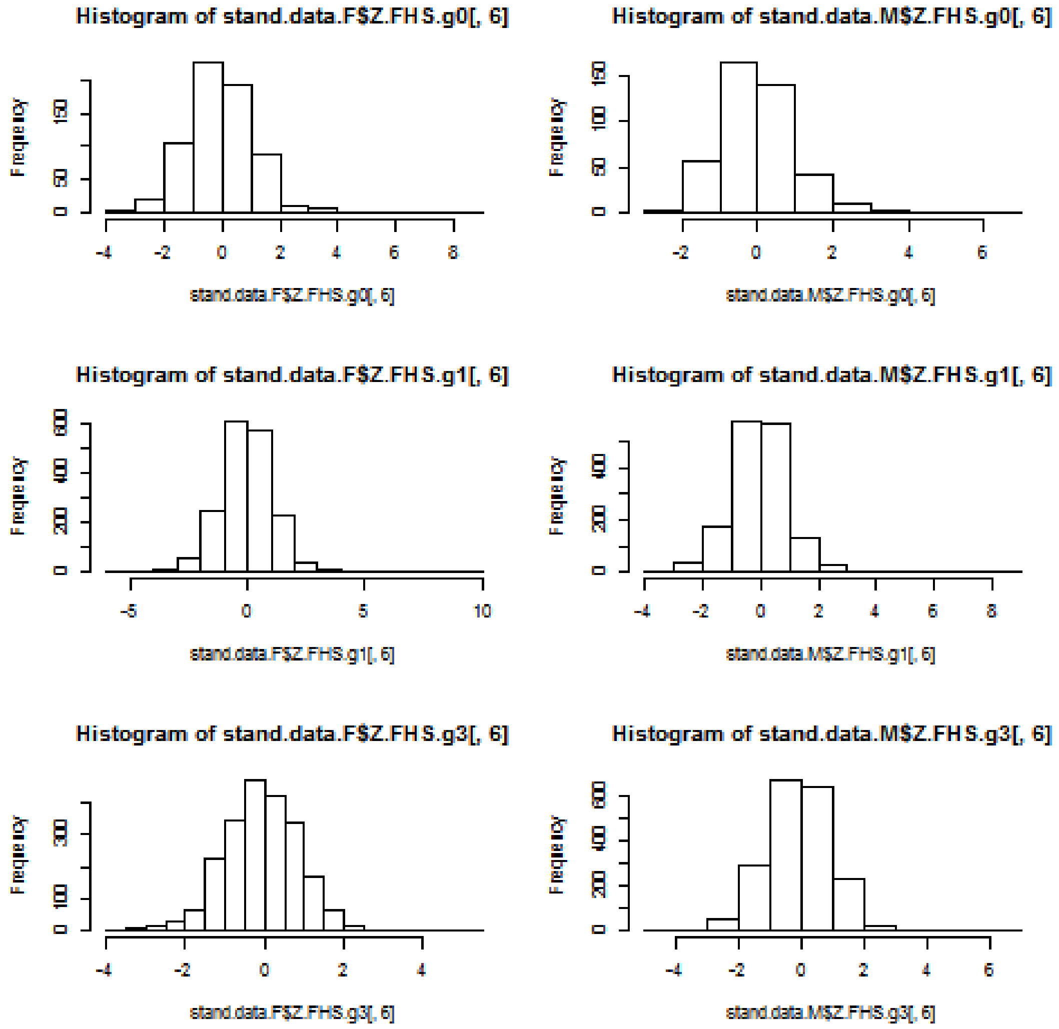

Supplement Figure S20-g: Externally Standardized CRP

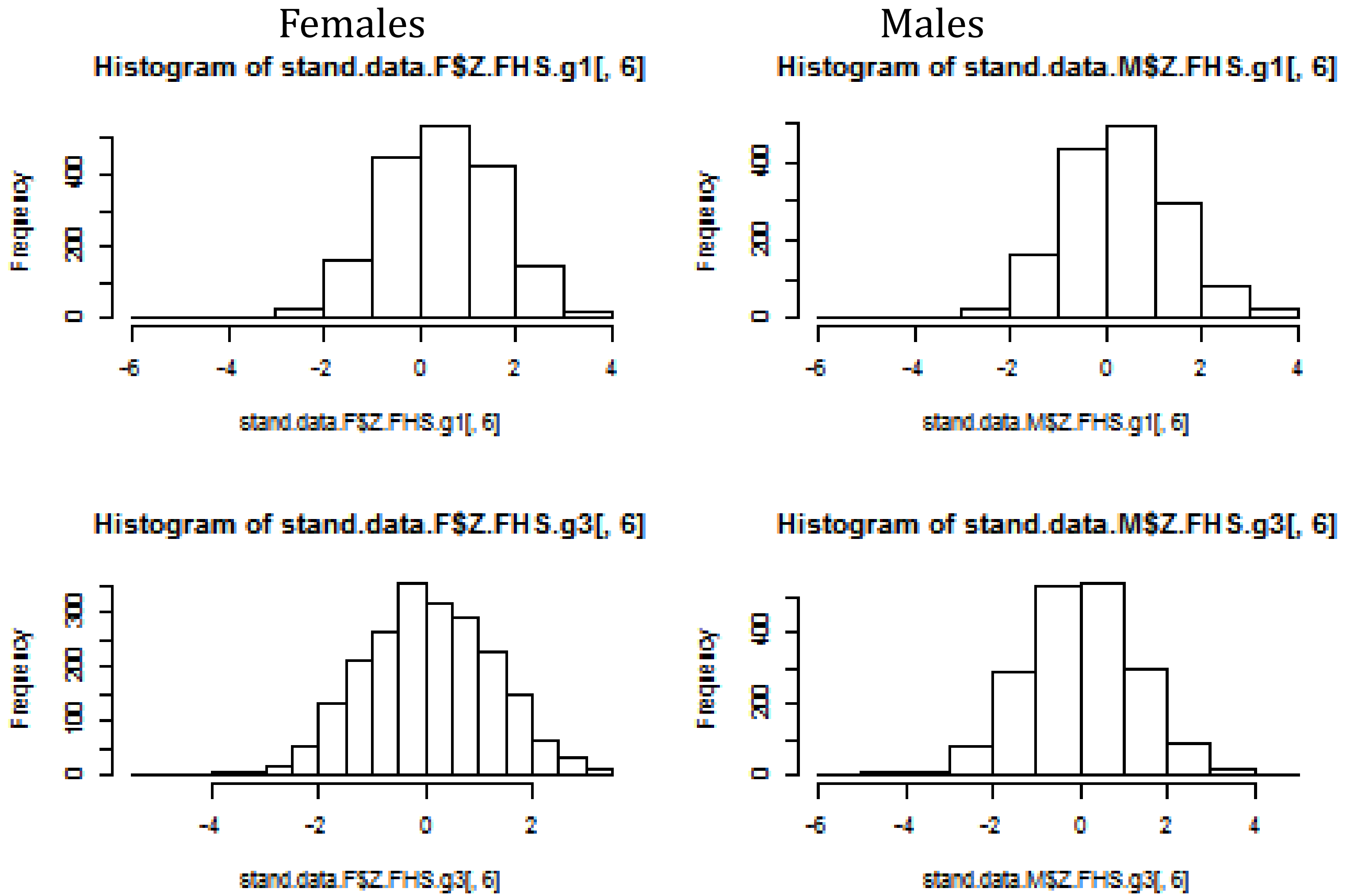

Supplement Figure S20-j: Externally Standardized Cystatin

Females | Males

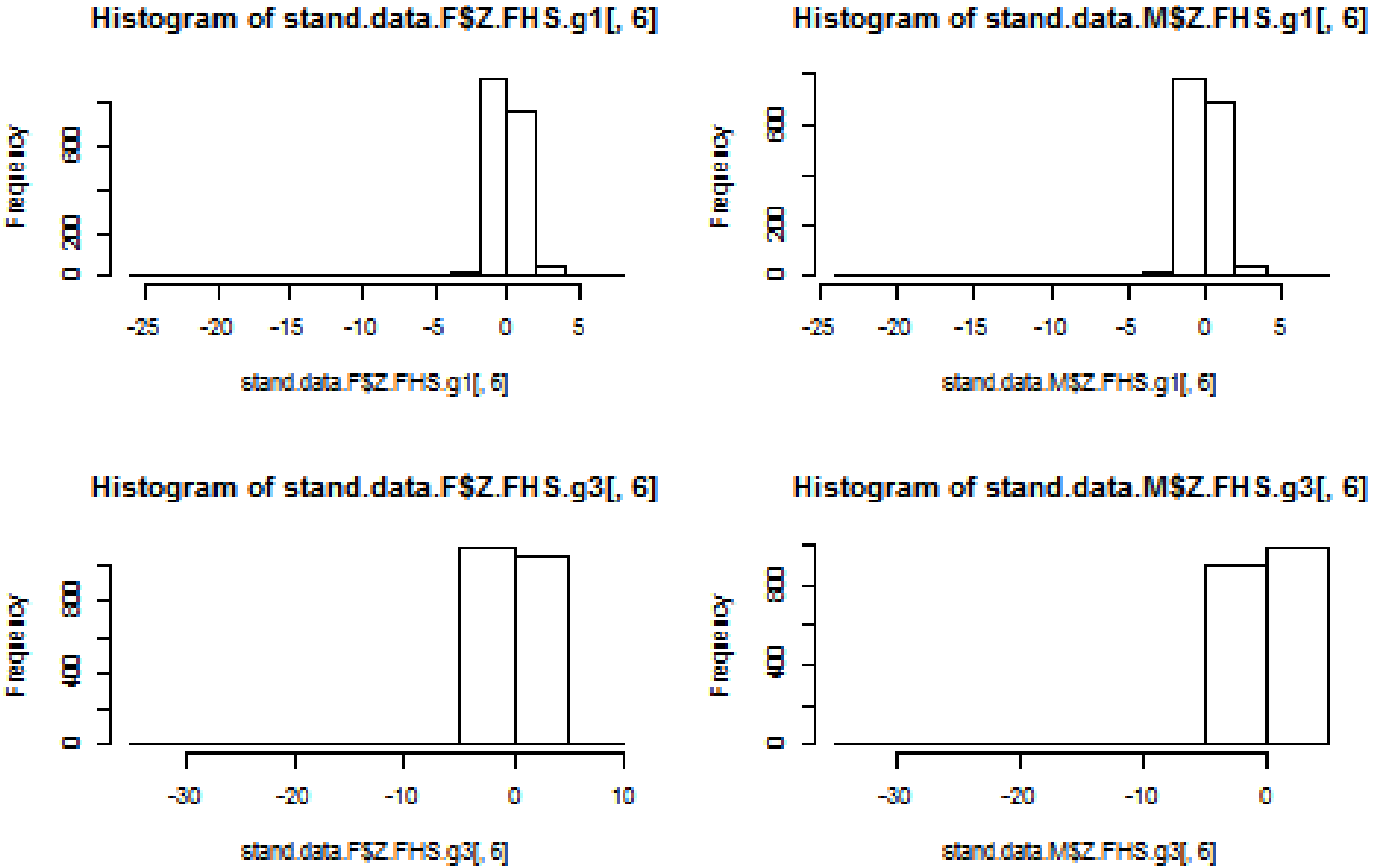

Supplement Figure S20-k: Externally Standardized DHEA

Males

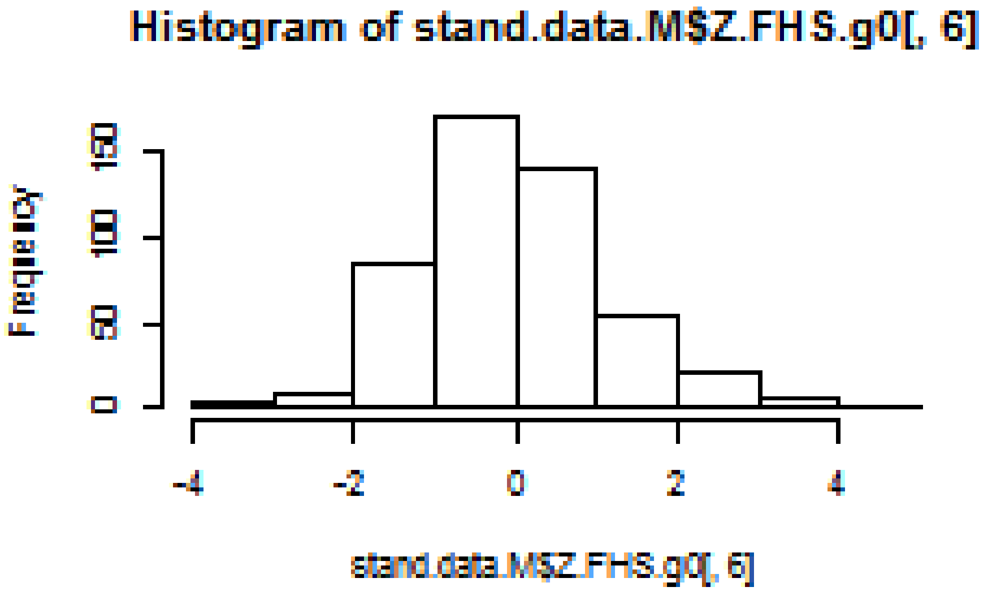

Supplement Figure S20-h: Externally Standardized HbA1C

Females | Males

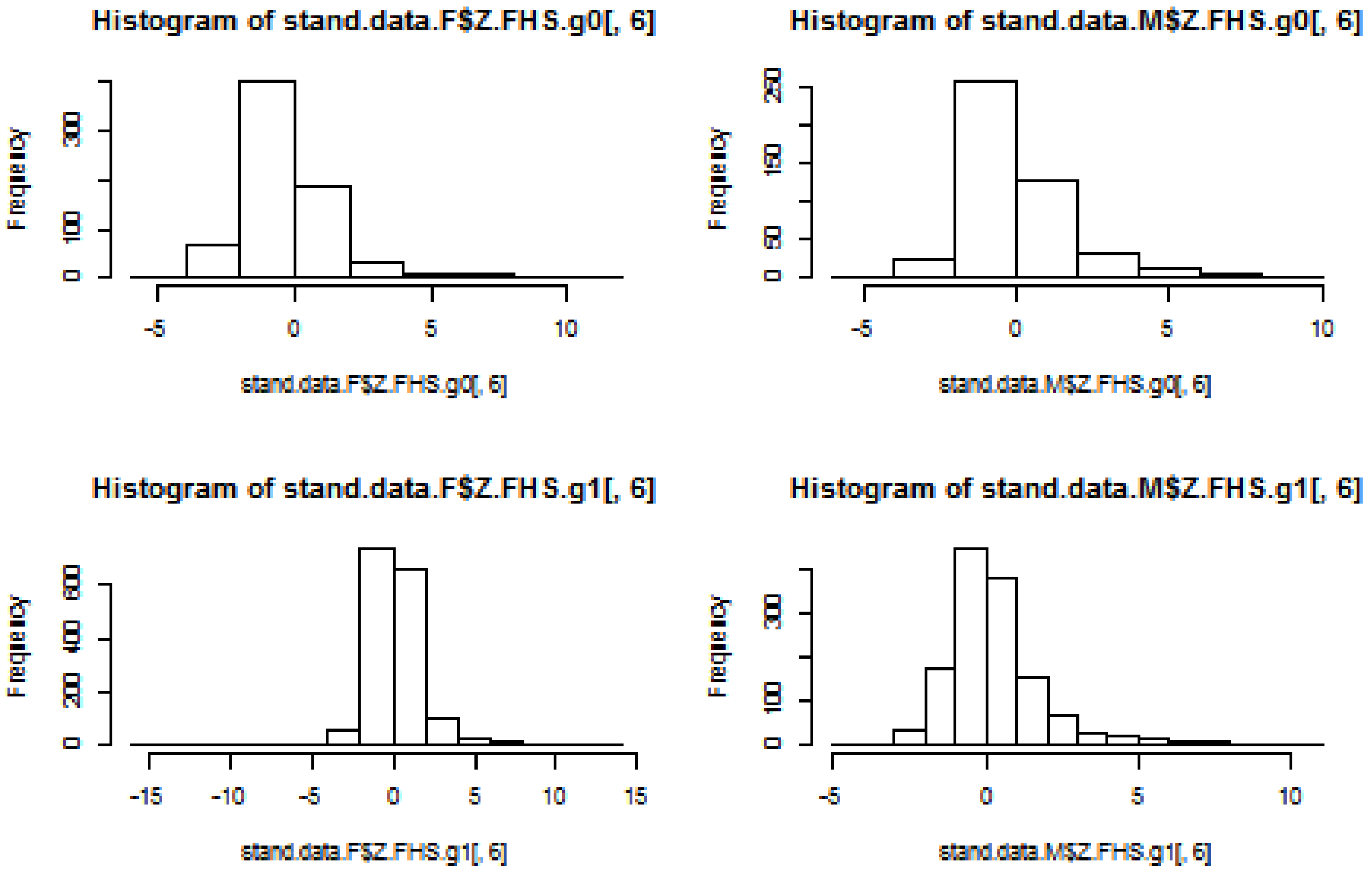

Supplement Figure S20-m: Externally Standardized HGB

Females

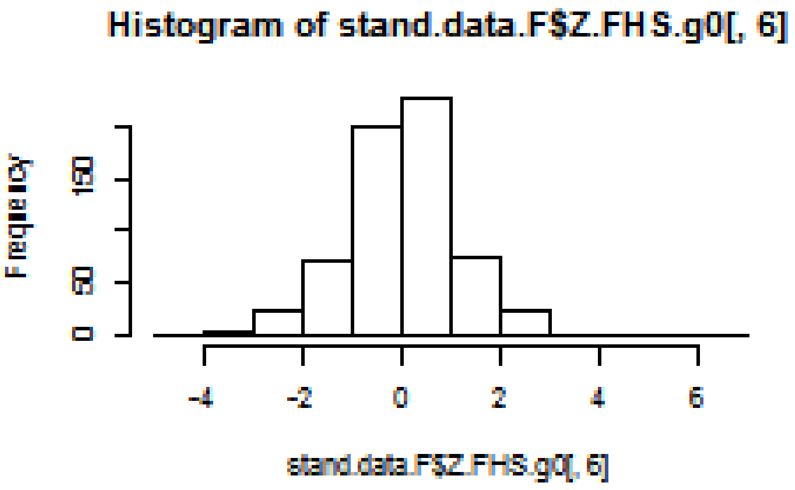

Males

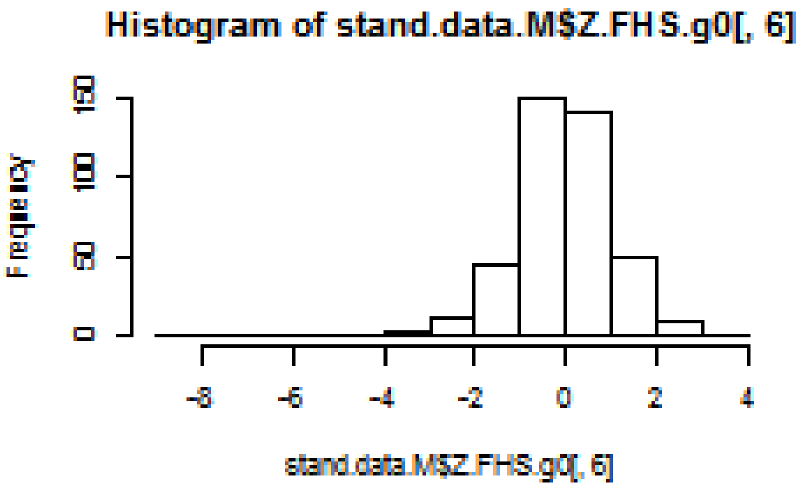

Supplement Figure S20-n: Externally Standardized IGF1

Females Males

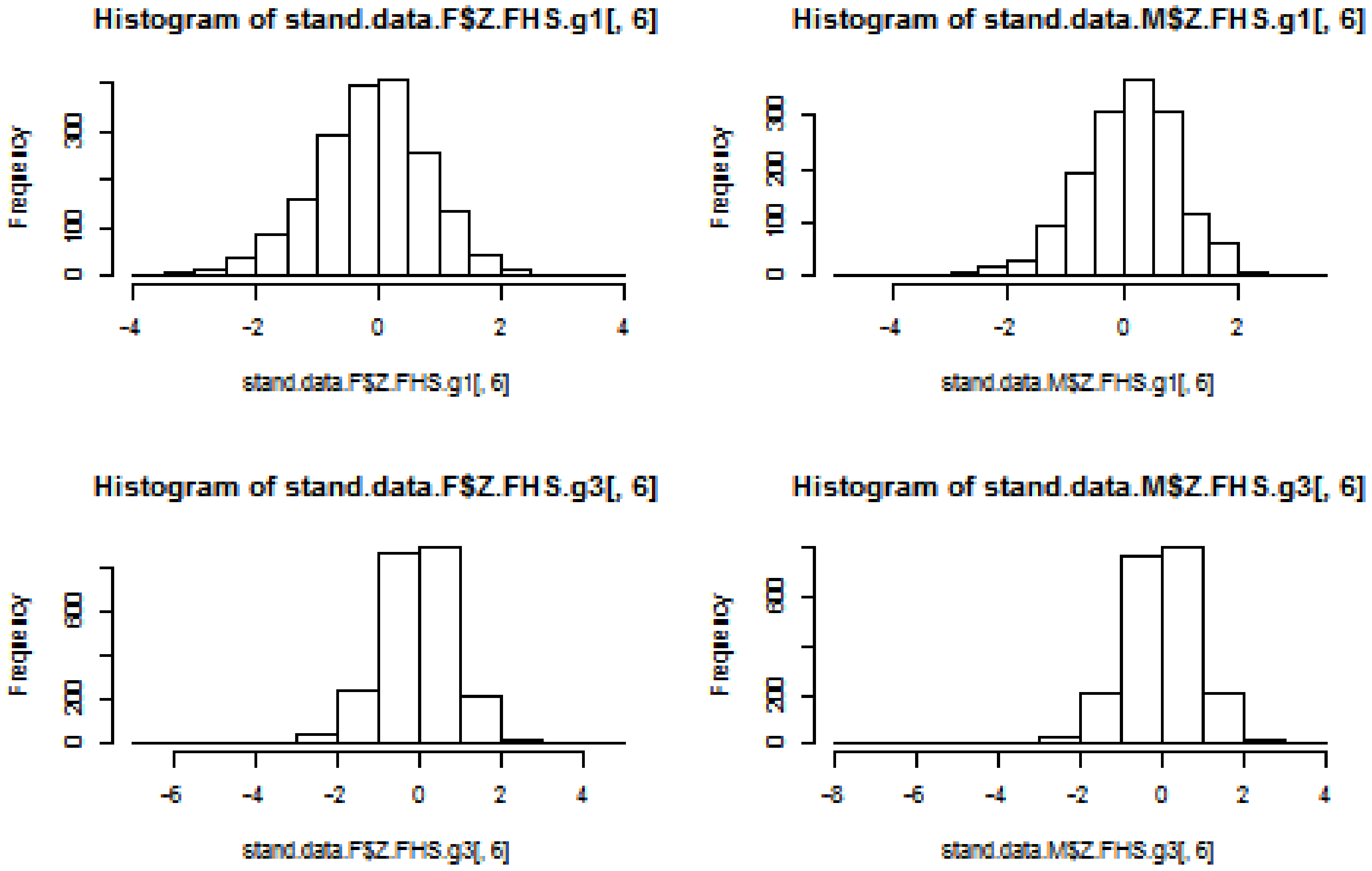

Supplement Figure S20-p: Externally Standardized IL6

Females Males

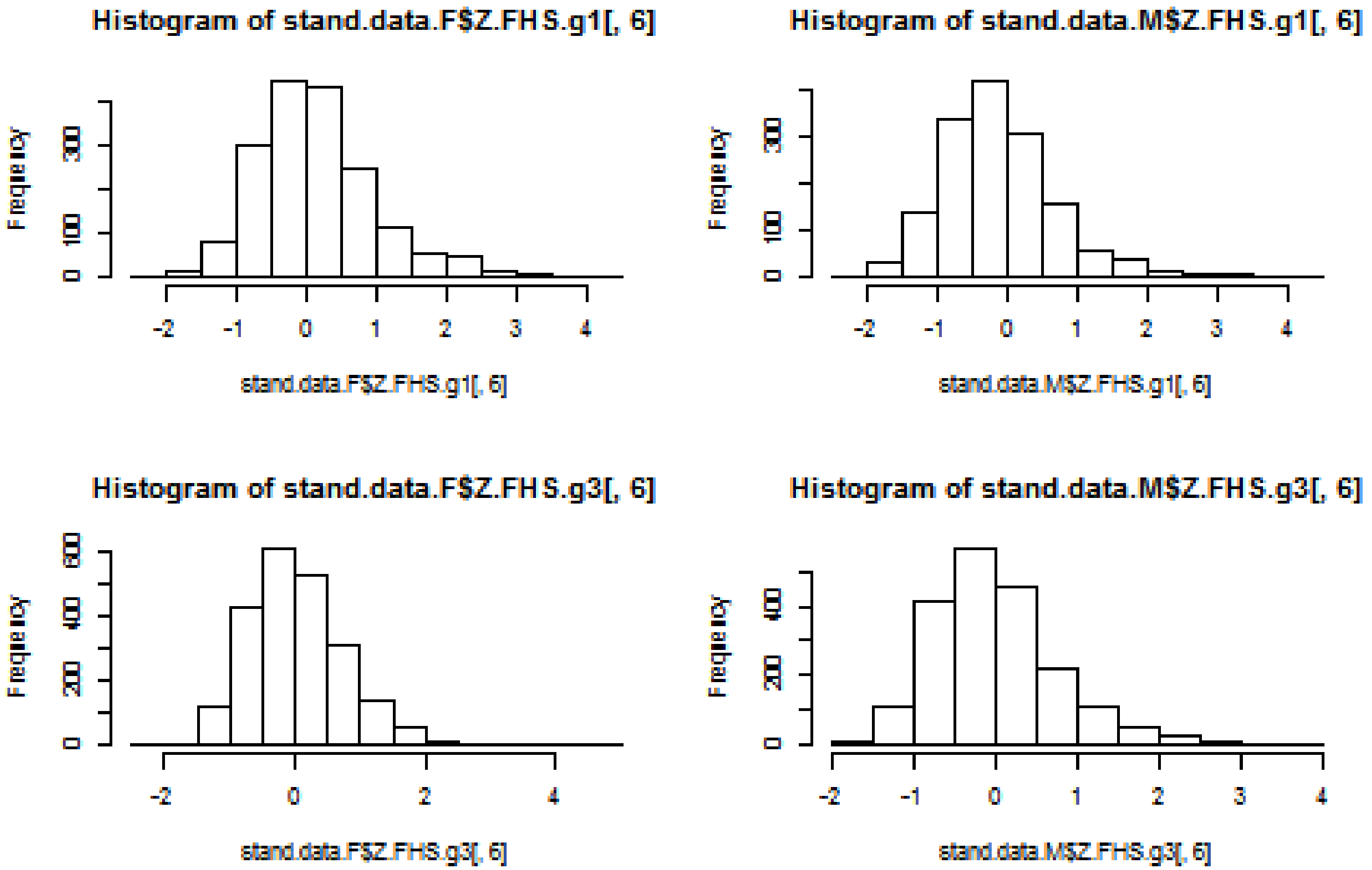

Supplement Figure S20-q: Externally Standardized MCV

Females

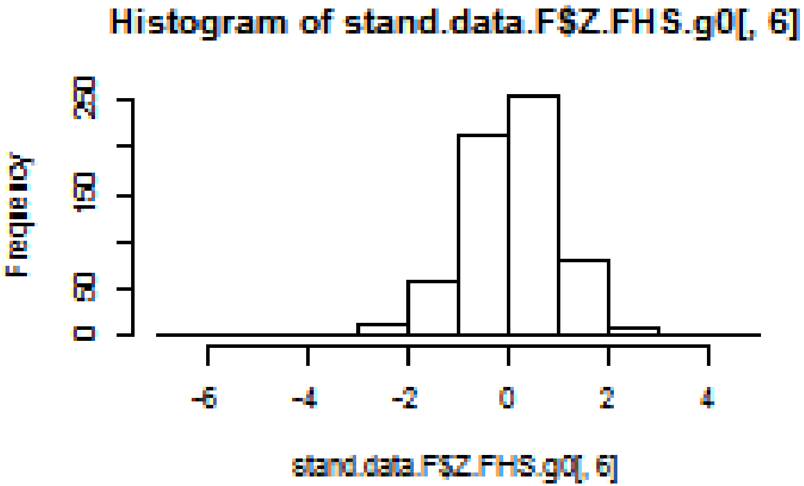

Males

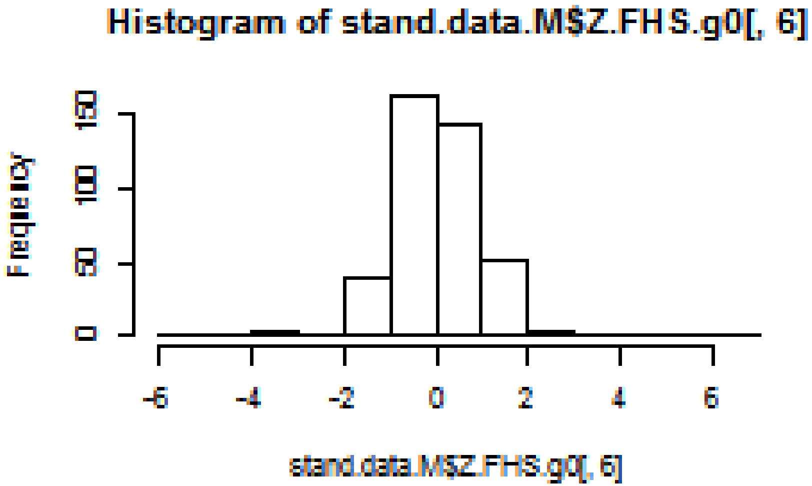

Supplement Figure S20-r: Externally Standardized SHBG

Females | Males

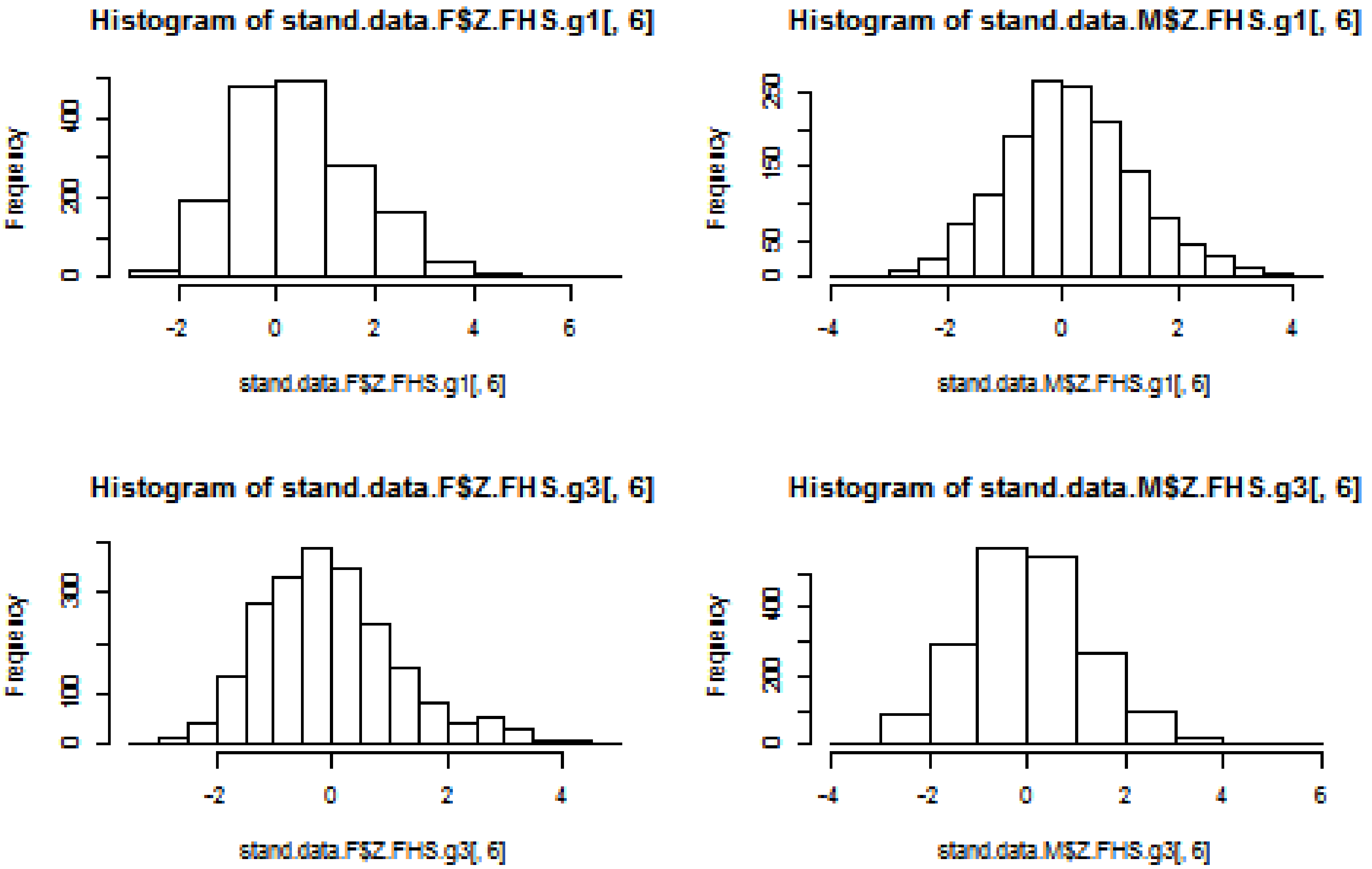

Supplement Figure S20-s: Externally Standardized SRAGE

Females | Males

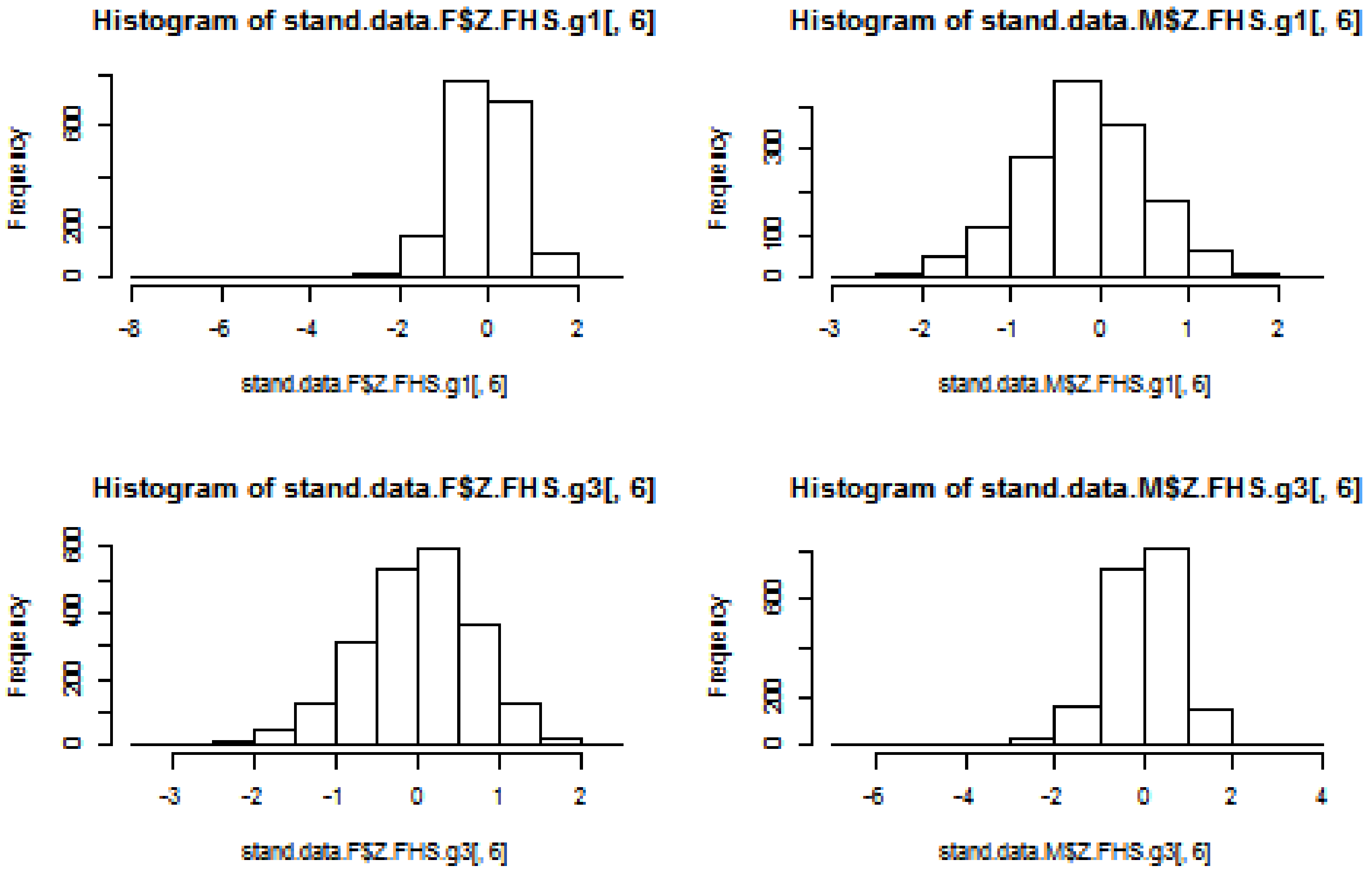

Supplement Figure S20-v: Externally Standardized Transferrin

Females

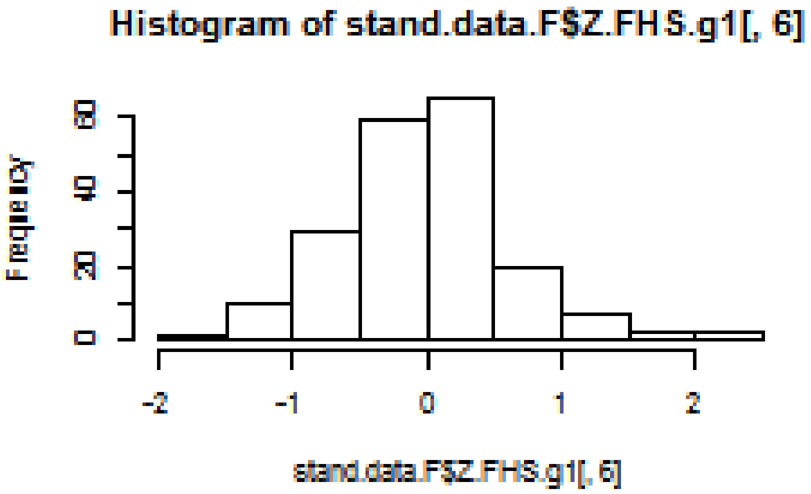

Males

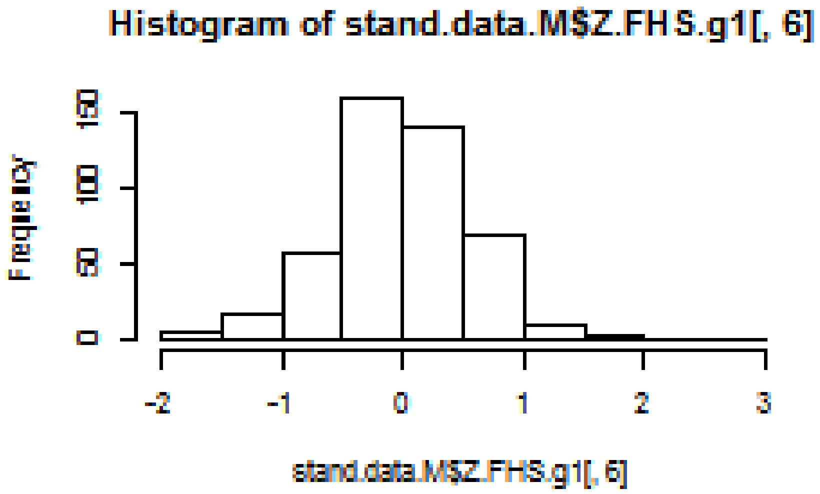

Supplement Figure S20-w: Externally Standardized WBC

Females

Males

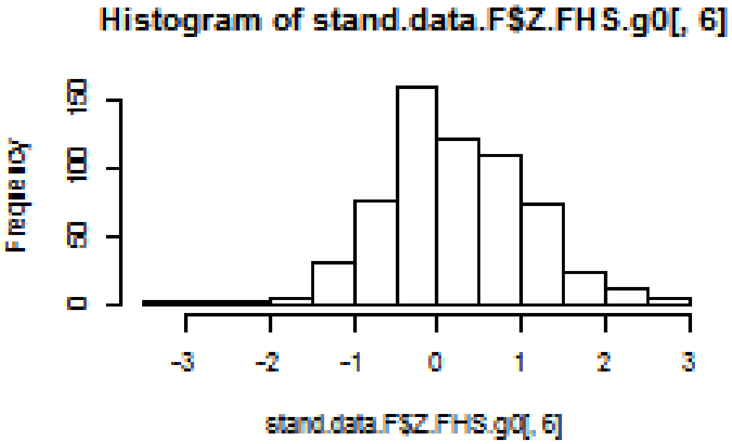

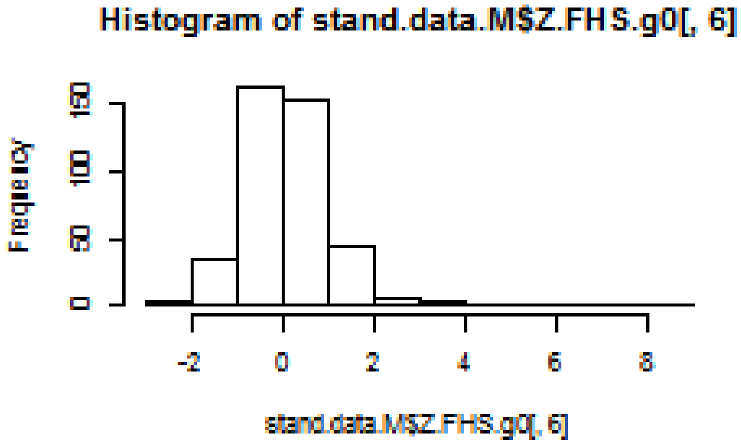

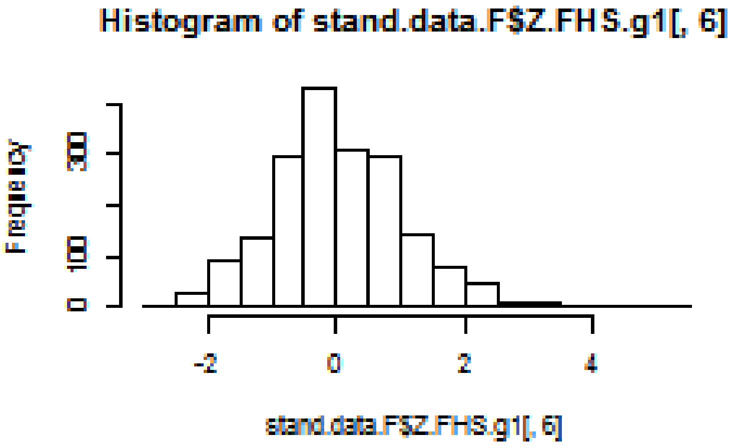

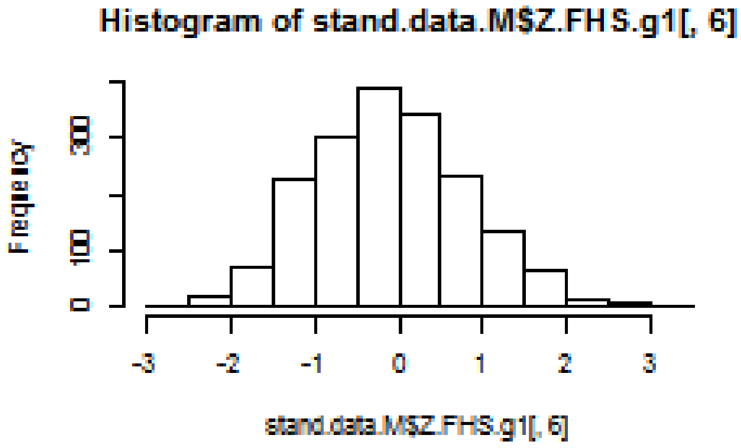

Supplement Figure S21: Distribution of Biomarker Signatures in LLFS and FHS

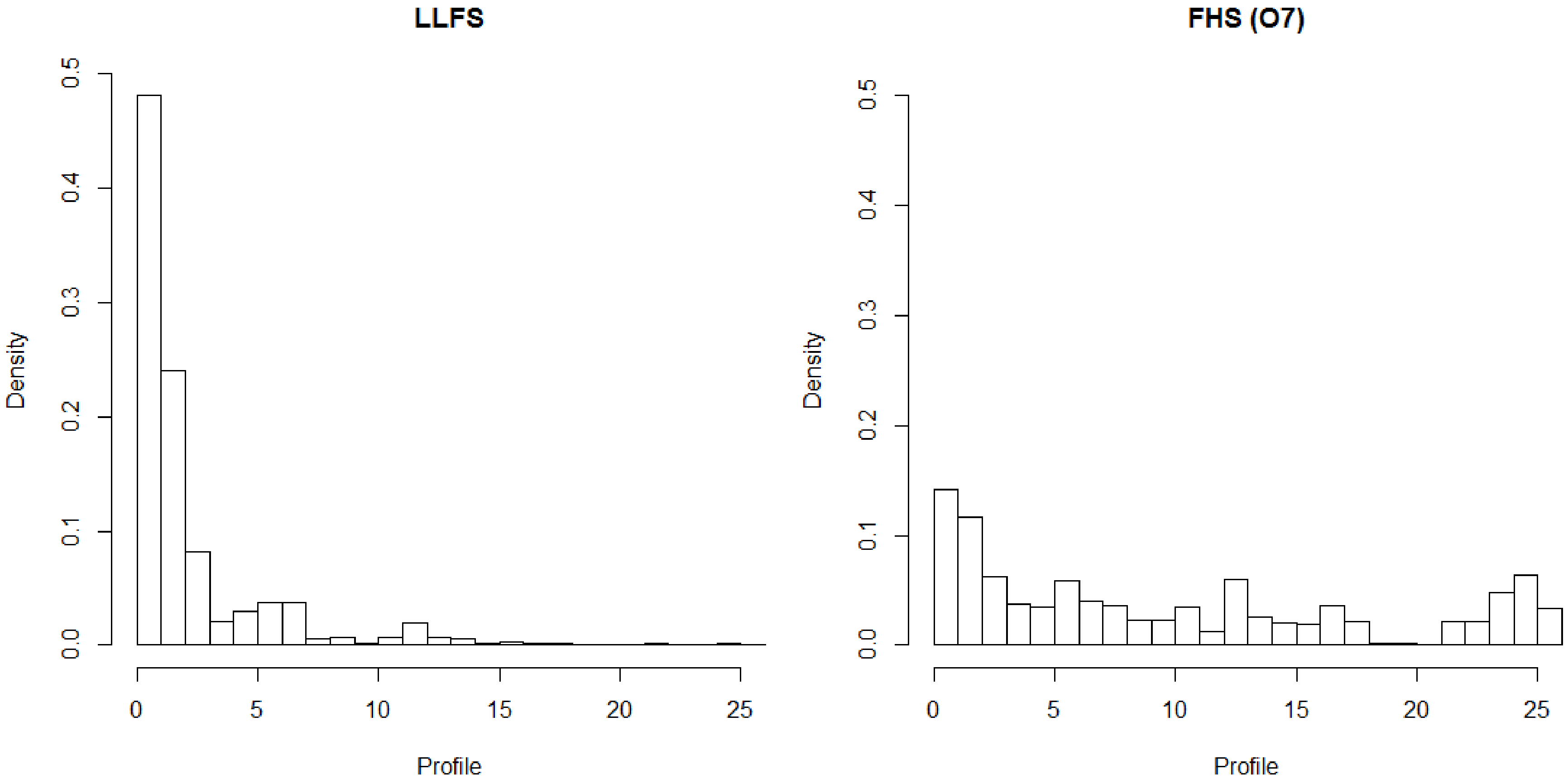

We see many more “anomalous patterns” in FHS

Figure S22-a Biomarker Signatures 1-4 in FHS

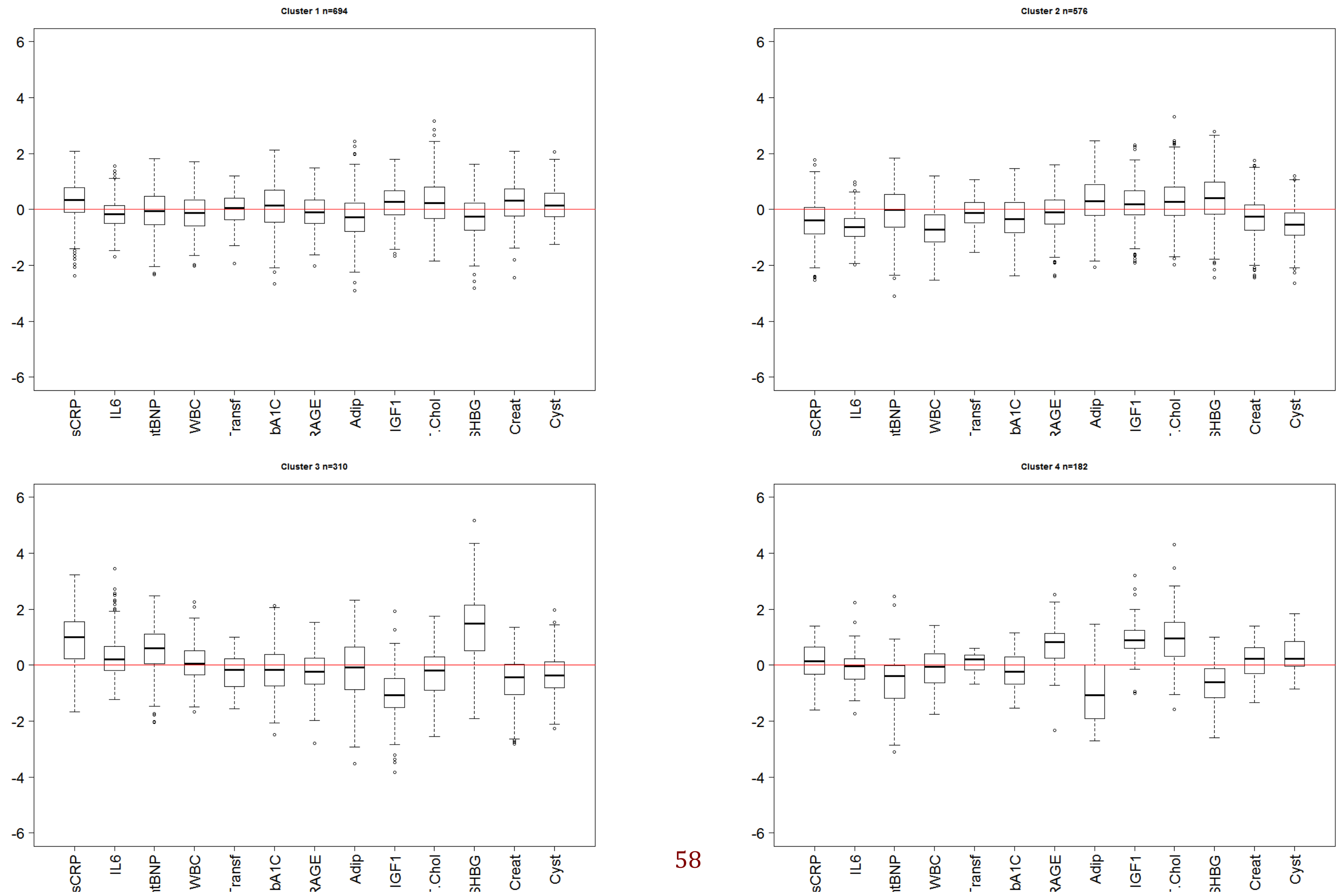

Figure S22-b Biomarker Signatures 5-8 in FHS

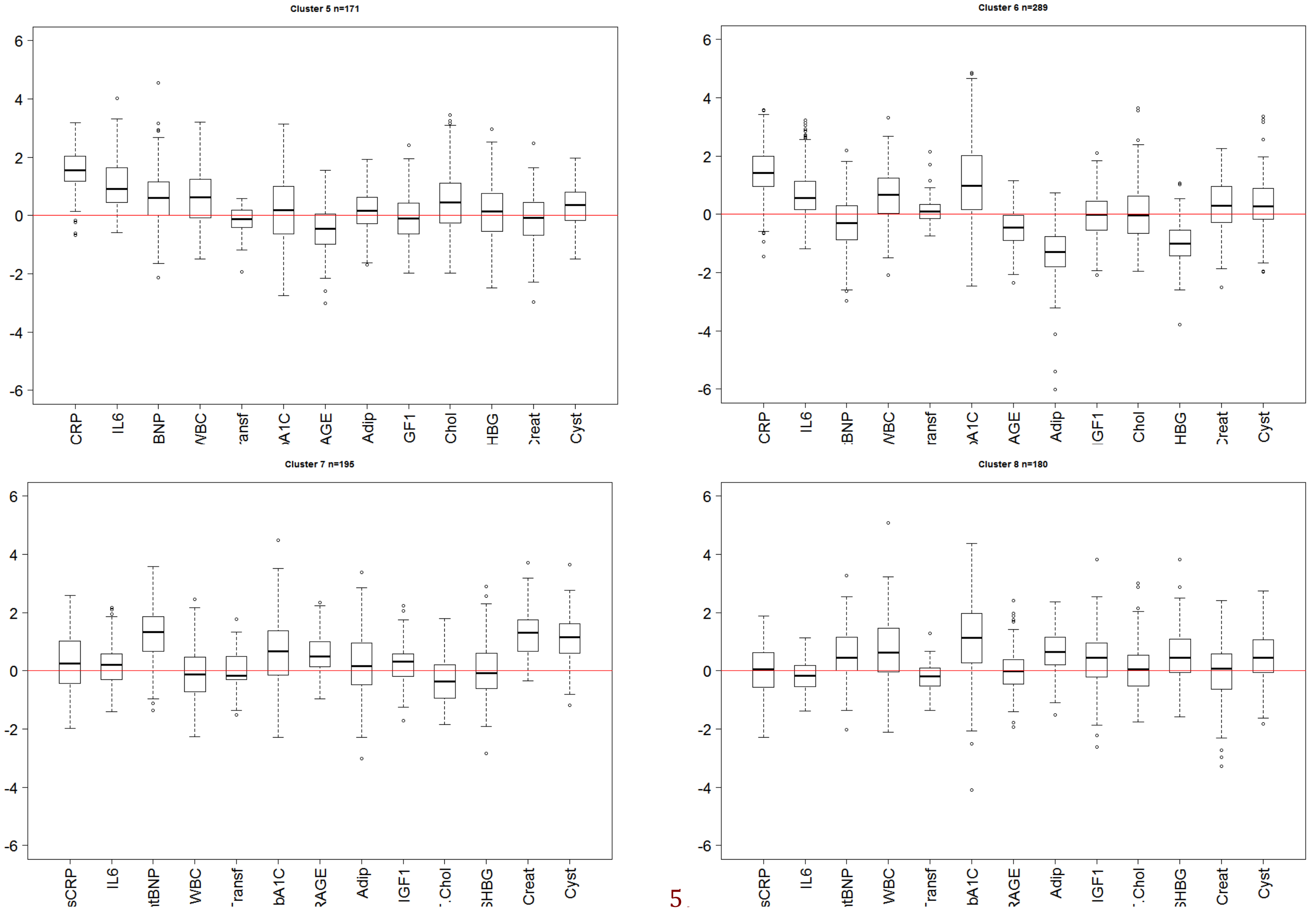

Figure S22-c Biomarker Signatures 9-12 in FHS

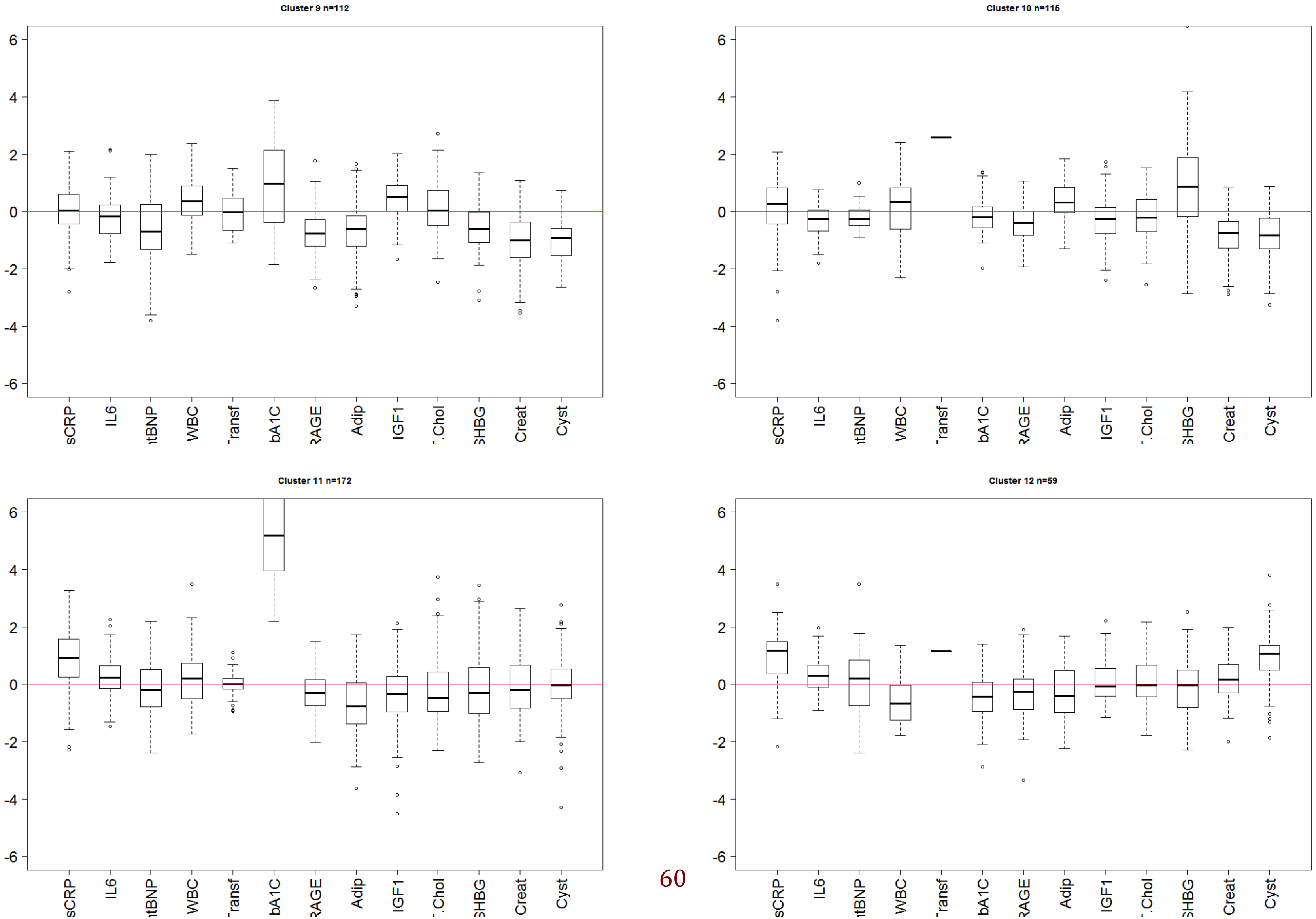

Figure S22-d Biomarker Signatures 13-16 in FHS

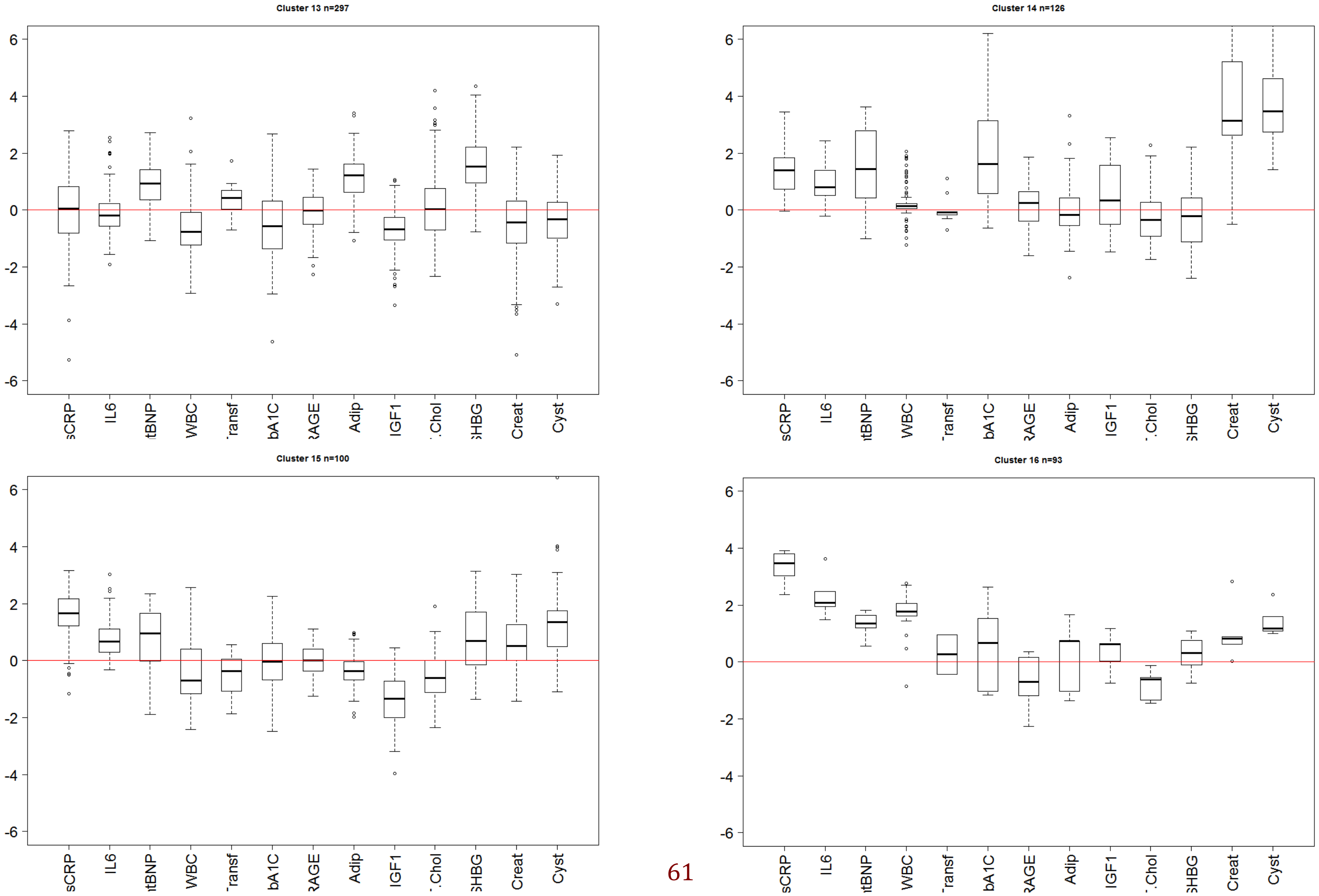

Figure S22-e Biomarker Signatures 17-20 in FHS

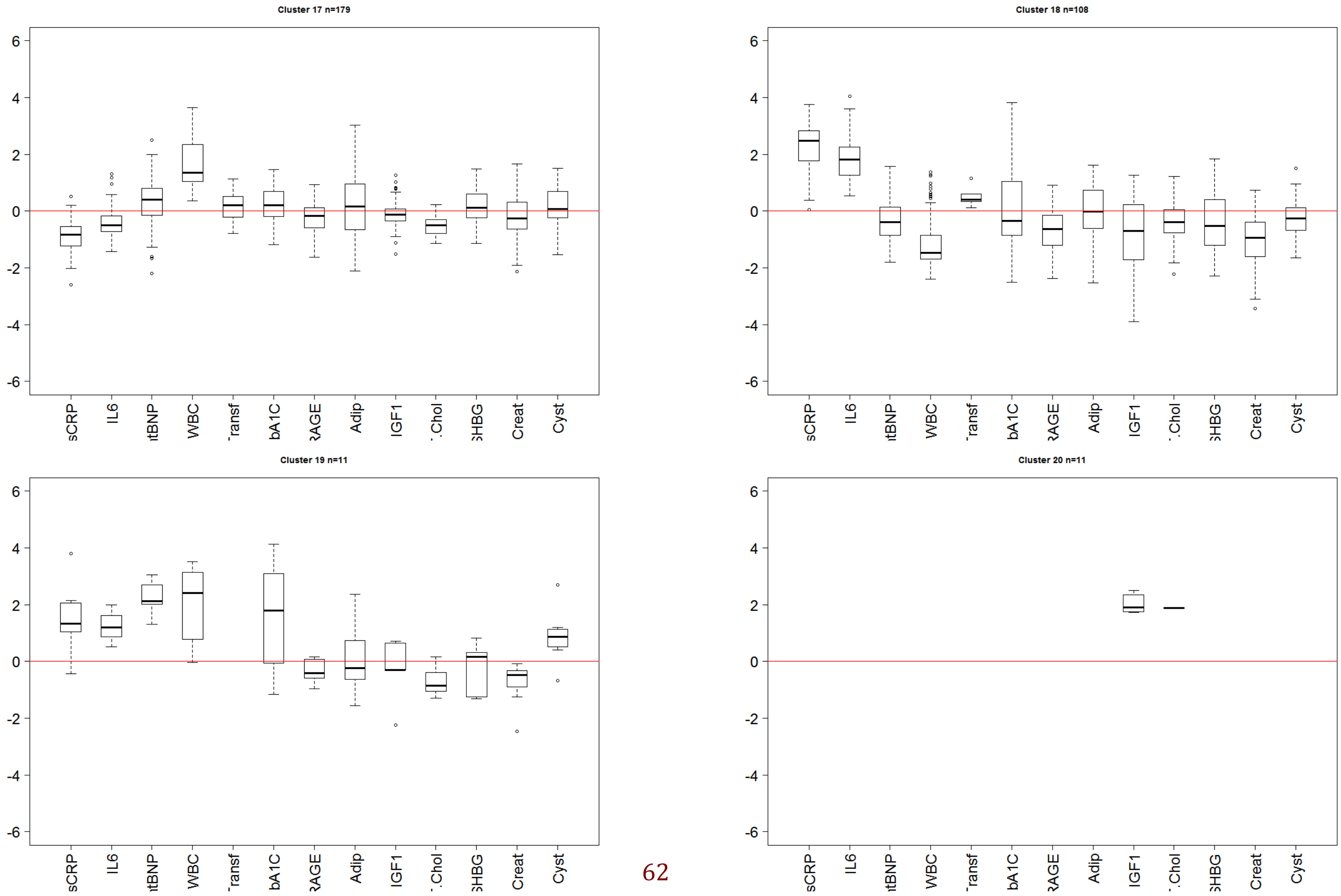

Figure S22-f Biomarker Signatures 21-24 in FHS

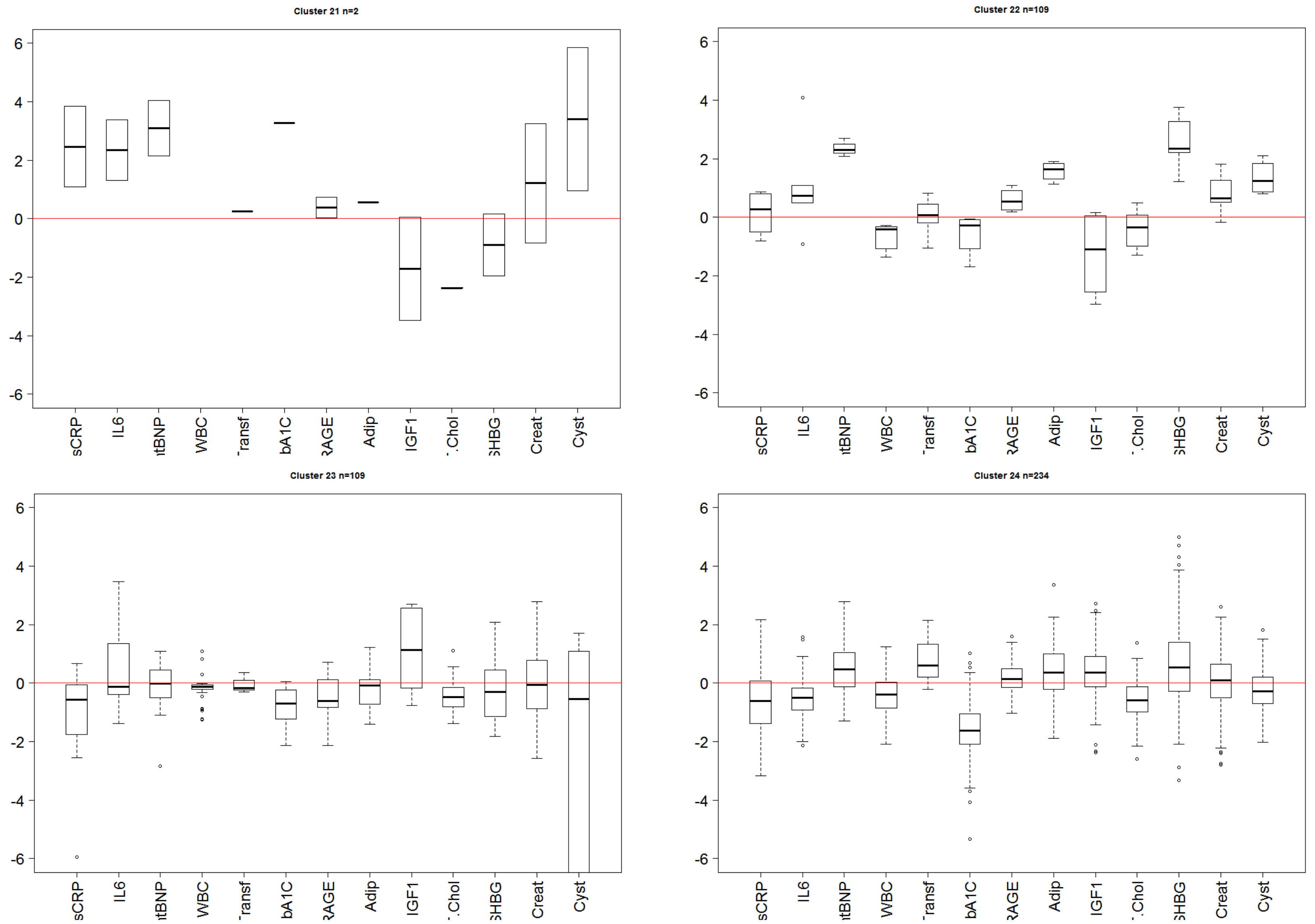

Figure S22-g Biomarker Signatures 25, 26 in FHS

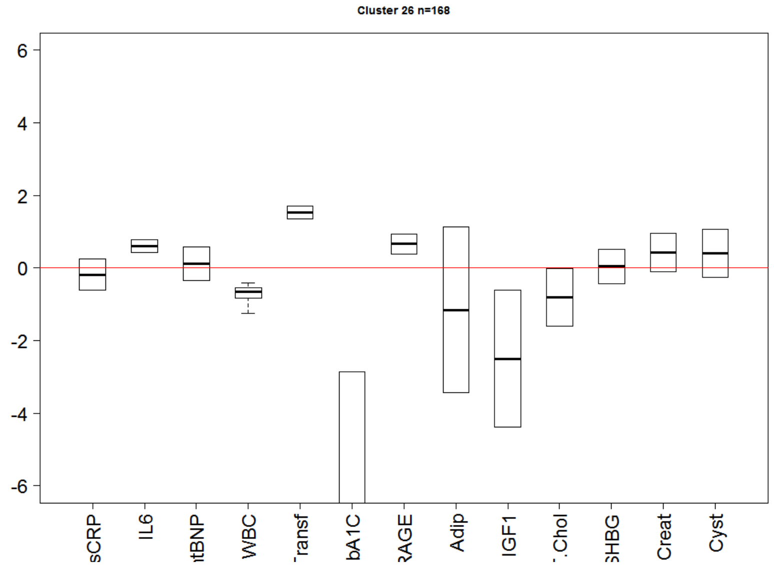

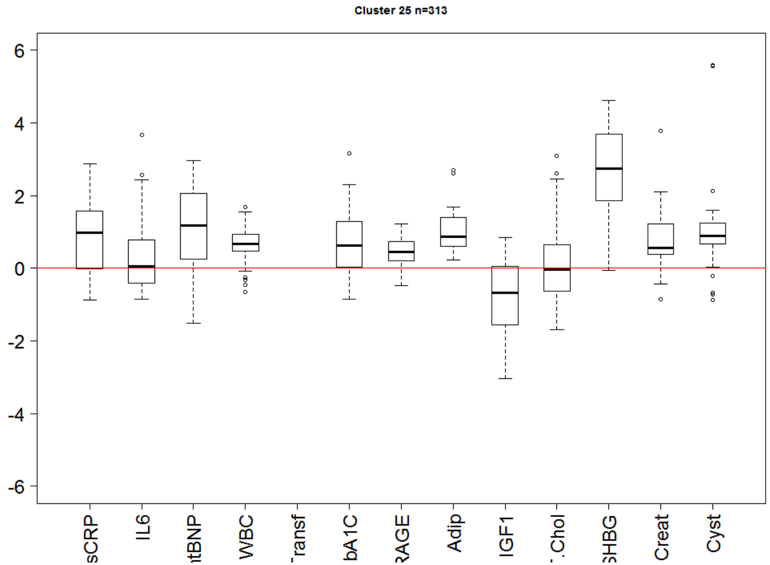

Supplement: Supplementary file 1 — Fig. S1 Flow chart of the analytic approach. Fig. S2 Distribution of age at enrollment in LLFS. Fig. S3 Overview of cluster analysis to discover biomarker signatures. Fig. S4 (a,b) Age and sex distribution of biomarkers. Fig. S5–S17 Description of 26 biomarker signatures in LLFS Fig. S18 Age and sex specific distribution of 19 biomarkers in clusters 1–17. Fig. S19 Example of lab‐bias in the measurement of albumin. Fig. S20 distribution of externally standardized biomarkers in FHS data using LLFS means and standard deviations. Fig. S21 Distribution of biomarker signatures in LLFS and FHS offspring. Fig. S22 Reproduced biomarker signatures in FHS offspring. [file ACEL-16-329-s001.pdf]
