# Supplementary material for: Biomarker signatures of aging
Source: Aging Cell. 2017 Jan 6;16(2):329–38. doi: 10.1111/acel.12557 (PMC5334528; doi:10.1111/acel.12557)
Supplement: Supplementary file 2 — Table S1 baseline characteristics of LLFS participants included in the analysis by generation and sex (mean and standard deviation) Table S2 (Clusters 1 to 13) The table displays the cluster number, the cluster size, and the cluster signature defined by mean and standard deviation of the standardized biomarkers Table S2 (Clusters 14 to 26) The table displays the cluster number, the cluster size, and the cluster signature defined by mean and standard deviation of the standardized biomarkers Table S3 Results of the ‘Leave‐one‐biomarker‐out’ replication Table S4 Summary demographics of patients allocated to the 26 clusters Table S5 Comparative analysis of 7 aging related phenotypes in LLFS Table S6 The table list the biomarkers available in the FHS by generation (g1: original; g2: offspring; g3: grand‐children), exam number, and age range and number of patients with available data Table S7 The table show the list of biomarkers in which there is a significant lab effect (column 2) and a significant cohort effect (column 3) Table S8 Sensitivity and misclassification rate of the Bayes Rule Table S9 Positive predicted values rate of the Bayes Rule Table S10 PPV rate of the Bayes rule with the subset of biomarkers available in the FHS generation 1 Table S11 PPV rate of the Bayes rule with subset of biomarkers in FHS offspring Table S12 Replication of association of signatures with mortality (FHS Cohort) Table S13 Replication of association of signatures with CVD risk (FHS Cohort) Table S14 Replication of association of signatures with Type 2 Diabetes (FHS Cohort) Appendix S1 Material and methods [file ACEL-16-329-s002.docx]

**Supplement Material and Methods**

**Study Populations:**

**Long Life Family Study (LLFS).** This is a family-based, longitudinal study of healthy aging and longevity that enrolled approximately 4,900 subjects in 583 families between 2006 and 2009 via three American and one Danish field centers. Potential probands were recruited based on older age, capacity to understand the study and their Family Longevity Selection Score (FLoSS). The FLoSS discriminates the degree of familial longevity using sex and birth-year cohort survival probabilities of the proband and their siblings ([1](#_ENREF_1)). Eligibility of sibships for the study was based on a FLoSS score >7 and having at least one living sibling and at least one offspring willing to be enrolled in the study. Socio-demographic, medical history data, current medical conditions and medications, physical and cognitive function data and blood samples were collected via in-person visits for all subjects at the time of enrollment as described elsewhere ([2](#_ENREF_2), [3](#_ENREF_3))**.** Participants are also followed up annually if aged 70 years and older, and every two years if younger, by telephone and mailings to obtain updated information about medications, hospitalizations and medical history. All surviving participants are currently undergoing a second in-person evaluation (taking place in 2015-2018). All participants underwent informed consent at the local institutions. Genetic, biomarker and phenotypic data are available from dbGaP (**dbGaP Study Accession:** phs000397.v1.p1). Characteristics of patients at the baseline visit are in **Supplement Table S1 and Supplement Figure 2**.

**The Framingham Heart Study (FHS).** The FHS is an on-going, family-based longitudinal study that was initiated in 1949 to identify risk factors of cardiovascular disease. The FHS evolved over the years to study the epidemiology and genetics of a wide spectrum of age-related diseases ([4](#_ENREF_4)). The original cohort enrolled more than 5,000 participants aged between 28 to 62 years old, and participants were examined every two years since 1949 for a total of 31 exams thus far, and data are available for the first 28 exams. The offspring cohort was recruited in 1971 and consisted of 5,124 offspring of the original cohort members and their spouses. Participants have been examined every 4 to 8 years, and data of the first 8 exams are available. The third generation consists of 4,095 children of offspring aged between 19 to 72 years old recruited in 2002, and data from 2 exams are available for this cohort. All participants underwent informed consent at Boston University IRB.

**Biomarkers in LLFS.** Fasting blood samples in LLFS participants were obtained following a standardized venipuncture protocol by staff at baseline visit. Approximately 50 mL of blood specimens were collected according to the standardized protocol ([2](#_ENREF_2)). The serum tubes were kept at room temperature for 30-45 minutes prior to centrifugation to allow for clotting and centrifuged on site at 3000 x g for 10 minutes. The centrifuged serum tubes along with the other unprocessed blood tubes were shipped to the Advanced Diagnostics and Research Laboratory (ARDL) at the University of Minnesota. To evaluate peripheral blood mononuclear cells, the anticoagulated EDTA tubes were centrifuged at 3000 x g for 30 minutes at 15°C. An unprocessed EDTA tube was used for measurement of complete blood counts. All serum, plasma aliquots and RNA samples were stored at -80°C until analysis. The central laboratory maintains a biorepository of plasma, serum, genomic DNA, and RNA for future analysis. Description of the assays and their accuracy is in ([5](#_ENREF_5)). The selection of biomarkers to assay was based on known or hypothetical association with aging-related diseases and include: complete blood count, lipids (triglycerides, HDL-c, LDL-c and total cholesterol), 25-OH vitamin D2 and D3, and vitamin D epi-isomer, diabetes related biomarkers (adiponectin, insulin, insulin-like growth factor 1 (IGF1), glucose, hemoglobin A1C, and soluble receptor for advanced glycation end-product (sRAGE)), kidney disease related biomarkers (albumin, creatinine, cystatin), sex hormone markers (dehydroepiandrosterone (DHEA), sex-hormone binding globulin (SHBG)) markers of inflammation (interleukin 6 (IL6), high-sensitive C-reactive protein, NT-proBNP), ferritin and transferrin.

**Biomarkers in FHS**. Protocols of biomarkers data in FHS are available from the study web site (<https://www.framinghamheartstudy.org/researchers/description-data/index.php>). To replicate signatures of biomarkers discovered in LLFS, data collected at exams 20 and 21 were used for the original FHS cohort, data at exam 7 for the offspring cohort, and data at exam 1 for the third generation (**Supplement Table S6**). WBC at exam 2 was used as proxy of the assay at exam 7 in the offspring generation.

**Data Preprocessing and Selection of Biomarkers.** Biomarkers with more than 5% missing data or Pearson squared correlation < 0.15 with age at enrollment were not included in the analysis. Each biomarker underwent stringent quality control that included imputation of values below the range of detection using uniform distributions between 0 and the lower detection level (DHEA, IGF1, hsCRP) and cubic root transformation or a log-transformation to symmetrize the distribution as needed. All other missing data were filled in using regression based imputation adjusted for age and sex (< 5% missing data assuming data missing at random). Appropriately transformed biomarkers data were standardized using age group and sex-specific means and standard deviations as shown below, where X denotes the data matrix of b biomarkers, and n LLFS participants.

Age was divided into ranges of 5 years for ages between 45 and 100, while ages below 45 years were grouped together, and ages above 100 years were grouped together. To eliminate the effect of unusually large or small values, trimmed means were used after removal of the 2.5% extreme values at each tail. Correlation between pairs of biomarkers was examined to prune groups of highly correlated biomarkers (r^2^ < 0.36). These preliminary steps left 19 biomarkers that were used for subsequent cluster analysis. (See **Table 1**, **Supplement Figure 3**).

**Cluster Analysis.** Agglomerative hierarchical clustering with complete linkage based on Euclidean distance was used to group profiles of standardized biomarkers in LLFS participants, and the vector of distances D_o_ used for the agglomerative procedure was displayed in the dendrogram in **Supplement Figure 3**. Typically, cluster detection would be based on cutting the dendrogram in **Supplement Figure 3** horizontally to identify subtrees. To select clusters with some level of statistical significance, we used a novel resampling procedure to generate a reference distribution for D_o_ under the null hypothesis of no clusters in the data ([6](#_ENREF_6)). This reference distribution, say D_e_, was generated by independently permuting the profiles of each biomarker and conducting hierarchical clustering on the permuted data set in which there should not be clusters. The resampling procedure was repeated 10 times, and the reference distribution D_e_ was calculated as the average of the distances used for hierarchical clustering in each set. The QQ-plot of the observed and expected distances was used to show whether there are clusters in the data, and significant clusters were detected by cutting the dendrogram at the 99.6 percentile height of the reference distribution that would unlikely to be seen in random data (**Supplement Figure 3, top right**). Sensitivity analysis was conducted to examine the robustness of selected clusters to varying significance levels (**Supplement Figure 3, bottom**).

**Graphical Display of Clusters and Demographic Characteristics of Patients by Clusters.** The distribution of biomarkers for LLFS participants allocated to each cluster was depicted with side-by-side boxplots (**Figure 1 reports a selection of clusters and Supplement Figures 5 through 17 report all 26 clusters)** and summarized by means and standard deviations of each biomarker (**Supplement Table S2**).

**“Leave-One-Biomarker-Out” Replication.** To verify the relevance of the 19 biomarkers to define clusters, the cluster analysis was also repeated by removing one biomarker at a time. In each of the 19 “leave-one-biomarker-out” derived datasets, we run hierarchical cluster analysis and select clusters as in the primary analysis. The concordance between the cluster labels assigned to each patient in the primary analysis and reduced analysis were compared using Spearman correlation of the cluster labels. The correlation coefficient ranged between 0.15 when hemoglobin was dropped from the data to 0.34 when cystatin C was dropped (**Supplement Table S3**). This analysis suggested that all biomarkers are important for the definition of signatures because dropping any of these biomarkers would lead to different signatures.

**Effect of Familiality**. The evaluate the possible effect of familiality in the set of clusters, we searched for families enriched by specific profiles. We tested the enrichment of each of the ~550 LLFS families by profiles in the same signature, for each of the signature with more than 40 profiles. We limited attention to families with 10 or more family members. Fisher exact test was used to test the hypothesis of no-enrichment by comparing the observed number of profiles of the same signature versus the number of profiles expected by a random selection. Levels of significance were corrected for multiple testing. Only one family appeared to be significantly enriched of profiles from signature 6 (3 in 17 family members were assigned to cluster 6, p-value 2 E-5). This result suggests that familiality did not drive the results of clustering.

**Annotation of Clusters.** **Supplement Table S4** describes summary demographics of the patients by clusters. The set of 26 clusters derived with this analysis were annotated by their ability to predict morbidity and mortality for events recorded in the longitudinal follow up, and by the association with longitudinally measured physiological markers (gait speed, grip strength, FEV1, digital symbol substitution test (DSST) and mini mental state exam (MMS), BMI, pulse rate and systolic blood pressure). Cox proportional hazard models stratified by sex and adjusted by age at enrollment were used for the analyses of incident events. For each phenotype, ages were censored at last contact or at death as necessary. CVD was defined as myocardial infarction, coronary artery bypass grafting, atrial fibrillation, congestive heart failure or valve replacement (**Table 2**). Age and sex adjusted mixed effect linear models for repeated measures were used to estimate differences between physiological markers associated with the clusters (**Fig. 3 and Supplement Table S5**). To test the hypothesis that rates of change in physiological markers differed by clusters, interaction terms between age and cluster memberships were included in the models and the significance was tested using Markov Chain Monte Carlo methods.

**Validation of the signatures’ predictive values in the FHS.** We searched the FHS database to identify the exams with the largest number of the 19 biomarkers available in each generation and identified 8 biomarkers measured between exam 20 and 21 in the original generation (n=1537, years of blood collection 1988-1992), 13 biomarkers measured at exam 7 of the offspring generation (n=4915, years of blood collection 1998-2002), and 10 biomarkers measured at exam 1 of the third generation (n=4592, years of blood collection 2002-2005). WBC data measured at exam 2 for the offspring generation were used as a proxy of the WBC data at exam 7, since this marker changes slowly with ages <75 (**Supplement Figure 4b**), and more than 90% of the offspring generation at exam 7 was less than 75 years of age. Details of the available biomarkers in each FHS generation are in **Supplement Table S6**. Because we did not find the complete set of 19 biomarkers, we could not try replicating the analysis to discover signatures of biomarkers and focused on comparing the age and sex distributions of biomarkers in FHS relative to LLFS, and on validation of the predictive value of the biomarkers signatures.

**Calibration of biomarkers.** Visual inspection of the distribution of biomarkers in LLFS and FHS suggested lab-to-lab bias that could confound the analysis (see example of albumin in **Supplement Figure 19** and the systematic difference between measurements for each age group and sex). To remove lab-to-lab bias and calibrate the data, we aggregated biomarker data from LLFS and FHS, and each biomarker was analyzed using a multivariable regression model with indicators for cohort type (LLFS or FHS), age groups (<45 years, 45-49,…,>100), and interaction terms between cohort type and each age group. Significant cohort effect was taken to indicate a lab specific bias, and significant interaction terms for one or more age groups were taken to denote a cohort specific effect that represents a difference in biomarker distribution between LLFS and FHS after the lab-bias is taken into account. The analysis was conducted for males and females separately, using appropriately transformed biomarkers data in FHS as in LLFS.

To generate age and sex “externally standardized biomarker data” in FHS, we subtracted the estimated FHS cohort effect from the biomarker data measured in FHS, and standardized the data using age and sex specific means and standard deviations computed in the LLFS. Specifically, the following statistical model was used to estimate lab-specific effect, and cohort specific age effect.

$$\begin{matrix} y_{age,sex}=\text{biomarker (log-transformed or cubic-root transformed if needed)} \\ \text{E(y}_{\text{age,sex}}\text{)}=\alpha_{\text{0,sex}}+\sum_{FHS\_g} \alpha_{FHS\_g,sex}+\sum_{age\_g} \alpha_{age\_g,sex}+\text{ }\sum_{FHS\_gxage\_g} \alpha_{FHS\_gxage\_g,sex} \\ \alpha_{\text{0,sex}}=mean(y)\text{ in LLFS, age group }<50 \\ \alpha_{FHS\_g,sex}=\text{ sex specific FHS cohort effects (g}=0=\text{original; g}=\text{1 offspring; g}=\text{3; gen3)} \\ \alpha_{age\_g,sex}=\text{sex specific age group effect} \\ \alpha_{FHS\_gxage\_g,sex}=\text{sex specific age group x cohort interaction} \\ \\ \text{Cohort effect: some }\alpha_{FHS\_gxage\_g,sex}\text{ }\neq\text{ 0} \\ \text{Lab effect: }\alpha_{FHS\_g,sex}\neq0\text{ } \end{matrix}$$

The analysis was conducted for each biomarker, in each FHS cohort and separately for males and females. From the regression analysis, we estimated the FHS cohort effect and standardized the biomarker data in FHS cohort using age and sex specific mean and standard deviation from the LLFS data using the formula:

Data from FHS participants enrolled in the 3rd generation showed some age related bias, probably due to the age gap, compared to LLFS cohort (median age at enrollment 40 years, interquartile difference IQ=34;46 years). This group, therefore, was not included in the validation analysis.

**Replication of associations between biomarker signatures and morbidity and mortality in FHS**. We built and validated a 26-label Bayesian classifier that computes the probability of an individual to have a profile of biomarkers matching the 26 signatures discovered in LLFS. The Bayesian classifier uses the formula

$p(c_{j}|x_{1}^{*},x_{2}^{*},...,x_{19}^{*})\propto\prod_{i=1}^{19} f(x_{i}^{*}|c_{j})p(c_{j})$ (1)

to compute the probability that a subject’s biomarker profile $x_{1}^{*},x_{2}^{*},...,x_{19}^{*}$ matches the signature associated with the jth cluster (say $c_{j}$), and selects the signature associated with the maximum probability ([7](#_ENREF_7)). In the formula, $f(x_{i}^{*}|c_{j})$ is the density function of a normally distributed variable with mean and standard deviation estimated from the data of the ith biomarkers in cluster $c_{j}$, and $p(c_{j})$is the prior probability that $c_{j}$is the true signature. Means and standard deviations of the 19 biomarkers for each of the 26 clusters discovered in LLFS are in **Supplement Table S2**. The advantages of this classification rule is that it can work with incomplete data, it is equivalent to logistic regression when there are only two classes ([8](#_ENREF_8)), and it has proved to be highly effective notwithstanding the simplifying assumptions ([9](#_ENREF_9)). The Bayesian classifier was used in the LLFS data to assess the goodness of fit of the rule and to estimate the proportion of positive predicted values and the misclassification error (**Supplement Tables S8 and S9**). The analyses were also repeated using the subset of biomarkers available in the FHS, to see how these measures of accuracy are affected when only a subset of biomarkers is available. The results are described in **Supplement Tables S10 and S11**.

The classifier was then used to identify the most likely biomarker signature of FHS participants using the externally standardized biomarker data. In all cases uniform prior probabilities were used.

**Validation of Predictive Value in FHS**. Once each FHS participant was assigned the most likely signature from the set of 26 using the Bayesian classification rule, incident risk for mortality and morbidity associated with each biomarker signatures were compared as in LLFS, using subjects assigned signature 1 as referent group. Prospectively collected data after exam 7 for offspring, and exam 20 for the original generation were used. CVD was defined as myocardial infarction, coronary insufficiency and congestive heart failure. Cox proportional hazard regression models were adjusted for age at blood collection and stratified by sex. Complete results of these analyses are in **Supplement Tables S12, S13 and S14**.

All analyses were conducted in the statistical program R v3.

1. Sebastiani P, Hadley EC, Province M, Christensen K, Rossi W, Perls TT, et al. A family longevity selection score: ranking sibships by their longevity, size, and availability for study. Am J Epidemiol. 2009;170(12):1555-62. PMCID: 2800272.

2. Newman AB, Glynn NW, Taylor CA, Sebastiani P, Perls TT, Mayeux R, et al. Health and function of participants in the Long Life Family Study: A comparison with other cohorts. Aging (Albany NY). 2011;3(1):63-76. PMCID: 3047140.

3. Sebastiani P, Sun FX, Andersen SL, Lee JH, Wojczynski MK, Sanders JL, et al. Families Enriched for Exceptional Longevity also have Increased Health-Span: Findings from the Long Life Family Study. Front Public Health. 2013;1:38. PMCID: PMC3859985.

4. Tsao CW, Vasan RS. Cohort Profile: The Framingham Heart Study (FHS): overview of milestones in cardiovascular epidemiology. Int J Epidem. 2015;44(6):1800-13.

5. Sebastiani P, Thyagarajan B, Sun F, Honig LS, Schupf N, Cosentino S, et al. Age and Sex Distributions of Age-Related Biomarker Values in Healthy Older Adults from the Long Life Family Study. J Am Geriatr Soc. 2016.

6. Sebastiani P, Perls TT. Detection of significant groups in hierarchical clustering. Submitted2016.

7. Domingos P, Pazzani M. On the Optimality of the Simple Bayesian Classifier under Zero-One Loss. J Mach Learn. 1997;29(2):103-30.

8. Sebastiani P, Solovieff N, Sun JX. Naive Bayesian Classifier and Genetic Risk Score for Genetic Risk Prediction of a Categorical Trait: Not so Different after all! Front Genet. 2012;3:26. PMCID: 3289795.

9. Hand DJ, Yu K. Idiot's Bayes: Not So Stupid after All? Intern Statist Review 2001;69(3):385-98.


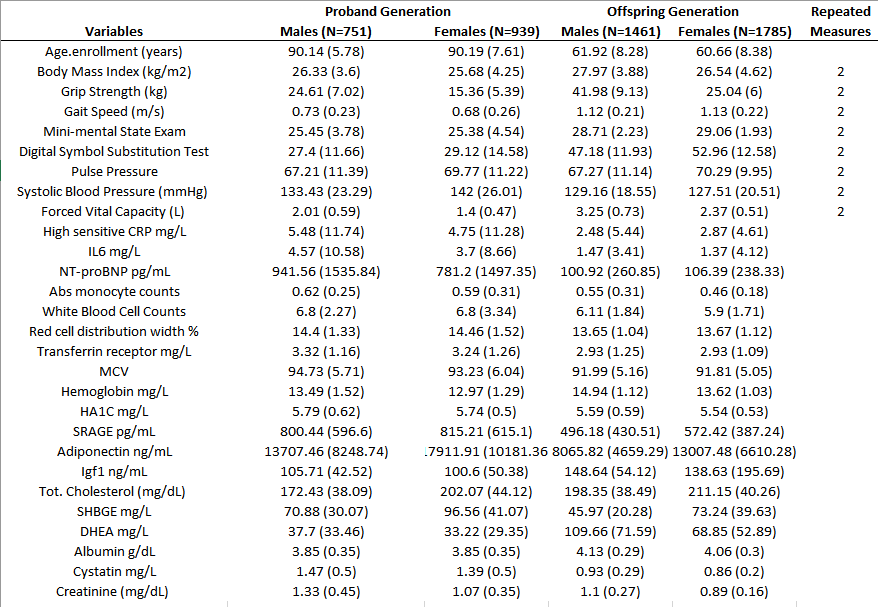


**Table S1**: baseline characteristics of LLFS participants included in the analysis by generation and sex (mean and standard deviation).


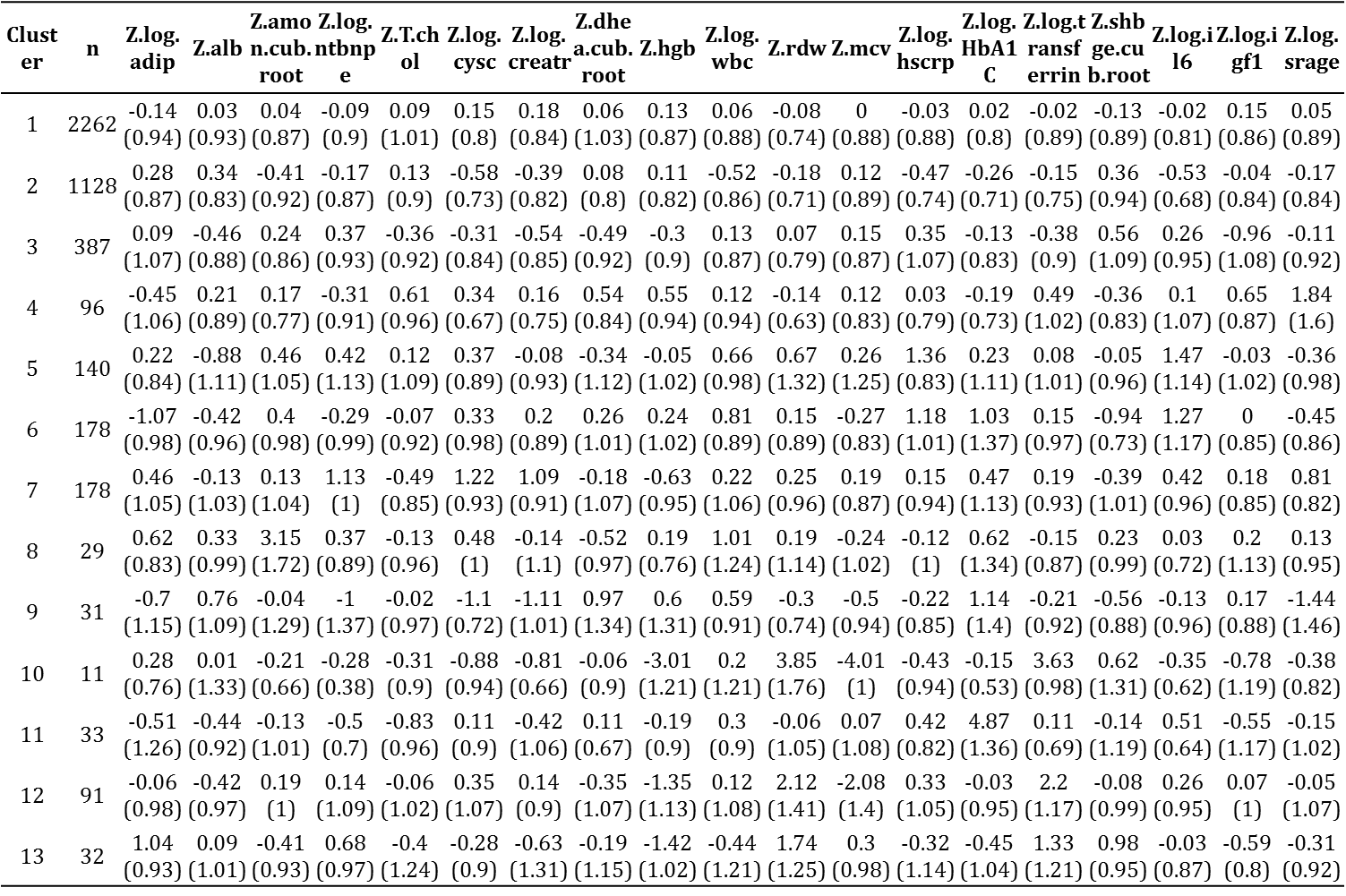


**Table S2 (Clusters 1 to 13)** The table displays the cluster number, the cluster size, and the cluster signature defined by mean and standard deviation of the standardized biomarkers. Notation: Z.log.adip: adiponectin (log-transformed data); Z.alb: albumin; Z.amon.cub.root: absolute monocite counts (cubic root transformed); Z.log.ntbnpe: NT-proBNP (log-transformed data); Z.T.chol: Total cholesterol; Z.log.cysc: Cystatin (log-transformed); Z.log.creatr: Creatinine (log-transformed); Z.dhea.cub.root : DHEA (cubic root transormated); Z.hgb: hemoglobin; Z.log.wbc: white blood cell conts (log-transformed); Z.rdw: Red cell distribution width; Z.mcv: MCV; Z.log.hscrp: hsCRP; Z.log.HbA1C: HBA1C (log-transformed); Z.log.transferrin: Transferrin (log-trasnformed); Z.shbge.cub.root: SHBGE (cubic root transformed); Z.log.il6: IL6 (log-transformed); Z.log.igf1: IGF1 (log-trasnformed); Z.log.srage: sRAGE (log-transformed).


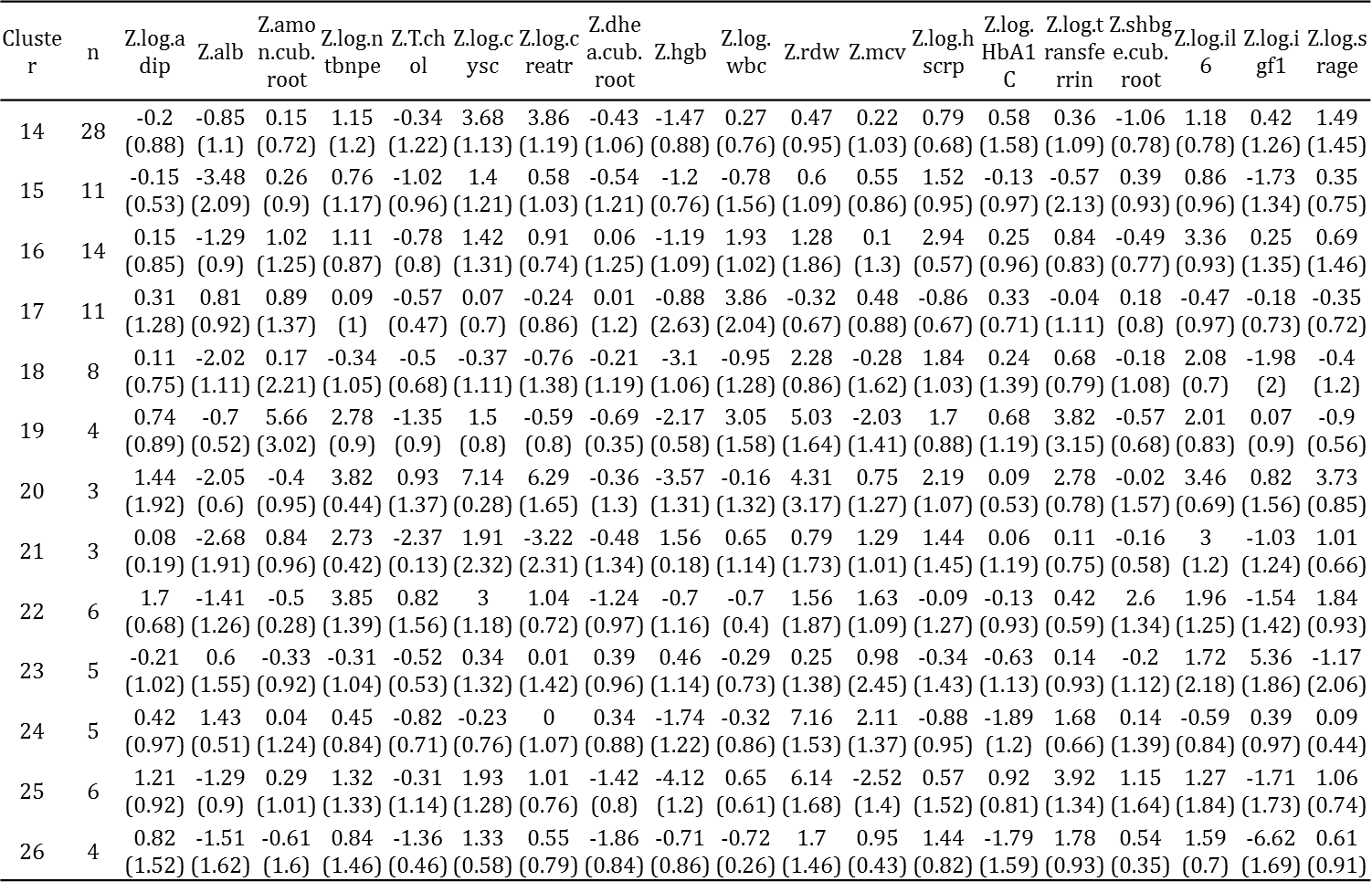


**Table S2 (Clusters 14 to 26)** The table displays the cluster number, the cluster size, and the cluster signature defined by mean and standard deviation of the standardized biomarkers. Notation: Z.log.adip: adiponectin (log-transformed data); Z.alb: albumin; Z.amon.cub.root: absolute monocite counts (cubic root transformed); Z.log.ntbnpe: NT-proBNP (log-transformed data); Z.T.chol: Total cholesterol; Z.log.cysc: Cystatin (log-transformed); Z.log.creatr: Creatinine (log-transformed); Z.dhea.cub.root : DHEA (cubic root transormated); Z.hgb: hemoglobin; Z.log.wbc: white blood cell conts (log-transformed); Z.rdw: Red cell distribution width; Z.mcv: MCV; Z.log.hscrp: hsCRP; Z.log.HbA1C: HBA1C (log-transformed); Z.log.transferrin: Transferrin (log-trasnformed); Z.shbge.cub.root: SHBGE (cubic root transformed); Z.log.il6: IL6 (log-transformed); Z.log.igf1: IGF1 (log-trasnformed); Z.log.srage: sRAGE (log-transformed).


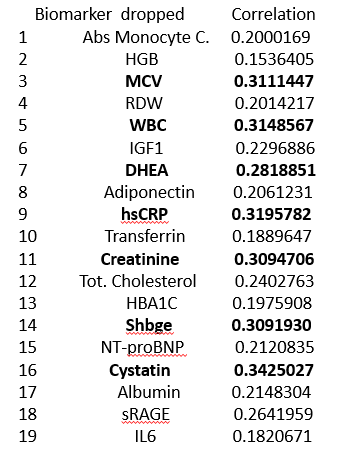


**Supplement Table S3. Results of the “Leave-one-biomarker-out” replication**. The table displays the correlation between cluster labels based on 19 biomarkers and 18 biomarkers, after leaving out one biomarker at a time. For example, 0.20 in row 1 is the correlation between the cluster labels based on the 19 biomarkers and the cluster labels based on 18 biomarkers when Abs monocyte counts was dropped from the analysis. Note that the higher the correlation, the lower the necessity of the biomarker to reproduce the same 26 clusters. The most important biomarker is HgB, with correlation 0.15, while the least important biomarker is cystatin, with correlation 0.34. Since all correlation values are relatively small, all 19 biomarkers are critical to derive the 26 signatures. We emphasize that while this analysis does not show that this 19-biomarker analysis produced the “best” signature, it shows that all biomarkers are necessary to generate the 26 clusters. With more biomarkers we would expect the signature to become even more specific.


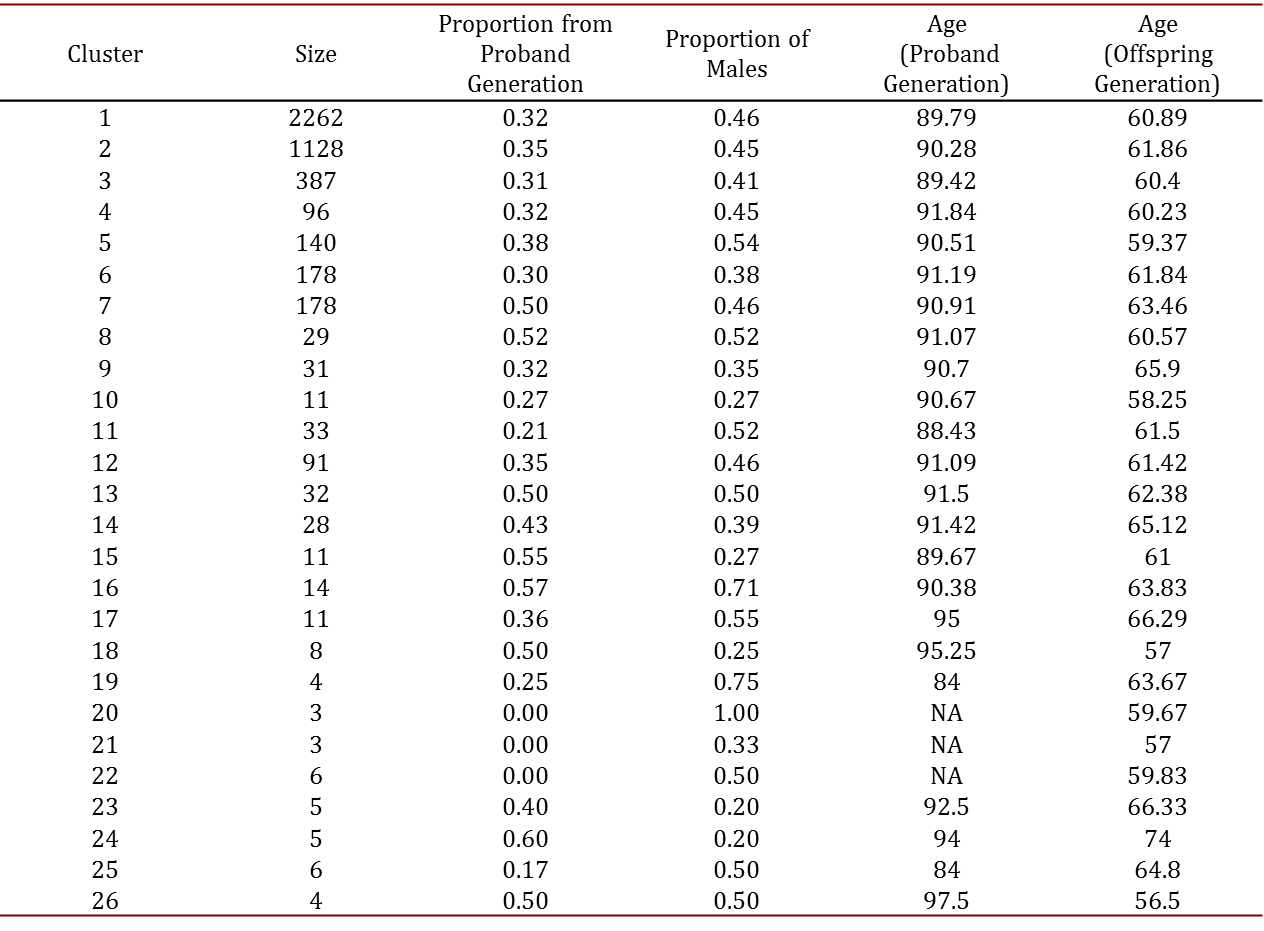


**Supplement Table S4.** Summary demographics of patients allocated to the 26 clusters.


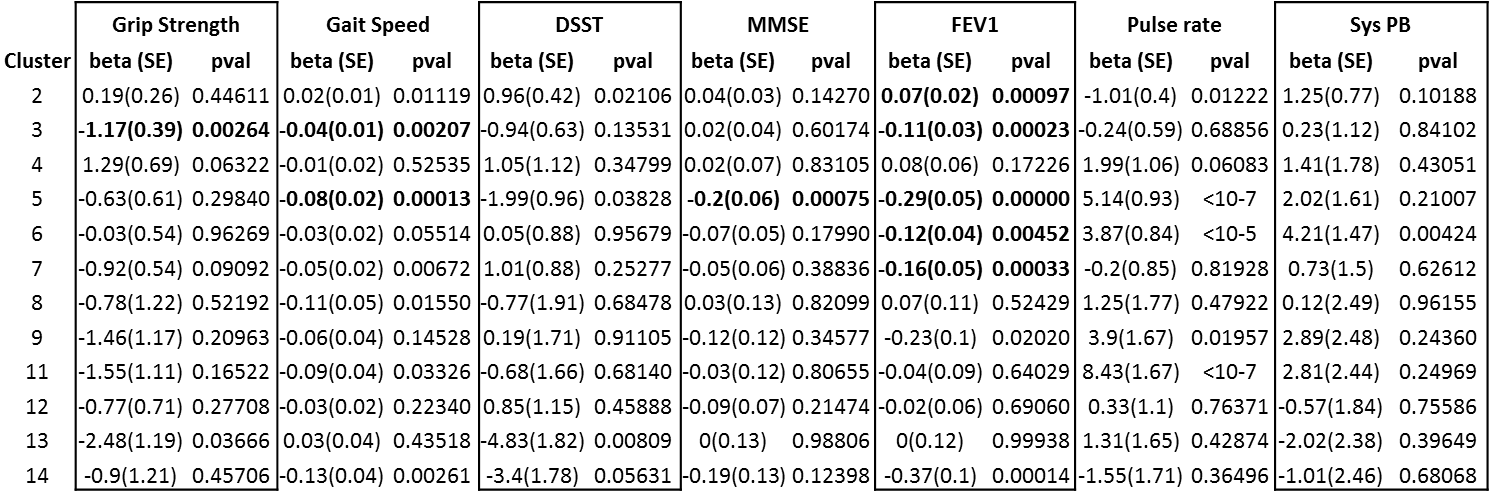


**Supplement Table S5**. Comparative analysis of 7 aging related phenotypes in LLFS. For each variable in the columns, the beta coefficient represents the estimated difference in variable comparing the cluster in the first column and the referent cluster. For example, 0.19 represents the estimated difference in grip strength between individuals in cluster 2 versus individuals in the referent cluster. SE is the standard error of the estimate and pval is the p-value to test the null hypothesis that the beta coefficient is 0. Highlighted in bold are the differences that remain significant using Bonferroni correction.


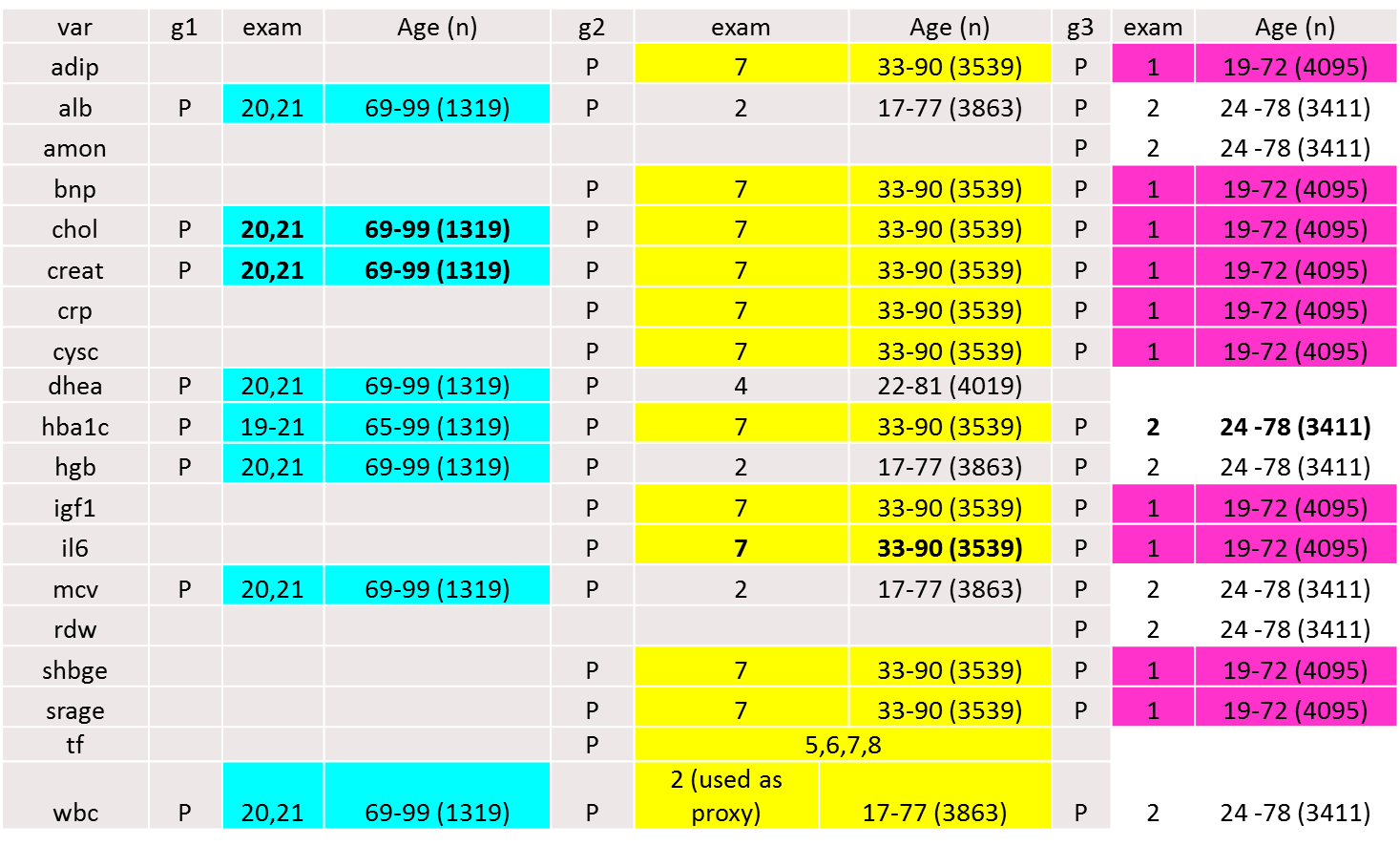


**Supplement Table S6.** The table list the biomarkers available in the FHS by generation (g1: original; g2: offspring; g3: grand-children), exam number, and age range and number of patients with available data.

**
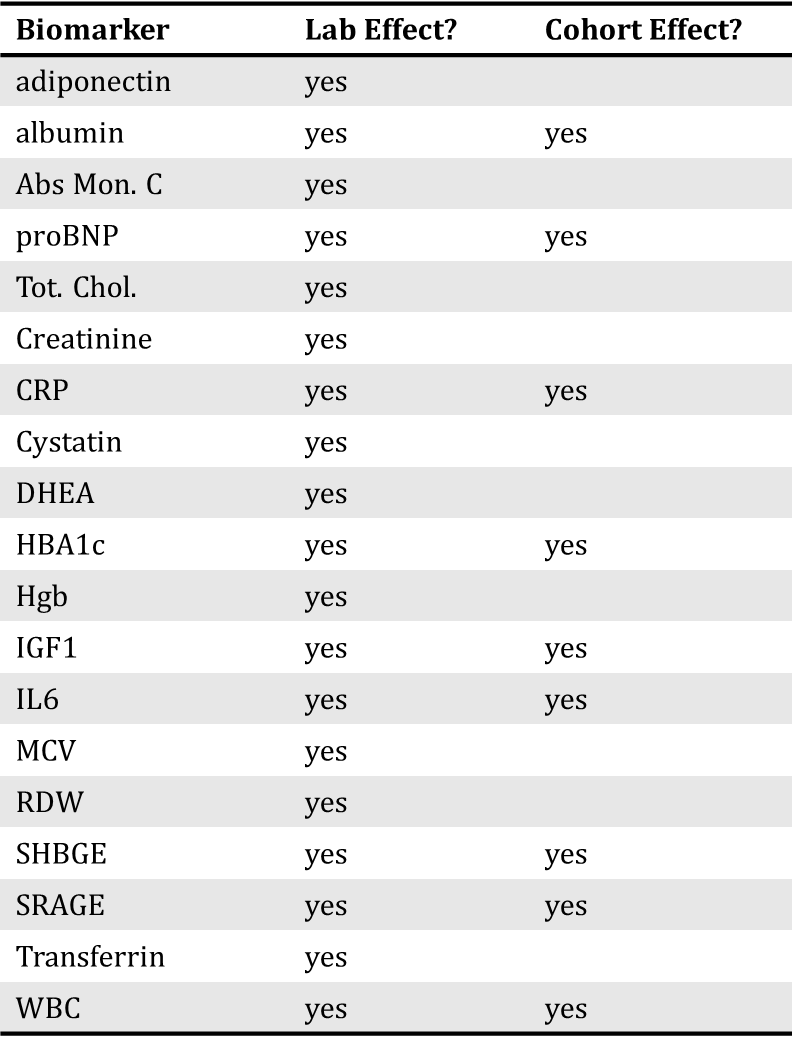
**

**Supplement Table S7.** The table show the list of biomarkers in which there is a significant lab effect (column 2) and a significant cohort effect (column 3)


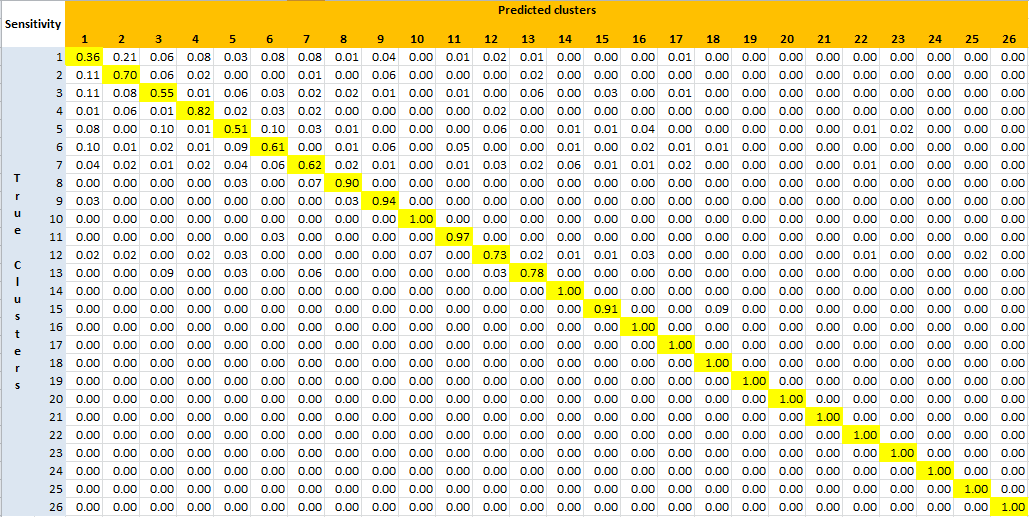


**Supplement Table S8. Sensitivity and Misclassification Rate of the Bayes Rule.** To assess the accuracy, we trained the classifiers using LLFS data and used it to estimate the probability of cluster membership in LLFS. We assigned the cluster label based on maximum posterior probability rule (this is label in the columns=predicted cluster). Random assignment: 1/26=3%. The diagonal elements in yellow show the sensitivity that ranges between 36% to 100%.High numbered clusters are more accurately predicted


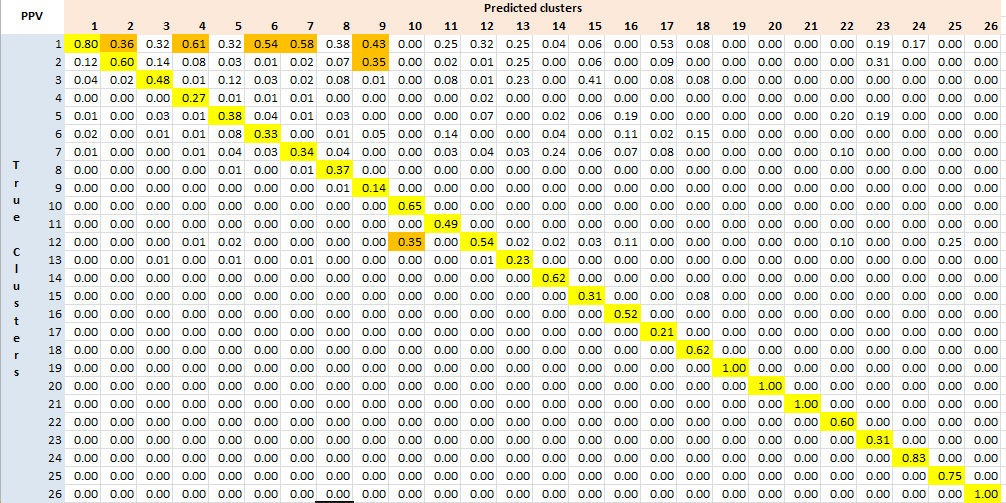


**Supplement Table S9. Positive Predicted Values Rate of the Bayes Rule.** Positive predicted value rate is the proportion of subjects assigned in cluster j by the Bayes rule that are actually in cluster j. The diagonal elements in yellow show the PPV that is above 50% in 14 of 26 clusters, and above 30% in 22 of 26. Based on this analysis, clusters predicted as 9 and 17 are less reliable than others.


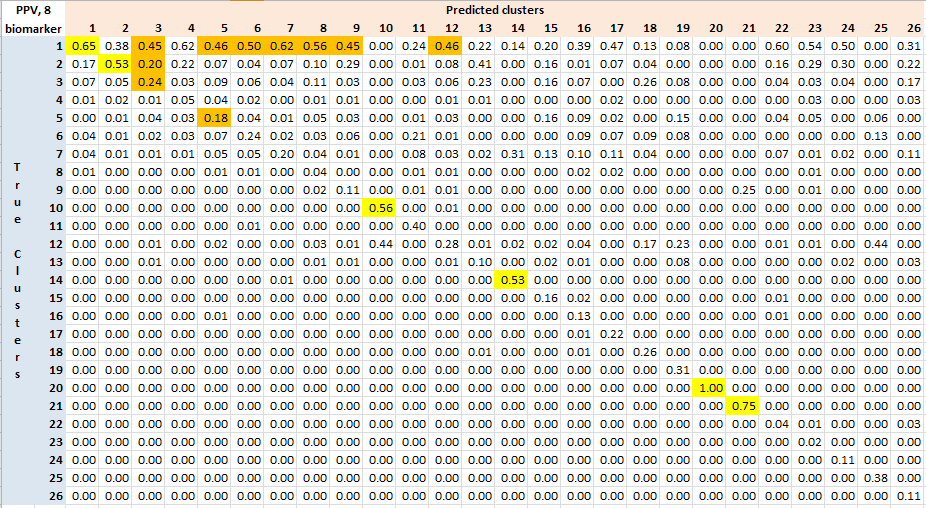


**Supplement Table S10. PPV Rate of the Bayes Rule With the Subset of Biomarkers available in the FHS Generation 1.** Positive predicted value rate using the Bayes rule with biomarkers albumin, total cholesterol, creatinine, DHEA, HbA1C, hemoglobin, MCV and WBC that would be available in FHS original cohort. Based on this analysis, cluster predicted as 2, 10,14 are reliable.


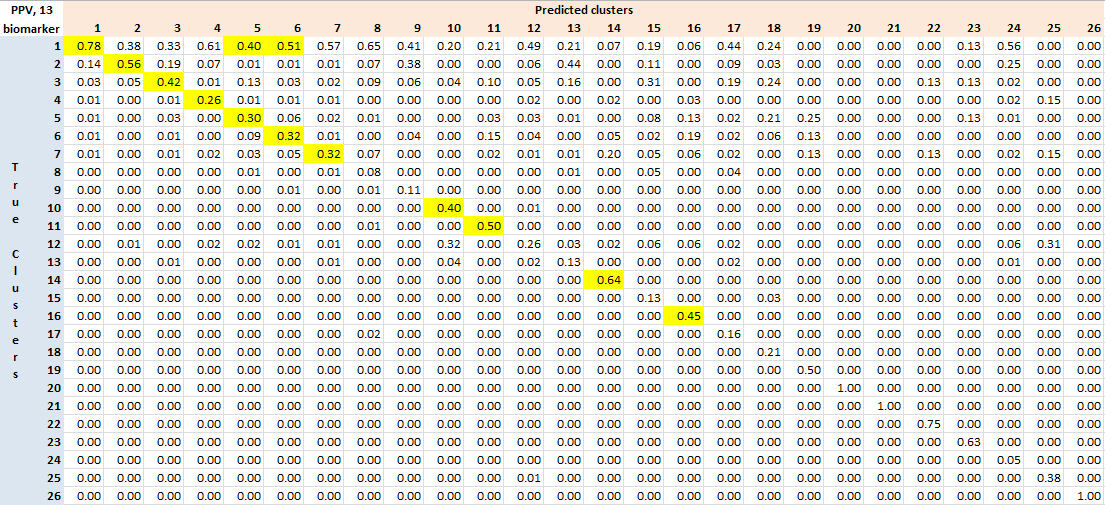


**Supplement Table S11. PPV Rate of the Bayes Rule With Subset of Biomarkers in FHS Offspring.** Positive predicted value rate using the Bayes rule with biomarkers adiponectin, NT-proBNP, Tot. Cholesterol, creatinine, cystatin, hsCRP, HBA1C, IGF1, IL6, SHBG, sRAGE, transferrin and WBC that would be available in FHS offspring cohort. Note that all biomarkers were measured at exam 7 while WBC was measured at exam 2.


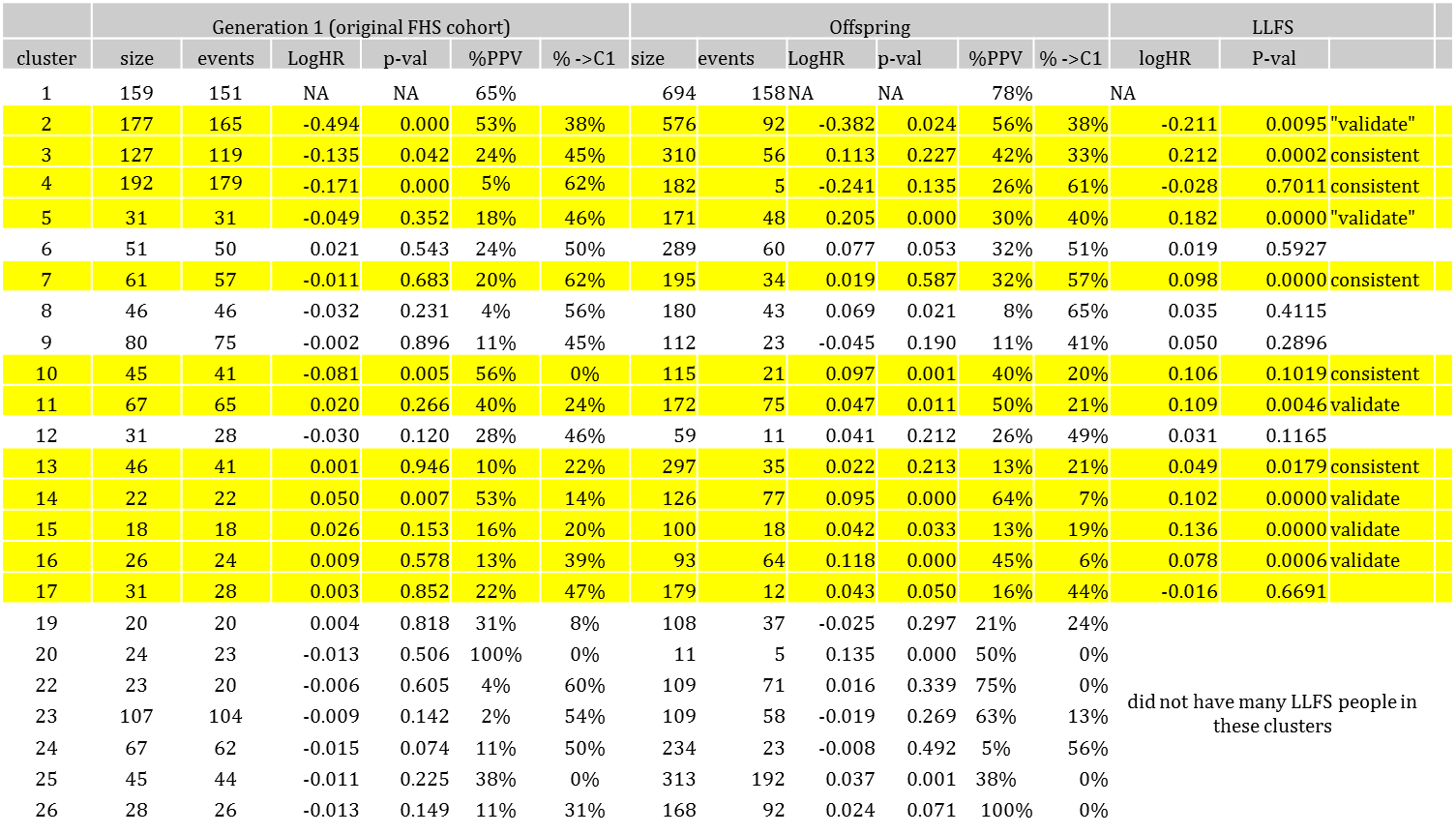


**Supplement Table S12. Replication of Association of Signatures with Mortality (FHS Cohort).** The table shows complete details of the validation of the risk for mortality relative to the referent group (cluster 1) in the offspring generation of the FHS. Log-hazard ratios (LogHR) were estimated using Cox proportional hazard regression, stratified by sex and adjusted by age at blood collection. Significance is based on p-values from Wald’s test. %PPV denotes the proportion of positive predicted values of each signature selected by the Bayesian classifier trained in the LLFS data. The column denoted by “%->C1” denotes the proportion of profiles assigned to the referent group.

|  | G0 (original generation in FHS) | | | | | Offspring Generation | | | | | LLFS | | | | |  |  | |
| --- | --- | --- | --- | --- | --- | --- | --- | --- | --- | --- | --- | --- | --- | --- | --- | --- | --- | --- |
| cluster | size | events | LHR | StDev | p-val | size | events | LHR | StDev | p-val | size | events | LHR | StDev | p-val |  |  |
| 1 | 159 | 85 | NA | 0 |  | 694 | 158 |  | 0 |  | 2262 | 151 |  | 0 |  |  |  |
| 2 | 177 | 87 | -0.374 | 0.032 | 0.035 | 576 | 92 | -0.382 | 0.029 | 0.024 | 1128 | 82 | -0.06 | 0.02 | 0.68 | consistent | |
| 3 | 127 | 72 | -0.109 | 0.008 | 0.221 | 310 | 56 | 0.113 | 0.009 | 0.227 | 387 | 25 | 0.08 | 0.01 | 0.48 |  |  |
| 4 | 192 | 102 | -0.121 | 0.003 | 0.028 | 182 | 5 | -0.241 | 0.026 | 0.135 | 96 | 6 | -0.03 | 0.02 | 0.86 |  |  |
| 5 | 31 | 12 | 0.118 | 0.007 | 0.151 | 171 | 48 | 0.205 | 0.002 | 0.000 | 140 | 14 | 0.13 | 0.00 | 0.07 | "validate" | |
| 6 | 51 | 29 | 0.057 | 0.002 | 0.234 | 289 | 60 | 0.077 | 0.002 | 0.053 | 178 | 11 | 0.00 | 0.00 | 1.00 |  |  |
| 7 | 61 | 32 | -0.001 | 0.001 | 0.984 | 195 | 34 | 0.019 | 0.001 | 0.587 | 178 | 19 | 0.09 | 0.00 | 0.02 | NS (consistent) | |
| 8 | 46 | 36 | -0.043 | 0.001 | 0.177 | 180 | 43 | 0.069 | 0.001 | 0.021 | 29 | 2 | -0.06 | 0.01 | 0.59 |  |  |
| 9 | 80 | 45 | 0.006 | 0.001 | 0.801 | 112 | 23 | -0.045 | 0.001 | 0.190 | 31 | 2 | -0.01 | 0.01 | 0.90 |  |  |
| 10 | 45 | 27 | -0.031 | 0.001 | 0.335 | 115 | 21 | 0.097 | 0.001 | 0.001 |  |  |  |  |  |  |  |
| 11 | 67 | 44 | 0.018 | 0.001 | 0.441 | 172 | 75 | 0.047 | 0.000 | 0.011 | 33 | 2 | 0.04 | 0.01 | 0.58 |  |  |
| 12 | 31 | 15 | -0.021 | 0.001 | 0.434 | 59 | 11 | 0.041 | 0.001 | 0.212 | 91 | 7 | 0.03 | 0.00 | 0.45 |  |  |
| 13 | 46 | 12 | -0.006 | 0.001 | 0.823 | 297 | 35 | 0.022 | 0.000 | 0.213 | 32 | 6 | 0.14 | 0.00 | 0.00 | consistent | |
| 14 | 22 | 10 | 0.045 | 0.001 | 0.103 | 126 | 77 | 0.095 | 0.000 | 0.000 | 28 | 3 | 0.07 | 0.00 | 0.15 | consistent | |

**Supplement Table S13. Replication of Association of Signatures with CVD Risk (FHS Cohort).** LHR= hazard ratios in natural log-scale relative to the referent signature of cluster 1 were estimated using Cox proportional hazard stratified by sex and adjusted by age at exam 7 in FHS offspring and age at exam 20 in the original cohort. Significance is based on p-values from Wald’s test.

|  | G0 (Original generation in FHS) | | | | | Offspring generation | | | | |  | LLFS | | | | | |  | |
| --- | --- | --- | --- | --- | --- | --- | --- | --- | --- | --- | --- | --- | --- | --- | --- | --- | --- | --- | --- |
| cluster | size | events | LogHR | StDev | p-val | size | events | LogHR | StDev | p-val | cluster | size | events | LogHR | StDev | p-val |  | |
| 1 | 159 | 23 |  |  |  | 694 | 53 |  |  |  | 1 | 2262 | 40 |  |  |  |  | |
| 2 | 177 | 31 | -0.30 | 0.09 | 0.33 | 576 | 31 | -0.66 | 0.06 | 0.01 | 2 | 1128 | 11 | -0.67 | 0.12 | 0.05 | validate | |
| 3 | 127 | 23 | 0.11 | 0.03 | 0.54 | 310 | 38 | 0.29 | 0.01 | 0.02 | 3 | 387 | 10 | 0.21 | 0.03 | 0.24 | consistent | |
| 4 | 192 | 41 | -0.03 | 0.01 | 0.75 | 182 | 4 | -0.10 | 0.03 | 0.58 | 4 | 96 | 1 | -0.14 | 0.11 | 0.68 |  | |
| 5 | 31 | 5 | -0.04 | 0.02 | 0.77 | 171 | 18 | 0.07 | 0.01 | 0.37 | 5 | 140 | 6 | 0.28 | 0.01 | 0.01 | consistent | |
| 6 | 51 | 12 | 0.20 | 0.01 | 0.01 | 289 | 78 | 0.29 | 0.00 | 0.00 | 6 | 178 | 12 | 0.28 | 0.00 | 0.00 | validate | |
| 7 | 61 | 7 | -0.02 | 0.01 | 0.80 | 195 | 28 | 0.11 | 0.00 | 0.01 | 7 | 178 | 2 | -0.04 | 0.01 | 0.75 |  | |
| 8 | 46 | 11 | 0.03 | 0.00 | 0.62 | 180 | 28 | 0.08 | 0.00 | 0.03 |  |  |  |  |  |  |  | |
| 9 | 80 | 30 | 0.10 | 0.00 | 0.01 | 112 | 28 | 0.12 | 0.00 | 0.00 | 9 | 31 | 1 | 0.12 | 0.02 | 0.36 |  | |
| 10 | 45 | 3 | -0.05 | 0.01 | 0.50 | 115 | 6 | -0.05 | 0.00 | 0.31 |  |  |  |  |  |  |  | |
| 11 | 67 | 40 | 0.18 | 0.00 | 0.00 | 172 | 88 | 0.24 | 0.00 | 0.00 | 11 | 33 | 1 | 0.09 | 0.01 | 0.37 |  | |
| 12 | 31 | 4 | -0.05 | 0.00 | 0.38 | 59 | 4 | -0.02 | 0.00 | 0.69 | 12 | 91 | 4 | 0.08 | 0.00 | 0.09 |  | |

**Supplement Table S14. Replication of Association of Signatures with Type 2 Diabetes (FHS Cohort).** LHR= hazard ratios in natural log-scale relative to the referent signature of cluster 1 were estimated using Cox proportional hazard stratified by sex and adjusted by age at exam 7 in FHS offspring and age at exam 20 in the original cohort. Significance is based on p-values from Wald’s test.
